# The long antisense non-coding RNA HOXA transcript at the distal tip (LncRNA HOTTIP) in health and disease: a comprehensive review and in silico analysis

**DOI:** 10.1007/s00210-025-04372-9

**Published:** 2025-07-05

**Authors:** Mona G. El-Sisi, Sara M. Radwan, Sameh S. Ali, Mohamed Y. Mostafa, Nadia M. Hamdy

**Affiliations:** 1 Department of Biochemistry, Faculty of Pharmacy, Ain Shams University, Cairo, 11566 Egypt; 2 Research Department, Children’s Cancer Hospital Egypt-57357, Cairo, Egypt; 3 Department of Clinical Oncology, Faculty of Medicine, Ain Shams University, Cairo, 11566 Egypt

**Keywords:** HOTTIP, HOXA transcript at the distal tip, LncRNA, Metastasis, Diagnosis, Prognosis, SNPs

## Abstract

Since lncRNAs are RNA transcripts with more than 200 nucleotides that do not encode proteins, they are functional units themselves. It has been demonstrated that they participate in a variety of biological processes. One particular long noncoding RNA, known as HOXA transcript at the distal tip (HOTTIP), is encoded from a genomic region in the 5′ tip of the HOXA locus. It has been linked to the development and spread of human cancer. A growing body of evidence indicates that dysregulation of HOTTIP is linked to a number of cancers and impacts the prognosis and survival of cancer patients but *what are the normal physiological functions of HOTTIP in non-cancerous tissues*? And *how does the dysregulation of HOTTIP contribute to non-malignant diseases*? These are major questions to be answered. In this study, we provide an overview of the current understanding of the significance of lncRNA HOTTIP and, for the first time, describe its participation in normal physiological function. Moreover, *what are the most critical knowledge gaps in the current understanding of HOTTIP*, and *how can these gaps be addressed experimentally*? These are other questions we aimed to answer, in addition to providing insight into its function in chemotherapeutic resistance and its usefulness in the diagnosis and prognosis of numerous malignancies. Finally, we also show how mutations in this lncRNA, even in a single nucleotide, affect many diseases’ fates including malignancies and other non-malignant ones.

**Supplementary Information:**

The online version contains supplementary material available at 10.1007/s00210-025-04372-9.

## Introduction

### Definition and general roles of lncRNAs

Transcribed RNA molecules without a large open reading frame are known as long noncoding RNAs (lncRNAs), and their length is greater than 200 nucleotides (Johnsson et al. 2014). LncRNAs have a variety of significant roles in disease, such as transcriptional and posttranscriptional control, epigenetic regulation, and more (Guo et al. 2015).

### Description of HOTTIP, its discovery, and its relevance to disease

The lncRNA known as HOXA transcript at the distal tip (HOTTIP) is encoded from a genomic area located at the 5′ tip of the HOXA locus. It is a polyadenylated, spliced, 3764-nucleotide long noncoding RNA. Because of its position in the genome, it has the ability to activate many 5′ HOXA genes (Wang et al. 2011). HOTTIP was discovered through high-throughput sequencing and transcriptome analysis aimed at identifying lncRNAs associated with specific chromosomal regions. Researchers employed RNA sequencing (RNA-seq) and chromatin immunoprecipitation sequencing (ChIP-seq) to explore transcriptional activity and chromatin modifications across the HOXA gene cluster (Ghafouri-Fard et al. 2020). Differential expression analysis revealed that HOTTIP is predominantly expressed in distal tissues, such as the fingers and toes, aligning with its role in spatially regulating the HOXA genes. This tissue-specific expression and its ability to orchestrate chromatin architecture underscore its critical role in limb development and cellular differentiation (Wang et al. 2011). Further studies on its dysregulation have linked HOTTIP to certain cancers, where its aberrant expression influences tumor by modulating key developmental pathways. Researchers have recently discovered a relationship between HOTTIP expression and the overall survival, distant metastasis, lymph node metastasis (LNM), and tumor stage of human cancers (Xie et al. 2015). HOTTIP expression may also have an association with the prognosis and metastasis of human cancers (Fan et al. 2018). Despite its established importance in cancer, HOTTIP’s role extends beyond malignant conditions. Emerging evidence suggests that this lncRNA may participate in normal physiological functions, opening avenues for research into its contributions to non-cancerous diseases (Mao et al. 2019a). Moreover, it has strong connections to the pathophysiology of various communicable and non-communicable diseases, including diabetes mellitus (Cao et al. 2022), Hirschsprung disease (Xie et al. 2015), and pre-eclampsia (Li et al. 2018a) for example.

Additionally, mutations within HOTTIP, including single-nucleotide alterations, have been shown to affect disease progression and therapeutic outcomes, underscoring its clinical significance (Ali et al. 2020; Wang et al. 2018a). We collectively mentioned all the studies concerned with HOTTIP polymorphisms to emphasize the gaps in knowledge that offer exciting opportunities for future research.

### Aim of the review

Unfortunately, HOTTIP’s exact significance in diseases other than cancer is, however, poorly described by the majority of studies that have been published to date as they have focused on its dysregulation and oncogenic roles in cancer (Fan et al. 2018; Deng et al. 2015; Gao et al. 2018a). However, its functions in normal, non-cancerous cells remain largely unexplored well. Thus, in order to clarify regulation, significance, and usefulness of HOTTIP for both cancer and non-cancer diseases; to fill the identified research gaps; and to collectively prepare a comprehensive study to aid in better treatment outcomes, we carried out this updated review.

### The search strategy

A comprehensive search using Google Scholar and PUBMED, two e-databases, was done using the keywords (“HOTTIP” OR “HOXA transcript at the distal tip”) AND (“HOTTIP in Cancer”) AND (“HOXA transcript at the distal tip in Cancer”) AND (“HOTTIP in diseases”) AND (“HOXA transcript at the distal tip in Diseases”) (“in silico”) AND (“SNPs”) AND (“Polymorphism”) AND (“Prognostic marker”) AND (“Diagnostic marker”) AND (“Nanoparticles”) AND (“Exosomes”) on July, 2024. Since, but not limited to, 2014, emphasis has been placed on meta-analyses, randomized clinical trials, systematic and narrative reviews, and original articles.

## Biogenesis and discovery

Through RNA polymerase II-mediated transcription, the two main gene types expressed in human genomes are protein-coding transcription units and non-coding RNA transcription units (Nojima and Proudfoot 2022). Non-coding RNAs can be further subdivided into a large number of long non-coding RNAs (lncRNAs) that are frequently low in abundance and stability, and into relatively abundant structural RNAs such as small nuclear RNAs. Recent research has defined distinct roles for either individual lncRNAs or the lncRNA synthesis process itself, even if transcriptional “noise” may be present in at least some lncRNA synthesis. Notably, lncRNA transcription, processing, and metabolism are regulated in a different way than genes that code for proteins (Nojima and Proudfoot 2022).

In steady-state RNA level analysis, the majority of lncRNAs are frequently undetected due to their extreme instability (Nojima and Proudfoot 2022; Schlackow et al. 2017). In actuality, lncRNA complexity may still be underestimated despite the high number of discovered lncRNAs. Furthermore, few lncRNA transcription units’ 3′ ends have been thoroughly characterized; only a portion of them have functional polyadenylation sites (PAS) (Schlackow et al. 2017; Herman et al. 2022). The RNA exosome complex is the predominant RNA degradation machinery that breaks down the majority of non-polyadenylated lncRNAs quickly (Schlackow et al. 2017; Andersson et al. 2014; Pefanis et al. 2015).

Eleven HOX genes make up the human HOXA locus, which is expressed in a graded manner along body appendages from proximal (near the main body) to distal (appendage tip) (Yang 2024). Anatomically distal human fibroblasts from the hand, foot, or foreskin were the original source of HOTTIP (Lian et al. 2016a). The HOTTIP gene was found at the homeobox A (HOXA) locus (chromosomal locus 7p15.2), which encodes the 3764 bp transcript. Consequently, “HOXA transcript at the distal tip” (HOTTIP) was used to refer to the lncRNA (Wang et al. 2011).

## In silico platforms about HOTTIP

**Fig. 1:**
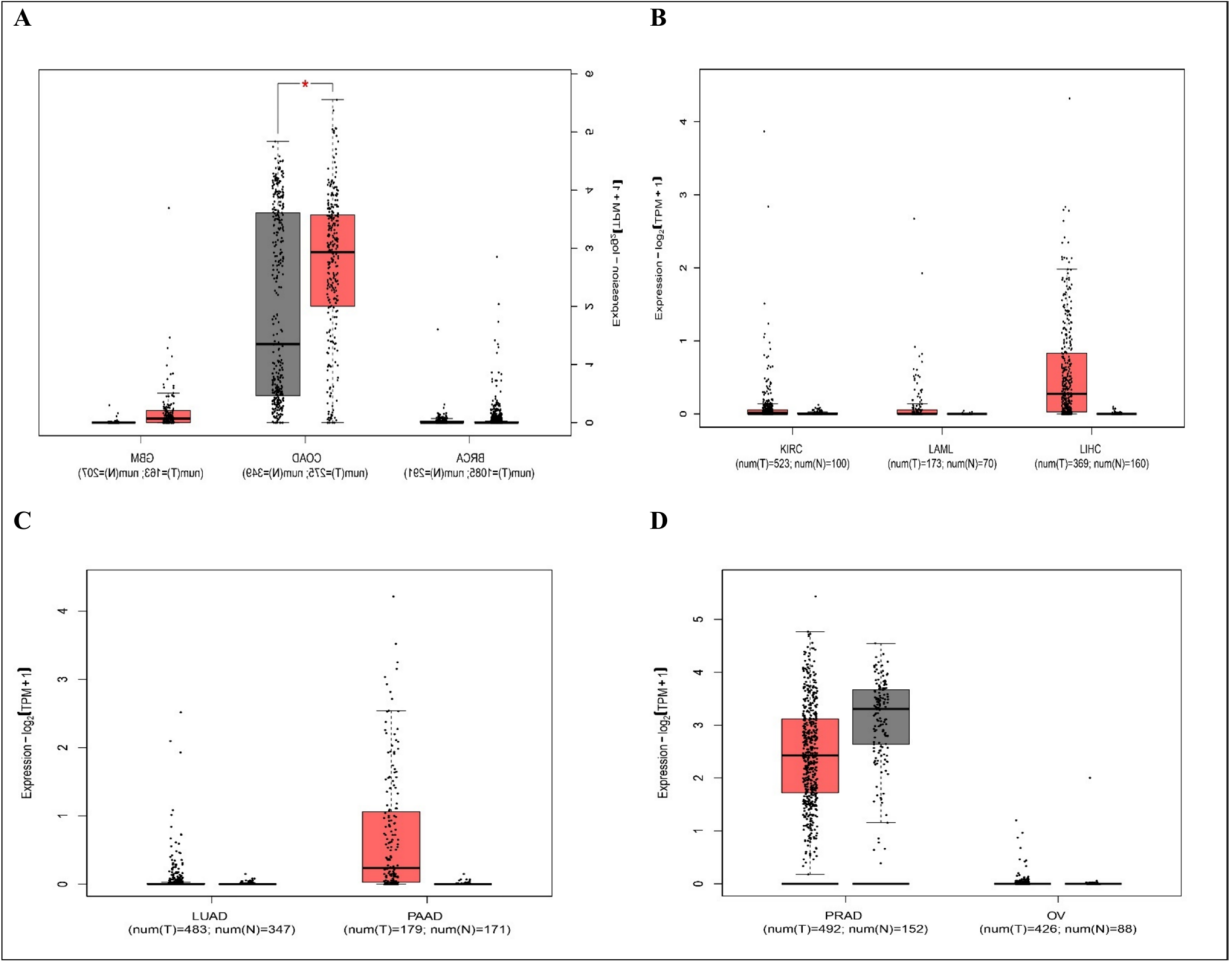
Relative expression between tumor tissue samples and GTex normal tissue samples **A** in BRCA (*n* of tumor samples = 1085, *n* of normal samples = 291), COAD (*n* of tumor samples = 275, *n* of normal samples = 349), GBM (*n* of tumor samples = 163, *n* of normal samples = 207). **B** In KIRC (*n* of tumor samples = 523, *n* of normal samples = 100), LAML (*n* of tumor samples = 173, *n* of normal samples = 70), LIHC (*n* of tumor samples = 369, *n* of normal samples = 160). **C** In LUAD (*n* of tumor samples = 483, *n* of normal samples = 347), PAAD (*n* of tumor samples = 179, *n* of normal samples = 171). **D** In PRAD (*n* of tumor samples = 492, *n* of normal samples = 152), OV (*n* of tumor samples = 426, *n* of normal samples = 88). BRCA, breast invasive carcinoma; COAD, colon adenocarcinoma; GBM, glioblastoma; KIRC, kidney renal clear cell carcinoma; LAML, acute myeloid leukemia; LIHC, liver hepatocellular carcinoma; LUAD, lung adenocarcinoma; OV, ovarian serous cystadenocarcinoma; PAAD, pancreatic adenocarcinoma; PRAD, prostate adenocarcinoma

**Fig. 2:**
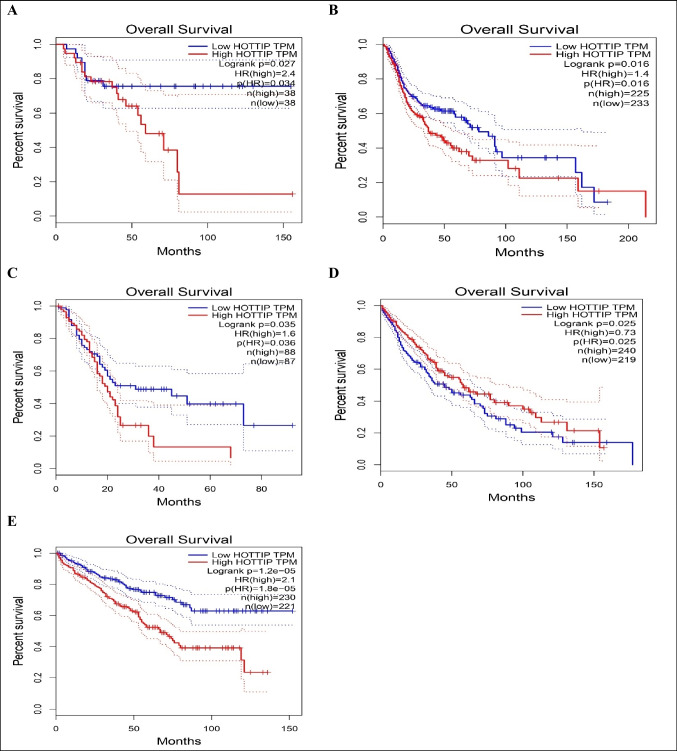
Kaplan–Meier curves showing correlation of low/high expression of lncRNA HOTTIP with overall survival in **A** adrenocortical carcinoma, **B** head and neck squamous cell carcinoma, **C** pancreatic adenocarcinoma, **D** lung squamous cell carcinoma, **E** kidney renal clear cell carcinoma

We used Gene Expression Profiling Interactive Analysis2 (GEPIA2) database (http://gepia2.cancer-pku.cn/#index) (Tang et al. 2017), which is a web server that enables gene expression analysis based on tumor and normal samples from the TCGA and the GTEx databases. We used GEPIA database (a) to compare HOTTIP expression between top 10 most common cancer types and paired normal tissue as shown in Fig. 1, (b) to explore the correlation between HOTTIP expression and overall survival in these cancers as seen in Fig. 2, and (c) to identify top 5 genes that have similar expression pattern to HOTTIP in the mentioned cancer types and this can be seen in supplementary table (S1). (d) To explore the correlation between HOTTIP and common genes related to cancer hallmarks (invasion, metastasis, etc.) as shown in supplementary figures (S1), (S2), (S3), and (S4).

This diagram shows that HOTTIP high expression in adrenocortical, head and neck squamous cell, pancreatic and kidney renal clear cell carcinomas is significantly associated with lower overall survival. On the other hand, in lung squamous cell carcinoma, low HOTTIP expression is associated with lower overall survival.

As shown in these diagrams, significant differences between tumor and normal samples are observed only in COAD. On the other hand, there’s no significant difference between tumor and normal samples and this may be attributed to low sample sizes which require more studies on larger sample sizes.

We also used *starBase or Encyclopedia of RNA Interactomes* (*ENCORI*) (https://rnasysu.com/encori/index.php) (Li et al. 2014) web server, through which we presented the interaction network of lncRNA-RNA identified from high-throughput sequencing data of RNA-RNA interactome as shown in Table 1.

**Table 1:** The HOTTIP-RNA Interaction Network in human

Gene name	PairGeneID	Pair gene name	Pair gene type	Exp num	Seq type num	Total reads num	Free energy	Align score (Smith-Waterman)
HOTTIP	ENSG00000086589	RBM22	protein_coding	1	1	1	− 26.1	13
	ENSG00000087095	NLK	protein_coding	1	1	1	− 19.9	12
	ENSG00000115207	GTF3C2	protein_coding	1	1	1	− 40.3	15.5
	NR_004435.1	SNAR-A1	SNAR_snRNA	2	1	11	− 22.1	15.5

*RBM22* RNA binding motif protein 22, *NLK* nemo-like kinase, *GTF3C2* general transcription factor IIIC subunit 2, *SNAR-A1* small NF90 (ILF3)–associated RNA A1, *snRNA* small nuclear RNA

As stated in Table 1, Exp Num (experimental number) indicates the number of experiments or datasets supporting this interaction, Seq Type Num (sequence type number) refers to the number of distinct sequence types used to verify this interaction. Moreover, Total Reads Num represents the total number of RNA sequencing reads that confirm this interaction and Free Energy refers to the binding energy (measured in kcal/mol) between HOTTIP and the interacting partner. Negative values indicate a stable interaction; the more negative the value, the stronger the interaction.

Align Score (Smith-Waterman) indicates the alignment score generated by the Smith-Waterman algorithm, which measures the quality of the sequence alignment between HOTTIP and the interacting RNA. Higher scores indicate better alignment. We can conclude that HOTTIP interacts with protein-coding genes (e.g., RBM22, NLK, GTF3C2) and noncoding RNAs (e.g., SNAR-A1). The stability of these interactions varies, with free energy values ranging from − 19.9 to − 40.3 kcal/mol. Finally, SNAR-A1 is a small nuclear RNA interacting with HOTTIP, while others are protein-coding genes involved in cellular processes.

**Fig. 3:**
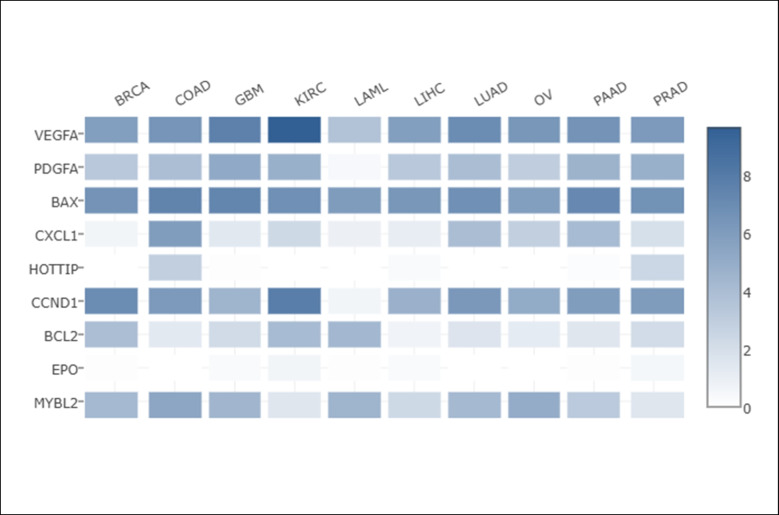
Heatmap showing relative expression of HOTTIP compared to some cancer hallmarks’ markers in different cancers. CCND1, Cyclin D1; BAX, Bcl-2-associated X-protein; BCL2, B cell lymphoma 2; CXCL1, C-X-C motif chemokine ligand 1; HOTTIP, HOXA transcript at the distal tip; EPO, erythropoietin; MYBL2, myelobglioblastoma like 2; PDGFA, platelet-derived growth factor subunit A; VEGFA, vascular endothelial growth factor A; BRCA, breast invasive carcinoma; COAD, colon adenocarcinoma; GBM, glioblastoma; KIRC, kidney renal clear cell carcinoma; LAML, acute myeloid leukemia; LIHC, liver hepatocellular carcinoma; LUAD, lung adenocarcinoma; OV, ovarian serous cystadenocarcinoma; PAAD, pancreatic adenocarcinoma; PRAD, prostate adenocarcinoma

Moreover, we used GEPIA2 webserver to get a heatmap concerning the multiple expression of HOTTIP compared to the cancer hallmarks’ markers—previously mentioned in figures (S1, S2, S3 and S4)—in different cancers as shown in Fig. 3.

We also used *UCSC Xena* web server (https://xena.ucsc.edu/#analysis) (Goldman et al. 2020) to get heatmaps showing HOTTIP’s gene expression in different common cancers in Supplementary Fig. S5.

## HOTTIP regulation

It was found that lncRNAs perform a variety of regulatory duties, such as translation, chromatin remodeling, post-translational protein modification, protein trafficking, and signaling among cells. They also regulate transcription through interactions with proteins and RNAs (Choudhuri 2023). For HOTTP, the direct interaction with WD repeat-containing protein 5 (WDR5) has been demonstrated. This interaction induces an open deoxy nucleic acid (DNA)-chromatin configuration to target WDR5/mixed lineage leukemia (MLL) complexes that drive histone H3 lysine 4 trimethylation, hence controlling the transcription of 5′ end HOXA locus genes (Wang et al. 2011). This was concluded by a mechanistic study by Wang et al. where they used many techniques to prove that interaction (Wang et al. 2011). They used techniques like RNA immunoprecipitation (RIP) to show direct interaction between HOTTIP and WDR5 and chromatin isolation by RNA purification (ChIRP) technique to map where HOTTIP binds across the genome and shows colocalization with WDR5/MLL. Moreover, this study utilized RNA pull-down assays to confirm specific binding of HOTTIP to WDR5 and loss-of-function studies (siRNA knockdown) to demonstrate that depletion of HOTTIP leads to decreased H3K4me3 and reduced expression of HOXA genes as well (Wang et al. 2011).

Research has indicated that the *HOTTIP* gene regulates the expression of the HOXA locus and is situated close to the *HOXA13* gene. Numerous transcription factors essential in embryogenesis and critical cellular processes are encoded by this HOXA locus (Lin et al. 2017). Furthermore, it acts as a competing endogenous RNA (ceRNA) of miR-30b which in turn eliminates miR-30b level regulating *HOXA13* expression (Lin et al. 2017). Additionally, some lncRNAs are involved in translation, mRNA degradation, and protein kinetics (Ye et al. 2019). Examples of these include the homeobox (Hox) antisense intergenic RNA (HOTAIR) and HOTTIP (Ye et al. 2019; Liu et al. 2020a).

Furthermore, determining whether the RNA molecule itself or the transcription mechanism is responsible for lncRNA function remains challenging. There have been questions about the shortcomings and differences in the different approaches taken in the research of lncRNA function (Bassett et al. 2014; Portoso et al. 2017; Selleri et al. 2016). Beyond that, not much is understood about lncRNAs’ upstream controls. The expression of the lncRNA HOTTIP, which is located at the 5′ end of the HOXA locus, is specifically regulated by the short isoform (p52) of PC4 and SF2 interacting protein 1 (Psip1), a transcriptional co-activator known to be involved in connecting transcription to RNA processing, according to a study by Pradeepa and her colleagues (Pradeepa et al. 2017). Besides, in order to cooperatively maintain the chromatin modifications of HOXA genes and thereby coordinate the transcriptional activation of distal HOXA genes in human foreskin fibroblasts, HOTTIP physically associates with the CCCTC-binding factor (CTCF), which functions as an insulator by arranging the HOXA cluster into disjoint domains (Wang et al. 2018b).

**Fig. 4:**
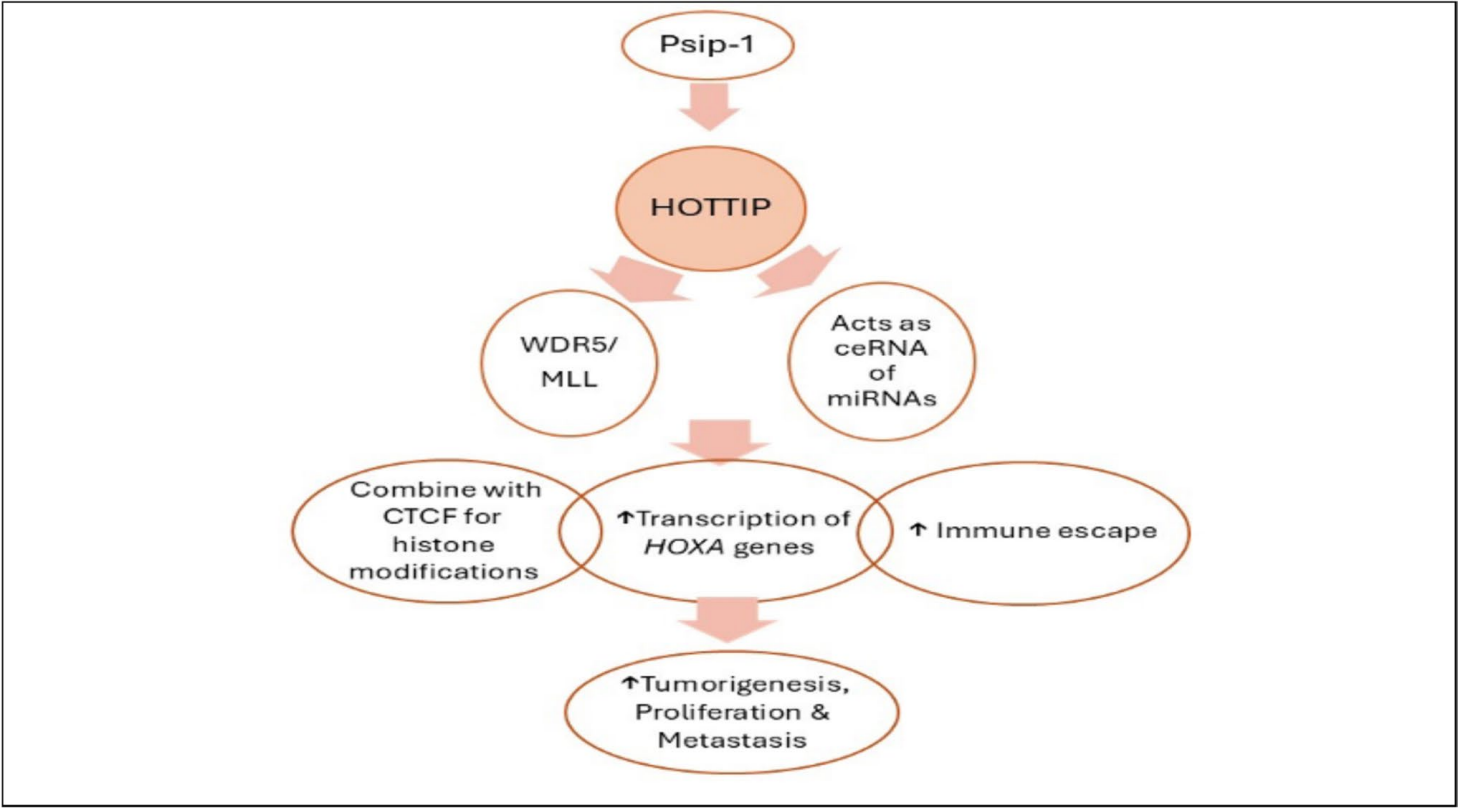
Regulation of HOTTIP (upstream/downstream pathways). Psip1, PC4 and SF2 interacting protein 1; WDR5, WD repeat-containing protein 5; MLL, mixed lineage leukemia; ceRNA, competing endogenous RNA; HOXA, homeobox A; HOTTIP, HOXA transcript at the distal tip; CTCF, CCCTC-binding factor

A summary of regulations related to HOTTIP is summarized in Fig. 4.

## HOTTIP and other HOX-related lncRNAs

### HOXA families

The HOX gene clusters (HOXA, HOXB, HOXC, HOXD) encode transcription factors critical for anterior–posterior body patterning and organogenesis (Wang et al. 2011). In addition to protein-coding HOX genes, these clusters produce lncRNAs, notably HOTAIR (from the HOXC cluster) and HOTTIP (from the HOXA cluster). These lncRNAs regulate gene expression via epigenetic modification, influencing development, stem cell behavior, and disease (especially cancer) (Wang et al. 2011).

### Mechanisms of action: chromatin remodeling and histone modification

Both HOTTIP and HOTAIR act as scaffolds that recruit chromatin-modifying complexes to regulate gene expression epigenetically. HOTTIP recruits WDR5/MLL complex via direct binding, promotes H3K4me3, an epigenetic modification to the DNA packaging protein histone H3 containing the tri-methylated lysine 4, at 5′ HOXA genes (e.g., HOXA13, HOXA11) and acts in cis (regulates nearby HOXA genes). On the other hand, HOTAIR binds Polycomb Repressive Complex 2 (PRC2) via its 5′ domain, and induces H3K27me3 and H3K4me2, an epigenetic modification to the DNA packaging protein histone H3 containing the di-methylated lysine 4, demethylation silencing genes and acts in trans, especially repressing HOXD locus and genes on other chromosomes (Gupta et al. 2010).

### Functional overlap

Table 2 shows functional overlap while Table 3 shows functional divergence between HOTTIP and HOTAIR as a representative example on HOXA genes.

**Table 2:** Functional overlap between HOTTIP and HOTAIR (Ghafouri-Fard et al. 2020; Nazari et al. 2024)

Area	Overlap
Epigenetic remodeling	Both recruit histone-modifying enzymes to regulate gene expression (activating or repressing)
Developmental regulation	Both regulate key HOX genes crucial for embryonic patterning and organogenesis
Cancer progression	Both are aberrantly upregulated in various cancers (e.g., breast, liver, colorectal), affecting proliferation, metastasis, and EMT
Scaffold function	Both serve as platforms for multiple protein interactions (modular architecture)
3D genome organization	Emerging data suggests both may help mediate chromosomal looping and domain interactions, e.g., HOTTIP is involved in chromatin looping with WDR5, while HOTAIR may interact with chromatin boundary elements

*EMT* epithelial–mesenchymal transition, *HOTTIP* HOXA transcript at the distal tip, *WDR5* WD repeat domain 5, *HOTAIR* HOX transcript antisense RNA

**Table 3:** Functional divergence between HOTTIP and HOTAIR (Ghafouri-Fard et al. 2020; Nazari et al. 2024)

Feature	HOTTIP	HOTAIR
Targeting range	Primarily local (HOXA cluster)	Long-range (HOXD and genome-wide)
Epigenetic effect	Activation (via H3K4me3)	Repression (via H3K27me3 and H3K4 demethylation)
Pathways involved	Wnt/β-catenin, TGF-β signaling	PRC2-mediated silencing, EMT regulation
Disease relevance	Strongly linked to limb malformations, cancer, fibrosis	Strongly implicated in metastasis, poor prognosis in breast and colorectal cancer

*PRC2* polycomb repressive complex 2, *Wnt* wingless-related integration site, *TGF-β* transforming growth factor beta, *EMT* epithelial–mesenchymal transition

## HOTTIP in normal cells

The majority of research on HOTTIP has focused on its dysregulation and oncogenic roles in cancer. However, its functions in normal, non-cancerous cells remain largely unexplored.

### HOTTIP in cartilages

For example, it was found that HOTTIP may function as RNA scaffolds or traps during osteogenic development, adding an additional layer of post-transcriptional regulation (Ye et al. 2019; Liu et al. 2020a). Additionally, in human primary chondrocytes, HOTTIP elevated levels of C–C motif chemokine ligand 3 (CCL3) and negatively regulated micro-RNA (miR)−455-3p (Mao et al. 2019a). Mechanistic analyses revealed that HOTTIP acted as a ceRNA for miR-455-3p, which raised the expression of CCL3. In turn, downregulation of HOTTIP boosted the expression of genes relevant to cartilage (Mao et al. 2019a).

Additionally, it was shown that the normal physiological function of the intervertebral disc can be maintained by activating HOTTIP transcription, which in turn suppresses the degeneration of chondrocyte-like cells and stimulates their proliferation (Hao et al. 2024). This prevents the breakdown of the intervertebral disc matrix. Thus, higher HOTTIP levels appear necessary for maintaining healthy cartilage. Another study by Kim et al. showed that by modifying integrin-α1 transcriptionally via HOXA13 or epigenetically via DNA methyltransferase-3B (DNMT-3B), HOTTIP regulates cartilage growth and degeneration (Kim et al. 2013).

### HOTTIP in nervous system

LncRNAs are essential for many physiological functions, yet little is known about how they regulate adult brain and neural stem cells (NSCs). Ten lncRNAs, including HOTTIP, are expressed during the differentiation of murine NSCs, according to research by Carelli et al. (Carelli et al. 2019). This clearly suggests that lncRNAs play a crucial synergistic function in neural stem cells’ fate. Furthermore, through sponging miR-335, lncRNA HOTTIP overexpression works as a neuroprotective factor to combat inflammation in the hippocampal regions, contributing to neuroprotection (Carvalho et al. 2023). Additionally, through Reelin signaling, the WDR5-HOTTIP Histone Modifying Complex modulates dendritic polarity and neural migration in pyramidal neurons (Ka et al. 2022).

### HOTTIP in bone development

It is widely recognized that the HOXA gene cluster plays a crucial role in mediating positional identity in the skeletal system, with the expression of distinct orthologues resulting in distinct locational phenotypes of vertebrate bones revealing HOTTIP and all HOXA members’ roles in the normal healthy bone function (Silva et al. 2019). A study conducted on bone marrow mesenchymal stem cells (BMSCs) revealed that during the process of osteoblast development and angiogenesis, HOTTIP was significantly upregulated together with the osteogenic transcriptional factors (Zeng et al. 2022). In addition, HOTTIP stabilized distal-less homeobox 2 (DLX2) through its interaction with TATA-box binding protein–associated factor 15 (TAF15) protein, which in turn accelerated osteogenic differentiation and angiogenesis and upregulated osteogenic and angiogenic-related gene expression (Zeng et al. 2022). Likewise, Liu et al. demonstrated that HOTTIP is a conservative long noncoding RNA that is necessary for BMSCs osteogenic development. By interacting with WDR5 and upregulating the expression of the β-catenin gene, HOTTIP enhances osteogenic differentiation and activates the Wingless-related integration site (Wnt)/β-catenin signaling pathway (Liu et al. 2020a).

### HOTTIP in fertility

The role of lncRNAs in the cellular response to UV therapy remains largely unexplored, even though they are increasingly recognized as key regulators of various cellular processes (Liang and Hu 2019). UV-induced G2/M-phase arrest and early apoptosis are controlled by HOXA13 and lncRNA-HOTTIP. Additionally, in UV-irradiated spermatogonia germ cells, lncRNA-HOTTIP can upregulate the expression of p53 via HOXA13. Furthermore, both in vitro and in vivo, p53 can control the expression of lncRNA-HOTTIP and HOXA13. This unexpectedly demonstrated the function of lncRNA-HOTTIP in spermatogenic cells’ repair of UV light-induced DNA damage (Liang and Hu 2019).

### HOTTIP in pancreas

Regarding pancreatic cells, insulin secretion and the islet β cell cycle are inhibited by downregulating HOTTIP through the mitogen-activated protein kinase (MAPK)/MAPK kinase (MEK)/extracellular signal–regulated kinase (ERK) pathway in animal model (Xu et al. 2018) revealing its role in keeping normal blood glucose balance. Interestingly, the pro-angiogenic lncRNA HOTTIP was one of the lncRNAs that have been the subject of research on its function in controlling ocular angiogenesis (Gandhi et al. 2024). Talking about its role and mode of action in vascular oculopathies can provide new perspectives on it as a potential therapeutic target.

### HOTTIP in diseases

Negative consequences on human health have also been linked to dysregulation of long noncoding RNAs (Quagliata et al. 2014). It is becoming more widely accepted that lncRNA expression profile data must be carefully analyzed in order to ascertain whether changed expression of these molecules can serve as biomarkers for toxicity or unfavorable effects for human health (Choudhuri 2023).

### HOTTIP in non-cancerous diseases

It is well-established that HOTTIP plays many roles in various types of cancers. However, its role in other diseases is still not well-elucidated.

#### HOTTIP in diabetes

Diabetes, an endocrine system disorder identified by abnormally elevated blood glucose levels, is one of the most prevalent and rapidly expanding diseases globally, expected to impact 693 million individuals by 2045 (Cho et al. 2018). Early genetic research had severe problems that hampered genetic finding, even though family studies have shown clear genetic components to diabetes and associated consequences (Cole and Florez 2020). In mice with normal islet tissues, HOTTIP was found to be increased, whereas in animals with diabetes islet tissues, it was downregulated (Xu et al. 2018). Insulin secretion was reduced by HOTTIP inhibition. Additionally, the downregulation of HOTTIP decreased the expression of CyclinD1, CyclinD2, CyclinE1, and CyclinE2 and impeded cell proliferation. Furthermore, following HOTTIP knockdown, islet β cells were stopped in the G0/G1 phase. These findings suggested a potential link between the MEK/ERK pathway and HOTTIP’s role in regulating insulin production and the cell cycle in islet β cells (Xu et al. 2018).

Additionally, through the p38-mitogen-activated protein kinase (p38-MAPK) pathway, HOTTIP improves diabetic retinal microangiopathy (Sun and Liu 2018). HOTTIP is anticipated to be a promising target for treating diabetic microangiopathy. The retinas of diabetic rats and mice showed significantly higher levels of HOTTIP expression. Downregulating HOTTIP can lessen the decline in visual function and apoptosis of retinal cells caused by diabetes.

While direct studies on HOTTIP in diabetes mellitus patient populations are limited, recent research has explored HOTTIP’s involvement in diabetic complications, particularly diabetic retinopathy. A study investigated the expression levels of lncRNAs, including HOTTIP, in the serum of patients with varying stages of diabetic retinopathy (Li et al. 2024a). The findings indicated that HOTTIP expression increased with the severity of diabetic retinopathy, suggesting its potential as a biomarker for this complication.

Diabetic nephropathy (DN) is another diabetic consequence that needs to be managed. LncRNAs are involved in the pathophysiology of several disorders, including DN. The specific mechanism is still mostly unclear, though (Allison 2016). In DN mice and mouse mesangial cells (MMCs) treated with drugs causing hyperglycemia, HOTTIP was elevated and miR-455-3p was downregulated (Zhu et al. 2019). Elevating the glucose level could lead to increased fibrosis-related protein expression, cell proliferation, inflammation, and activation of the Wnt/β-catenin/cyclin D1 pathway. On the other hand, silencing HOTTIP would reverse all of these effects. As a primary target of HOTTIP, miR-455-3p’s inhibitor was able to mitigate the effects of HOTTIP knockdown on the expression of proteins linked to fibrosis, inflammation, and cell proliferation. These findings suggest that the lncRNA HOTTIP/miR-455-3p/Wnt axis plays a role in the development of extracellular matrix (ECM) buildup, inflammation, and cell proliferation in DN (Zhu et al. 2019).

Regarding gestational diabetes mellitus (GDM), a recent study showed that in GDM mice, HOTTIP levels were reduced while miR-423-5p was increased (Cao et al. 2022). Overexpressed HOTTIP or silenced miR-423-5p improved insulin regulation and increased the expression of glucose transporter 2 (GLUT2). It also reduced the levels of phosphoenolpyruvate carboxykinase (PEPCK) and glucose-6-phosphatase (G-6-Pase), minimized damage to pancreatic tissues, and prevented pancreatic cell apoptosis. The overexpression of miR-143-5p reversed the repressive effects of increased HOTTIP on GDM. HOTTIP used a miR-423-5p sponge. By modifying miR-423, HOTTIP improves hepatic gluconeogenesis and insulin resistance in GDM mice (Cao et al. 2022).

#### HOTTIP in heart diseases

Acute myocardial infarction (AMI) is one of the many human disorders on which lncRNAs have been the subject of increasing research (Jarroux et al. 2017; Wang et al. 2019a). While lncRNA HOTTIP has been shown to be involved in coronary artery disorders, its precise function and mode of action in acute myocardial infarction are yet unknown. According to a recent study, hypoxia-induced cardiomyocytes and the ischemic myocardium of MI mice both have considerably higher levels of HOTTIP (Wang et al. 2022). Furthermore, in vitro, HOTTIP knockdown significantly increased cardiomyocyte development and decreased cardiomyocyte death. By focusing on the miR-92a axis, the researchers showed that HOTTIP downregulation inhibited the course of AMI and proposed HOTTIP as a possible therapeutic target for AMI (Wang et al. 2022).

The most frequent clinical consequence of sepsis is cardiac dysfunction (Habimana et al. 2020; Manetti et al. 2020). The clinical significance of HOTTIP in the start of sepsis and the development of cardiac dysfunction was investigated in the study by Fan et al. (Fan et al. 2022). Sepsis patients with elevated serum HOTTIP levels underwent testing. Additionally, they demonstrated how serum HOTTIP might predict the onset of cardiac dysfunction in sepsis patients. Furthermore, HOTTIP has the ability to independently influence the onset of cardiac dysfunction. HOTTIP knockdown in vitro reversed lipopolysaccharide (LPS)-induced cell death and excessive production of interleukin-6 (IL-6). They came to the conclusion that HOTTIP, perhaps because of its role in LPS-induced myocardial apoptosis and inflammation, is closely associated with the state of patients with sepsis and the emergence of cardiac dysfunction (Fan et al. 2022).

The primary cause of heart disease and stroke is atherosclerosis (Chen et al. 2016a; Han et al. 2014). The pathophysiology and development of atherosclerosis are yet unknown, though. The onset and pathological course of atherosclerosis are significantly influenced by endothelial cells’ migration and proliferation (Yu and Li 2014; Zhang et al. 2015a). Researchers found that the expression level of HOTTIP was higher in tissues affected by coronary artery disease (CAD) than in normal arterial tissues in a recent study (Liao et al. 2018). By activating the Wnt/β-catenin pathway, ectopic expression of HOTTIP boosted endothelial cell motility and proliferation. The mechanism that Guo et al. highlighted is another one that has to be addressed. They discovered that by controlling the miR-490-3p/high mobility group box 1 (HMGB1) axis and triggering the phosphoinositide 3-kinase (PI3K)/protein kinase B (AKT) signaling cascade, HOTTIP knockdown suppresses cell proliferation and migration (Guo et al. 2020).

Primary aldosteronism (PA) has been linked to cardiovascular outcomes such as CAD, congestive heart failure (CHF), and stroke, according to observational studies (Inoue et al. 2020; Monticone et al. 2018). However, the dearth of evidence from randomized controlled trials on this subject makes proving causality difficult (Davies et al. 2018; Sanderson et al. 2022). Thus, using a genome-wide association and Mendelian randomization study, some researchers sought to determine the causal relationship between PA and the risk of developing CAD, CHF, and stroke. They found that seven genetic loci, including HOTTIP, were significantly associated with the risk of PA. Together, they came to the conclusion that it may be a predictor of PA and, by extension, a number of cardiovascular diseases (Inoue et al. 2024).

#### HOTTIP in bone disorders

A build-up of pro-inflammatory factors is the hallmark of acute gouty arthritis (AGA). One of them, according to Shao et al.’s hypothesis, is HOTTIP (Shao et al. 2023). When compared to controls, it was statistically overexpressed in AGA patients, but miR-101-3p decreased. Patients with AGA can be clearly identified from healthy controls using serum HOTTIP. By means of miR-101-3p, HOTTIP promotes inflammation and functions as a novel biomarker for diagnosis. Rheumatoid arthritis (RA) etiology may be significantly influenced by certain proteins and lncRNAs, according to accumulating data. In RA synovial fibroblasts (RASFs), HOTTIP was discovered to be significantly expressed while Secreted Frizzled-Related Protein 1 (SFRP1) protein was hypermethylated (Hu et al. 2020). Inhibiting RASF proliferation, invasion, and migration while promoting apoptosis was achieved by either overexpressing SFRP1 or silencing HOTTIP. Furthermore, SFRP1 elevation or HOTTIP silencing prevented RA from progressing in vivo. Thus, we could conclude that HOTTIP silencing in RA via SFRP1 promoter demethylation has anti-inflammatory properties. Liu et al. investigated lncRNA HOTTIP expression in peripheral blood mononuclear cells from RA patients (Wei et al. 2023).They found an increased HOTTIP expression in ~ 60 RA patients vs. controls and also showed association with inflammatory cytokine levels (e.g., IL-6, TNF-α). It is worth noting that expression levels were correlated with disease activity scores. These results validate HOTTIP as a potential anti-arthritis target.

The method by which HOTTIP affects fibroblast-like synoviocytes’ (FLS) inflammatory response and apoptosis on a RA rat model was examined in a study (Wang et al. 2024). The synovial tissues of RA mice exhibited increased expression of HOTTIP. Reduced clinical scores, inflammatory infiltration, synovial hyperplasia, tartrate-resistant acid phosphatase (TRAP) activity, and enhanced bone mineral density (BMD) were all observed when HOTTIP was inhibited. It was stated that HOTTIP inhibited the growth of RA-FLS while triggering RA-induced cell death and inflammation (Wang et al. 2024). According to a different study, HOTTIP may exacerbate RA by inciting inflammation, which may be connected to the control of miR-1908-5p production and signal transducer and activator of transcription 3 (STAT3) signaling pathway (Yao et al. 2021a). These findings showed that RA treatment using HOTTIP control would be a viable approach.

Ankylosing spondylitis (AS) is a long-term inflammatory condition primarily affecting the spinal ligaments, vertebrae, and sacroiliac joint (Kang et al. 2014; Saha et al. 2021). For a chronic innate immune response in AS, genetic research and the effects of local tissue variables, such as biomechanical stress and bacterial products, are crucial (Mauro et al. 2021; Yang et al. 2016). In AS fibroblast-like synoviocytes (ASFLSs) and AS synovial tissues, HOTTIP was increased. Inhibited HOTTIP enhanced the pathogenic alterations and encouraged synoviocyte death in the synovial tissues of mice, while also limiting the proliferation and differentiation of ASFLSs (Wei et al. 2023). This shows that by inhibiting the growth and differentiation of ASFLSs, decreased HOTTIP slows the course of AS.

Another concerning bone disorder is osteoarthritis (OA). OA cartilage tissues and chondrocytes exhibited markedly increased expression of the lncRNA HOTTIP (Mao et al. 2019a). In vitro, chondrocytes with interleukin-1β (IL-1β) showed enhanced expression of HOTTIP. HOTTIP knockdown increased the expression of genes related to cartilage such as A disintegrin and metalloprotease with thrombospondin motifs 4 (ADAMTS-4), A disintegrin and metalloprotease with thrombospondin motifs 5 (ADAMTS-5), matrix metallopeptidase 3 (MMP3), and matrix metallopeptidase 13 (MMP13). On the other hand, overexpression of HOTTIP decreased the expression of these genes. According to mechanistic studies, HOTTIP acted as a ceRNA for miR-455-3p. When considered collectively, the HOTTIP/miR-455-3p ceRNA regulation network is crucial to the pathophysiology of OA and raises the possibility that HOTTIP could be a target for OA treatment (Mao et al. 2019a).

Additionally, in OA cartilage tissues, there was an upregulation of HOTTIP expression and a downregulation of miR-663a (He et al. 2021a). In OA cartilage model cells, knockdown of HOTTIP resulted in decreased proliferation and apoptosis, whereas overexpression of HOTTIP enhanced proliferation and lowered apoptosis. Furthermore, HOTTIP may bind as a competitive endogenous RNA to miR-663a. The effect of HOTTIP knockdown on the proliferation and death of OA cartilage model cells may be mitigated by inhibiting miR-663a expression. Finally, suppression of miR-663a boosted OA cell proliferation and decreased cell apoptosis. Thus, HOTTIP regulates miR-663a to contribute to the progression of osteoarthritis, according to He and his colleagues (He et al. 2021a).

Functionally, forkhead box O3 (FOXO3) enhanced the production of the miR-615-3p target gene, collagen type II alpha 1 chain (COL2A1), and transcriptionally activated HOTTIP (Hao et al. 2024). This allowed HOTTIP to bind to miR-615-3p more competitively. As a result, intervertebral disc degeneration (IDD) developed later than expected because cell proliferation was stimulated, and cell death was reduced.

#### HOTTIP in liver diseases

For both men and women, hepatocellular carcinoma (HCC) is the second most frequent cause of cancer-related mortality. It arises as a result of a gradual process that often begins with the development of premalignant lesions from morphologically identifiable low- and high-grade dysplastic nodules inside a cirrhotic liver (Abaza et al. 2023; El-Aziz et al. 2023; El-Derany et al. 2016; Eldosoky et al. 2023). Understanding the pathophysiology of HCC may be aided by the pathways’ multifactorial features, which highlight the significance of epigenetic factors such as miRs and lncRNAs (Eldosoky et al. 2023; Hammad et al. 2023).

HOTTIP is commonly known as an oncogenic long noncoding RNA that has been demonstrated to be upregulated in a range of malignancies (Ghafouri-Fard et al. 2020). It is unclear, therefore, how HOTTIP functions biologically in liver fibrosis. A study found that activated hepatic stellate cells (HSCs) exhibited a particular overexpression of HOTTIP while its knockdown inhibited HSC activation and proliferation (Zheng et al. 2019). Subsequent investigation showed that HOTTIP induced the expression of serum response factor (SRF), an endogenous RNA that competes with miR-150 to activate HSCs. When combined, that offers a new signaling cascade in liver fibrosis that involves HOTTIP-miR-150-SRF (Zheng et al. 2019). A different study revealed that HOTTIP contributes to the development of liver fibrosis by increasing the amount of transforming growth factor beta receptor (TGFBR) 1 and TGFBR2, which in turn promotes HSC activation through downregulating miR-148a (Li et al. 2018b).

Hepatitis B, an infection that progresses to acute-chronic hepatitis, severe liver failure, and mortality, is still a global issue (Guvenir and Arikan 2020). Hepatitis B is an inflammation of the liver caused by the hepatitis B virus (HBV). LncRNAs have been linked to HBV-related diseases and infections, although the underlying mechanisms are yet unknown, up to Zhao et al.’s study, which examined HOTTIP’s role in HBV replication. They discovered that the cAMP-responsive element-binding protein 1 (CREB1)-HOTTIP-HOXA13 axis is activated by HBV DNA polymerase (DNA pol), which inhibits HBV replication (Zhao et al. 2021). These results provide insight into the process by which HBV inhibits replication to support long-term infection. Furthermore, the expression of HOTTIP was significantly higher in resolved HBV patients at two time points (initial diagnosis and after 12 months of treatment) compared to the control group (Yılmaz Susluer et al. 2018). These results suggest that HOTTIP may serve as a valuable prognostic biomarker for HBV patients.

Patients with HBV can benefit from tenofovir alafenamide (TAF) as a therapy. It is still unknown, nevertheless, exactly how TAF’s antiviral action is achieved (Sax et al. 2015). Researchers have examined the antiviral potential of exosomes made from HBV patients’ sera who received TAF treatment (Exo-serum) and TAF-treated macrophages (MP) (Exo-MP(TAF)). The outcomes showed that Exo-serum and Exo-MP(TAF) both exhibited strong antiviral activity, as evidenced by the considerable downregulation of HBV DNA, covalently closed circular DNA, hepatitis B surface antigen, and hepatitis B e antigen (Liu et al. 2021). RNA sequencing (RNAseq) analysis demonstrated a substantial upregulation of lncRNA HOTTIP in Exo-serum. Moreover, lncRNA HOTTIP knockdown had the opposite effects from Exo-MP(TAF) in terms of its antiviral activity. When considered collectively, these findings point to the necessity of exosomal lncRNA HOTTIP for TAF’s antiviral action and offer an entirely new perspective on the exosome-mediated process driving HBV infection (Liu et al. 2021).

One of the most prevalent chronic liver disorders, hepatitis C virus (HCV) chronic infection affects ranging from 1% to over 3% of the global population (Blach et al. 2022; Stroffolini and Stroffolini 2024). HCV core protein’s functional significance in HCC has been closely studied as it builds the HCV capsid, takes part in HCV immune escape, is resistant to medication, and is associated with liver fibrosis (El-Mesallamy et al. 2011). According to recent data, there is potential that the core expression of the HCV not only causes downregulated miR204 expression, which in turn increases the expression of HOTTIP, but also activates the transforming growth factor β receptor–associated protein 1 (TGFBRAP1) signaling pathway, which is linked to the promotion of HCV’s carcinogenic progression (Wang et al. 2018c).

**Fig. 5:**
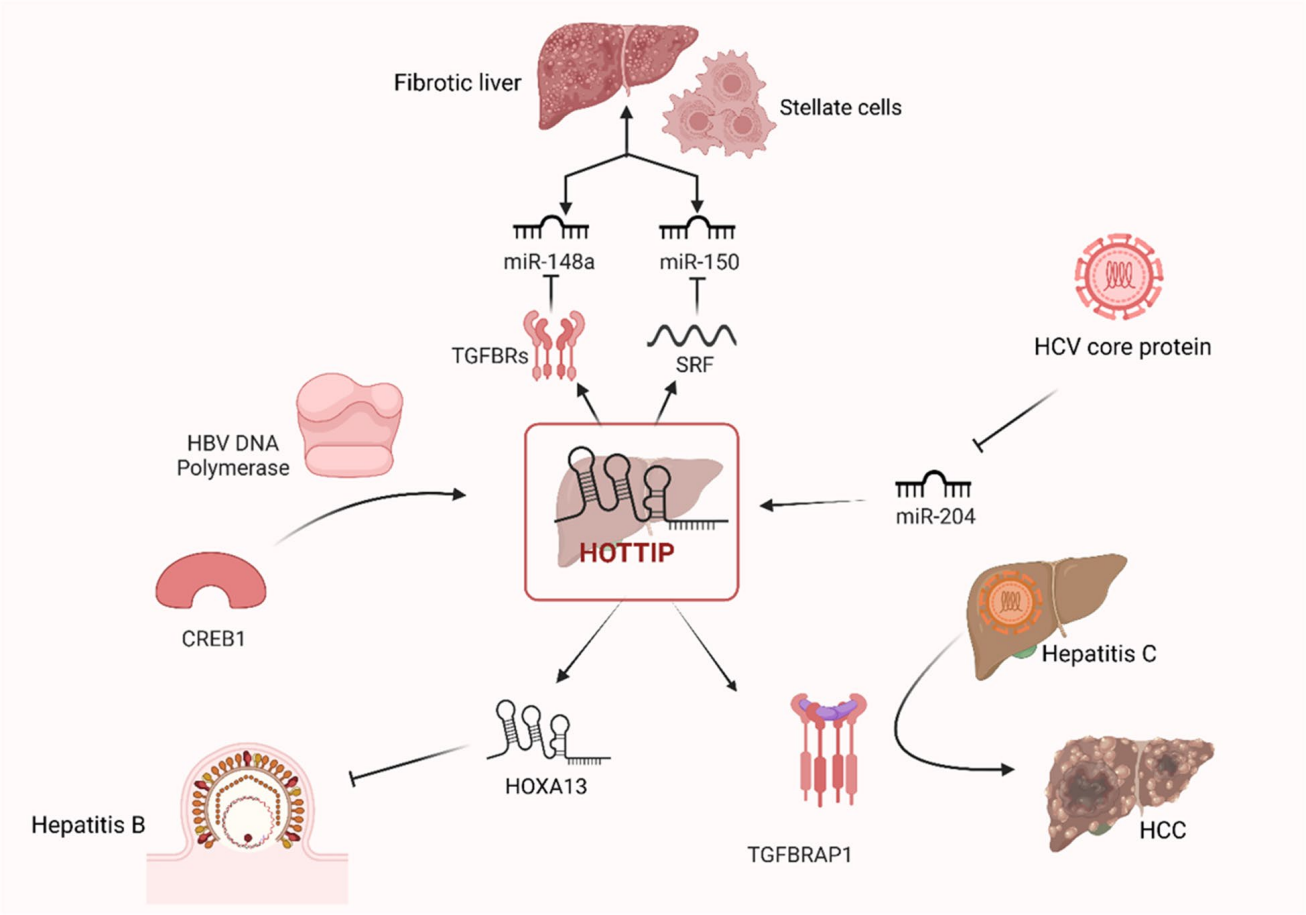
Mechanism of HOTTIP in different liver diseases [Created in BioRender. Hamdy, P. (2025) https://BioRender.com/n80y711]. HOTTIP, HOXA transcript at the distal tip; SRF, serum response factor; TGFBRs, tumor growth factor binding receptors; miR, microRNA; HCV, hepatitis C virus; TGFBRAP1, tumor growth factor binding receptor–associated protein 1; HCC, hepatocellular carcinoma; HBV, hepatitis B virus; CREB1, cAMP-responsive element-binding protein 1; HOXA13, homeobox A13

The mechanism by which HOTTIP participates in different liver diseases is summarized in Fig. 5 and Table 4.

**Table 4:** Mechanism of HOTTIP in different liver diseases

Liver disease	Target	Mechanism	Clinical implication	Ref
1) **Fibrosis**	SRF and TGFBRs	HOTTIP activates SRF and TGFBRs which in turn inhibits miR-150 and miR-148a that activates HSCs	Increased fibrosis	Zheng et al. 2019; Li et al. 2018b)
2) **Hepatitis B**	CREB1/HOTTIP/HOXA13 axis	HBV DNA polymerase activates CREB1/HOTTIP/HOXA13 axis	Inhibition of HBV replication	Zhao et al. 2021)
3) **Hepatitis C**	TGFBRAP1	HCV core protein inhibits miR-204 that increases HOTTP level then it upregulates TGFBRAP1	Enhancing the carcinogenic potential of HCV infection	Wang et al. 2018c)

*HOTTIP* HOXA transcript at the distal tip, *SRF* serum response factor, *TGFBRs* tumor growth factor binding receptors, *miR* microRNA, *HSCs* hepatic stellate cells, *HCV* hepatitis C virus, *TGFBRAP1* tumor growth factor binding receptor–associated protein 1, *HCC* hepatocellular carcinoma, *HBV* hepatitis B virus, CREB1 cAMP-responsive element-binding protein 1, *HOXA13* homeobox A13

The mechanism of lncRNA HOTTIP in other non-cancerous diseases is summarized in Table 5.

**Table 5:** Mechanism of HOTTIP in different non-cancerous diseases

Disease	Expression in disease vs. normal	Mechanism	Study population	Ref
**SSc**	Up	Increases fibrosis via acting as ceRNA for the anti-fibrotic miR‐150	Egyptian humans	Abd-Elmawla et al. 2020)
**Pre-eclampsia**	Down	Suppresses progression by inhibition of RND3	Chinese humans	Li et al. 2018a)
**Lung fibrosis**	Up	Enhances the fibrosis by ↓ levels of miR-744-5p and ↑ levels of PTBP1	Cell lines	Li et al. 2021)
		Promote cell injury by recruiting DNMT1 to inhibit SP-C expression	Cell lines	Li et al. 2024b)
**ARDS**	Up	↑ Progression by targeting miR-574-5p	Chinese humans	Shi et al. 2024)
**OSF**	Up	Enhances the fibrosis by ↓ production of IL-6 and TNF-α	Cell lines	Lee et al. 2021)
**Parkinson’s disease**	Up	↑ Neuronal damage and microglial activation by modulating miR-615-3p/FOXO1	Cell lines and mouse model	Lun et al. 2022)
**Vascular oculopathy**	Up	Activates p38/MAPK pathway, and promotes angiogenesis by interacting with WDR5 and MLL	Rat and mice models	Gandhi et al. 2024; Sun and Liu 2018; Li et al. 2022a; Yuan et al. 2021a)
**Male infertility**	Down	Affects spermatogenesis via sponging miR‐128‐3p	Chinese Humans	Su et al. 2019a)
**Asthma**	Up	Induces inflammation via promoting EFNA3 transcription	Mice model	Wu et al. 2022)
**Hirschsprung disease**	Down	Decreases cell migration and proliferation	Chinese humans and cell lines	Xie et al. 2015)
**ALS**	Up in some brain areas and down in others	Affects neuronal development via sponging miR-124 in late-stage ALS	Mouse model	Rey et al. 2021)

*SSc* systemic sclerosis, *ceRNA* competing endogenous RNA, *RND3* Rho family GTPase 3, *DNMT1* DNA methyltransferase 1, *SP-C* surfactant protein-C, *miR* microRNA, *ARDS* acute respiratory distress syndrome, *PTBP1* polypyrimidine tract binding protein 1, *OSF* oral submucous fibrosis, *IL-6* interleukin-6, *TNF-α* tumor necrosis factor-α, *FOXO1* forkhead box O1, *SRF* serum response factor, *HBV* hepatitis B virus, *DNA* deoxynucleic acid, *CREB1* cAMP-responsive element-binding protein 1, *MAPK* mitogen-activated protein kinase, *WDR5* WD repeat-containing protein 5, *MLL* mixed lineage leukemia, *EFNA3* Ephrin A3, *ALS* amyotrophic lateral sclerosis

### HOTTIP in cancers

It is interesting to note that a recent study also discovered a positive link between tumors and normal tissue in terms of HOTTIP and HOX gene expression (Deng et al. 2015; Lian et al. 2016a; Lian et al. 2016b; Chang et al. 2016; Cheng et al. 2015). To put it briefly, HOTTIP has the ability to inhibit tumor suppressor genes while simultaneously recruiting histone-modifying enzymes to activate HOX genes.

#### HOTTIP in hematologic cancers

The clonal hematopoietic stem cell cancer known as acute myeloid leukemia (AML) is typified by a buildup of immature progenitor cells with halted differentiation, which suppresses hematopoiesis (Döhner et al. 2015). HOTTIP showed different roles in AML. Luo et al. demonstrated that HOTTIP recruits CTCF/cohesion complex which in turn promotes oncogene transcription and leukemia development via HOTTIP-mediated R-loop creation (Luo et al. 2022). Moreover, another study showed that HOTTIP induces proliferation and progression by sponging miR-608 (Yang et al. 2018; Zhuang et al. 2019).

Singh et al. also stated that HOTTIP is upregulated in AML. This in turn remodels the chromatin architecture around miR-196b to promote transcription thus represses tumor suppressors and promotes leukemogenesis (Singh et al. 2022). Furthermore, HOTTIP plays a role in leukemia by regulating HOXA9 mRNA and protein levels. This, in turn, influences cell survival, self-renewal, and the cell cycle of progenitor cells (Aryal and Lu 2024). It also alters HOXA-driven domain and gene expression which in turn promotes hematopoietic stem cell self-renewal (Luo et al. 2019).

#### HOTTIP in gynecological cancers

Globally, breast cancer (BC) is the most prevalent type of cancer. Most of the cases are females. Numerous risk factors, such as genetic and inherited susceptibility, are linked to the incidence of breast cancer. Breast cancer is a heterogenous disease concerning its genetics (Hong and Xu 2022). Psathas et al. studied the role of lncRNA HOTTIP in BC. They found that HOTTIP remodels chromatin to increase invasion, aggressiveness, and metastasis (Psathas et al. 2023). A study on cell lines confirmed that HOTTIP binds to miR-148a-3p and inhibits mediation of Wnt1 which leads to inactivation of Wnt/β-catenin signaling pathway which in turn facilitates the stemness of breast cancer (Han et al. 2020). It also promotes migration, invasiveness, and epithelial–mesenchymal transition (EMT) via the Wnt-β-catenin signaling pathway as well (Han et al. 2019). Through regulation of HOXA genes, HOTTIP affects BC in many aspects. It regulates expression of HOXA genes thus enhances proliferation (Hussen et al. 2022). Furthermore, it promotes cell growth and migration and inhibits cell apoptosis via upregulation of HOXA11 (Sun et al. 2018b).

The most common cause of death for women with gynecological cancer diagnoses is *ovarian cancer* (*OC*) (Arora et al. 2023). In general, it ranks as the fifth most common cause of mortality for women. This disease has a poor prognosis because the majority of cases are detected at an advanced stage. Zhang and his colleagues found that HOTTIP enhances migration, invasion, and cell viability via upregulation of hypoxia-inducible factor 1-alpha (HIF-1α) and vascular endothelial growth factor (VEGF) besides its role as an anti-apoptotic (Zhang et al. 2022a). It also promotes proliferation, migration, and invasion by activating mitogen-activated protein kinase (MAPK) kinase (MEK)/extracellular signal–regulated kinase (ERK) pathway (Liu et al. 2020b) while promoting metastasis through regulation of the miR-615-3p/SWI/SNF-related matrix–associated actin dependent regulator of chromatin subfamily E member 1 (SMARCE1) pathway (Wu et al. 2020).

Moreover, HOTTIP upregulates AKT2 by negatively regulating miR-148a-3p which thus inhibits apoptosis signal-regulating kinase 1 (ASK1)/c-Jun N-terminal kinase (JNK) which in turn increases OC progression (Tan et al. 2021). It enhances cell proliferation and invasion via activation of Wnt/β-catenin pathway as well (Zou et al. 2019). Concerning its effect on immune escape, HOTTIP promotes interleukin-6 (IL-6), which thus upregulates programmed death-ligand 1 (PD-L1) in neutrophils and inhibits the activity of T cells and accelerates immune escape of OC cells (Shang et al. 2019).

In high-income nations, *endometrial cancer* (*EC*) is the most prevalent gynecological cancer, and its prevalence is increasing worldwide. The primary underlying reason for this trend is the rising prevalence of obesity, even if an ageing population and a decline in benign hysterectomies have also played a role (Crosbie et al. 2022). Despite its poor outcome, only a few studies were performed. A study on Chinese human samples and cell lines demonstrated that HOTTIP promotes EC development via activating PI3K/AKT pathway (Guan et al. 2018).

One of the most prevalent cancers in women is *mammary cancer* (*MC*). Western affluent countries have seen an upsurge in the incidence of breast cancer. China has seen a startling rise in the incidence of MC in recent years as a result of increasing living standards and life stresses (Nagata et al. 2014). A recent study showed that HOTTIP promotes MC cell proliferation via PI3K/AKT pathway while inhibiting apoptosis via upregulating CyclineD1 (Gao et al. 2018a).

One of the most prevalent malignancies in the world, prostate cancer (PCa) is responsible for a significant amount of all cancer-related fatalities (Sung et al. 2021). Furthermore, there are other environmental, lifestyle, viral, and dietary risk factors that could contribute to PCa etiology, albeit the evidence for this is often limited (Gandaglia et al. 2021). Interestingly, HOTTIP facilitates cancer cell metastasis via Twist family bHLH transcription factor 1 (TWIST1)-WDR5-HOTTIP axis that regulates HOXA9 chromatin (Malek et al. 2017). Additionally, it enhances proliferation and migration by sponging miR-216a-5p (Yang et al. 2018). Meanwhile, it aggravates cancer progression by regulating HOXA13 (Zhang et al. 2016).

#### HOTTIP in digestive cancers

According to GLOBOCAN, *nasopharyngeal carcinoma* (*NPC*) is endemic in southern China, southeast Asia, and north Africa, with age-standardized rates of 4–25 cases per 100,000 people in these areas (Bray et al. 2018). HOTTIP was found to promote proliferation, migration, and invasion of NPC by sponging miR-4301 (Shen et al. 2019). That in turn enlightens its role as an oncogene. Many studies were performed on *oral tongue squamous cancer cell* (*OTSCC*) lines or patients. An interesting one showed that HOTTIP upregulates cyclins B, D1, and E to enhance growth while upregulating Bcl-2 and downregulating Bcl-2-associated X-protein (Bax) enhancing its role as an anti-apoptotic (Mu et al. 2018). Another in silico study sheds light on its effect on regulation of HOXA genes involved in proliferation, migration, and invasion (Basavarajappa et al. 2022). This was confirmed on a study on human subjects (Zhang et al. 2015b). Moreover, it enhances cell proliferation and migration via downregulating both miR-206 (Li et al. 2021a) and high-mobility group AT-hook 2 (HMGA2)–mediated Wnt/β-catenin pathway (Xiong et al. 2020). One final study was conducted on Chinese human *tongue squamous cancer cell* (*TSCC*) patients found out that HOTTIP correlates positively with T stage, clinical stage, and distant metastasis (Zhang et al. 2015b).

The 5-year survival rate for *esophageal cancer* is less than 20%, and over 600,000 cases are diagnosed worldwide each year (Sung et al. 2021). Furthermore, little is known about the risk factors for this cancer, and it is unknown how significant genetic and environmental factors are to pathophysiology (Li et al. 2021b). A study performed on Chinese human samples and cell lines showed that HOTTIP activates ATP-Binding Cassette sub-family G member 2 (ABCG2) which in turn mediates chemotherapeutic resistance thus poorer patients’ outcomes (Li et al. 2021c). The third most common cause of cancer-related deaths worldwide and the fifth most common type of cancer overall is *gastric cancer* (*GC*) (Smyth et al. 2020). The disease is extremely diverse in both genetic and phenotypic aspects. GC is another GIT-related cancer. Upregulation of HOTTIP in GC leads to many consequences. One of which is hypermethylation of HOXA genes thus affecting GC progression and prognosis in a bad manner (Chang et al. 2016; Yang et al. 2017a). Meanwhile, it enhances GC proliferation. This may be attributed to affecting EMT markers (Ye et al. 2016) or via activation of HOXA13/HOTTIP/insulin growth factor-binding protein 3 (IGFBP-3) axis (Wang et al. 2016).

Another very common GIT cancer is *colorectal cancer* (*CRC*). Because CRC is a complex disease, a number of pathways, including microsatellite/chromosomal instability, genetic, and epigenetic changes (Abd El Fattah et al. 2022; El-Sheikh et al. 2023; Emam et al. 2022; Hamdy et al. 2024; Rizk et al. 2024); therefore, the colorectal tissues may be responsive to various genes abnormally leading to initiated tissue proliferation. HOTTIP is upregulated in CRC which in turn can affect the cells on their basic DNA level like facilitating DNA synthesis and enhancing proliferation (Liu et al. 2020c). Additionally, it enhances migration and invasion by upregulating vimentin and N-cadherin, and downregulating E-cadherin (Liu et al. 2020c). Another study showed its impact on cell growth where it downregulates p21 to induce tumor growth (Lian et al. 2016b). It can also enhance metastasis and invasion via Dickkopf WNT signaling pathway inhibitor 1 (DKK1) downregulation (Rui et al. 2019). Finally, a study by Liu et al. stated that HOTTIP enhances proliferation and migration and induces apoptosis by targeting serum/glucocorticoid regulated kinase 1 (SGK1) (Liu et al. 2018).

In the USA, *pancreatic cancer* (*PC*) is now the third most common cause of cancer-related mortality, surpassing breast cancer (Rahib et al. 2021). Major genetic variables that drive PC etiology and development have been essentially established by significant efforts. A complex microenvironment that coordinates metabolic changes and fosters a milieu of interactions among different cell types is a characteristic of pancreatic tumors (Halbrook et al. 2023). Many studies concerned with HOTITP and its correlation with PC. One study showed that HOTTIP promotes EMT and regulates pancreatic cancer stem cells (CSCs) via induction of HOXA19 and then activation of Wnt pathway (Li et al. 2015a). It also enhances proliferation, survival, and migration via WDR5/MLL1 chromatin–modifying complexes (Cheng et al. 2015). Moreover, HOTTIP binds to WDR5 and enhances the expression of HOXA9. This might further enhance stem cell properties by regulating the Wnt/β-catenin pathway (Fu et al. 2017).

Additionally, a study on cell lines demonstrated that HOTTIP upregulates HOXA13 and Aurora A kinase expression which may promote aneuploidy (Li et al. 2015a; Fu et al. 2017). An in silico study showed that HOTTIP promotes progression through sponging miR-497, the canonical HOTTIP-HOXA13 axis, and noncanonical trans-acting HOTTIP-WDR5-MLL1 axis (Wong et al. 2020). Ye and his colleagues stated that HOTTIP suppresses metabotropic glutamate receptor 1 (mGluR1) and lessened the activation of PI3K/Akt/mechanistic target of rapamycin (mTOR) pathway to reduce cell viability. Moreover, they demonstrated that HOTTIP induces apoptosis via promoting caspase-3 and caspase-8 activities and increasing Bax expression (Ye et al. 2018).

Another example of the most common tumors in the world is *hepatocellular carcinoma* (*HCC*). HCC typically arises in genetically predisposed people who are exposed to risk factors, particularly when liver cirrhosis is present (Toh et al. 2023). Despite the existence of hereditary disorders that enhance the risk of HCC, more studies are needed (Toh et al. 2023). Sponging and inhibiting mRNAs are very common mechanisms of HOTTIP in HCC. For example, it promotes cell viability by inhibition of miR-205 (Chen et al. 2022). Moreover, miR-192/−204-HOTTIP axis may interrupt HCC glutaminolysis through glutaminase 1 (GLS1) inhibition thus affecting HCC cell growth (Ge et al. 2015).

#### HOTTIP in lung-related cancers

With a projected 130,180 casualties, or up to 21% of all cancer-related deaths in the USA in 2022, lung cancer is the top cause of cancer mortality worldwide with *small cell lung cancer* (*SCLC*) accounting for about 14% of these cases (ACS 2022). Many studies conducted on SCLC stated that HOTTIP promotes proliferation and progression by regulation of miR-574-5p/enhancer of zeste homologous protein 1 (EZH1) (Sun et al. 2017). On the other hand, it regulates apoptosis by binding to miR-216a and upregulating BCL-2 (Sun et al. 2018a), or by suppressing HOXA13 (Sang et al. 2016). Additionally, it binds to miR-574-5p that in turn acts through vimentin (VIM), which is a key marker of EMT (Sun et al. 2021). The most common subtype of lung cancer is *non-small cell lung cancer* (*NSCLC*). However, a large number of patients who consent to surgery probably experience local recurrence or distant metastases (Alduais et al. 2023). This obligates researchers to study genetics and epigenetics of NSCLC. One study showed that lncRNA HOTTIP promotes hypoxia-induced glycolysis through targeting miR-615-3p/HMGB3 axis thus controls cell division and facilitates tumor cells’ avoidance of apoptosis (Shi et al. 2019).

Like in SCLC, HOTTIP promotes NSCLC proliferation, migration, and inhibits apoptosis by regulating HOXA13 (Sang et al. 2016). One subtype of NSCLC is *lung adenocarcinoma* (*LA*), a collection of lung cancers called after the types of cells they contain and their appearance under a microscope (Willner et al. 2024). In order to stage LA, clinicians employed invasive techniques. In difficult circumstances, this necessitates a more precise approach to cancer measures and enhances prognostic classification (Willner et al. 2024). One study stated that HOTTIP promotes proliferation by regulating AKT signaling pathway that boosts growth and survival in reaction to external cues (Zhang et al. 2017a). Furthermore, lncRNA HOTTIP contributes to tumor growth while it inhibits cell apoptosis by downregulation of pro-apoptotic factor Bad and upregulation of anti-apoptotic factor Bcl-2 (Deng et al. 2015).

#### HOTTIP in glioblastoma

Notwithstanding in-depth molecular investigations of *glioblastoma* (*GBM*) cells, it remains the most lethal kind of malignant brain tumor (Bikfalvi et al. 2023; Hamdy et al. 2023; Sokolov et al. 2024). The tumor microenvironment (TME) has gained attention recently as a key therapeutic target essential to the invasion and metastasis of tumors (Elanany et al. 2023) that may participant in GBM. However, in order to create more effective treatments, a comprehensive and integrated understanding of the various cellular and molecular components involved in the GBM’s TME and their interconnections is required (Bikfalvi et al. 2023). LncRNA HOTTIP shows a controversial role in GBM. A study by Xu et al. demonstrated that it is downregulated in GBM and this leads to downregulation of P53 and upregulation of CDK2 and cyclin A to induce cell growth (Xu et al. 2016). Another study showed high levels of HOTTIP induced by hypoxia. This hypoxia promotes EMT by regulating miR-101/zinc finger E-box binding homeobox 1 (ZEB1) axis (Zhang et al. 2017b).

#### HOTTIP in retinoblastoma

A pediatric tumor that develops in the developing retina is called *retinoblastoma* (*RB*). Between 1/16,000 and 1/18,000 live births are the global incidence (Bouchoucha et al. 2023). The understanding of epigenetics has greatly enhanced our understanding of the molecular biology of retinoblastoma and its somatic genetic changes. The upregulation of HOTTIP sponges miR-101-3p to upregulate stanniocalcin 1 (STC1), thereby promoting proliferation and inhibiting apoptosis in RB cells (Yuan et al. 2021a).

#### HOTTIP in papillary thyroid cancer

It is commonly known that the pathophysiology and prognosis of *papillary thyroid cancer* (*PTC*) are associated with genetic changes (Armos et al. 2024). Thyroid cancer was mainly attributed to ncRNA expression dysregulations that have been linked to the development of PTC in the past decades. LncRNA HOTTIP, which is upregulated in PTC, regulates proliferation and apoptosis by miR-744-5p regulation (Yuan et al. 2020) while promoting proliferation, invasion, and migration by regulating miR-637 (Yuan et al. 2018). This can also be achieved through activation of the MEK/ERK pathway (Esfandi et al. 2024).

#### HOTTIP in Head and neck squamous cell carcinoma

Among the head and neck cancers with the worst prognosis is *head and neck squamous cell carcinoma* (*HNSCC*), which is rather uncommon (Garneau et al. 2018). According to reports, the 5-year survival rate for patients with HNSCC is between 30 and 35% (Newman et al. 2015). Investigating novel and potent ncRNAs that may be viable targets is necessary in light of the difficulties presented by the current therapeutic approaches. It was found that HOTTIP/CTCF interaction promotes progression by targeting *HOXA9* gene (Sun et al. 2020).

#### HOTTIP in renal cell carcinoma

About 85% of kidney cancer cases are *renal cell carcinoma* (*RCC*), making it the most prevalent form of kidney cancer (Akgul and Williamson 2022). RCC patients frequently have metastasis and recurrence, which can have a negative clinical impact. However, one factor making successful therapy of RCC tumors more difficult is the absence of sensitive biomarkers for these tumors (Zhang and Zhu 2021). Mining for ncRNAs that could be useful for finding new targets became a must. A recent study found that lncRNA HOTTIP promotes progression through regulation of the miR-615/insulin‑like growth factor-2 (IGF-2) pathway (Wang et al. 2018d). Moreover, it promotes progression through regulation of miR-506 (Zhang et al. 2024a). Finally, a study performed on human samples, cell lines, and mouse models showed that HOTTIP affects RCC cell progression by regulating autophagy via the PI3K/Akt/autophagy-related 13 (Atg13) signaling pathway (Su et al. 2019b).

#### HOTTIP in osteosarcoma

In addition to pain, swelling, and fractures, patients with bone sarcomas experience extreme aggression (Rossi and Del Fattore 2023). Malignant bone tumors that are most common in teenagers and young adults are called *osteosarcoma* (*OS*) (Mann et al. 2022). The incidence of OS cures has not increased over the past 40 years, despite the rise in clinical trials. High dosages of non-specific targeted medicines result in side effects and poor therapeutic effects (Xu et al. 2022). Finding new genetic and epigenetic targets is boosted by this. Upregulation of HOTTIP enhances proliferation, migration, and invasion by regulating HOXA13 (Li et al. 2015b). It also interacts with polypyrimidine tract binding protein 1 (PTBP1) to promote KH-type splicing regulatory protein (KHSRP) level, and this leads finally to facilitation of proliferation, invasion, and migration (Yao et al. 2021b).

An important role of HOTTIP is highlighted by a recent study. HOTTIP/miR-27a-3p axis can regulate G protein subunit Gamma 12 (GNG12) expression which in turn affects progression and metastasis of OS. Furthermore, it promotes migration and invasion by upregulating cellular Myelocytomatosis (c-Myc) (Tang and Ji 2019). By controlling the expression of metabolic modulators, c-Myc functions as a transcriptional factor and contributes to several metabolism-related processes, including glutaminolysis, mitochondrial respiration, and glycolysis (Prieto et al. 2021; Tambay et al. 2021).

From the above section, we can conclude that HOTTIP’s common role in all cancers is that it frequently acts as an oncogene by regulating the transcription of HOXA genes, impacting cell proliferation, migration, invasion, and survival. It also activates signaling pathways such as Wnt/β-catenin, PI3K/AKT, and MAPK/ERK, which are pivotal in cancer progression. HOTTIP modifies chromatin architecture, often recruiting chromatin-modifying complexes (e.g., WDR5/MLL1) to enhance oncogene expression. Additionally, it sponges miRNAs (e.g., miR-608, miR-148a-3p) to derepress target oncogenes, facilitating tumor growth and metastasis.

Interestingly, EMT promotion is a recurring role of HOTTIP, enabling cancer cells to become more invasive and metastatic, as observed in breast, pancreatic, and lung cancers. Also, HOTTIP inhibits apoptosis by regulating Bcl-2 family proteins and suppressing pro-apoptotic miRNAs, ensuring cancer cell survival.

On the contrary, HOTTIP has divergent roles in different cancers. This includes acute myeloid leukemia (AML) where HOTTIP facilitates leukemogenesis through R-loop creation and HOXA9 regulation. While in ovarian cancer, it modulates immune escape by increasing IL-6 and PD-L1 expression, a mechanism unique to immune-regulatory pathways. Moreover, in glioblastoma, HOTTIP shows conflicting roles, being downregulated in some studies, which leads to reduced tumor growth through the suppression of cell cycle regulators like CDK2 and cyclin A. Finally, in esophageal cancer, HOTTIP mediates chemotherapeutic resistance by activating ABCG2, affecting treatment outcomes.

It has organ-specific functions as well. We conclude that in hepatocellular carcinoma (HCC), HOTTIP influences glutaminolysis and sponges miR-205 to promote cell viability. On the other hand, in renal cell carcinoma (RCC), it regulates autophagy via the PI3K/AKT/Atg13 pathway, a process less commonly reported in other cancers.

Therapeutic implications of HOTTIP include therapeutic targeting as there are many strategies to inhibit HOTTIP (e.g., antisense oligonucleotides or small molecule inhibitors). This may suppress tumor progression by disrupting oncogenic pathways and restoring normal apoptotic processes. Targeting HOTTIP’s interaction with miRNAs (e.g., miR-196b, miR-615-3p) or chromatin-modifying complexes could offer cancer-specific therapeutic benefits as well. In cancers like esophageal cancer, targeting HOTTIP-mediated pathways (e.g., ABCG2) could enhance chemotherapeutic efficacy. Future research should focus on developing precise tools to modulate HOTTIP activity, tailoring approaches to its context-specific roles in different cancers. Summary of studies which assessed expression and mechanism of HOTTIP in cell lines, animal models, and human samples can be seen in Table 6.

**Table 6:** Mechanism of HOTTIP in different cancers

Cancer type	Expression	Targets/regulators	Function	Study population	Ref
**AML**	Up	CTCF and R-loops	Recruits CTCF/cohesin complex and R-loop-associated regulators	US human, cell lines, and mouse model	Luo et al. 2022)
		miR-608/DDA1 axis	Induces proliferation and progression by sponging miR-608 and overstimulation of DDA1	Cell lines	Yang et al. 2018; Zhuang et al. 2019)
		miR-196b	Remodels the chromatin architecture around miR-196b to promote transcription thus represses tumor suppressors and promotes leukemogenesis	Cell lines and mouse model	Singh et al. 2022)
		HOXA9	Regulates HOXA9 mRNA and protein during leukemia thus regulates survival, self-renewal, progenitor cell cycle	Cell lines, mouse model, and in silico	Aryal and Lu 2024)
		Chromatin of HOXA genes	Alters HOXA-driven domain and gene expression thus promotes hematopoietic stem cell self-renewal	US human, cell lines, and mouse model	Luo et al. 2019)
**BC**	Up	Chromatin	Remodels chromatin to increase invasion, aggressiveness, and metastasis	Greek human	Psathas et al. 2023)
		miR-148a-3p and Wnt1	Binds to miR-148a-3p and inhibits mediation of Wnt1, leads to inactivation of Wnt/β-catenin signaling pathway	Cell lines	Han et al. 2020)
		Wnt-β-catenin	Promotes migration, invasiveness, and EMT via Wnt-β-catenin signaling pathway	Mouse model and cell lines	Han et al. 2019)
		HOXA genes	Regulate expression of HOXA genes thus enhances proliferation	Iraqi human and in silico	Hussen et al. 2022)
		HOXA11	Promotes cell growth, migration and inhibits cell apoptosis via HOXA11 upregulation	Cell lines and mouse model	Sun et al. 2018b)
**Esophageal cancer**	Up	ABCG2	Activates ABCG2 to mediate chemotherapeutic resistance	Chinese human and cell lines	Li et al. 2021c)
		EMT	Promotes metastasis via induction of EMT		Chen et al. 2016b)
**GC**	Up	HOXA genes	Hypermethylates HOXA genes thus affects progression and prognosis	Chinese human and cell lines	Yang et al. 2017a)
				Cell lines	Chang et al. 2016)
		EMT markers	Enhances proliferation maybe via affecting EMT markers	Chinese human and cell lines	Ye et al. 2016)
		IGFBP-3	Enhances proliferation via activation of HOXA13/HOTTIP/IGFBP-3 axis	Taiwanese human, mouse model, and cell lines	Wang et al. 2016)
**OTSCC**	Up	Cyclins B, D1, E, Bcl-2, and Bax	Upregulates cyclins B, D1, and E to enhance growth, upregulates Bcl-2, downregulates Bax	Cell lines	Mu et al. 2018)
		HOXA genes	Regulates HOXA genes involved in proliferation, migration, and invasion	In silico	Basavarajappa et al. 2022)
				Chinese human	Zhang et al. 2015b)
		miR-206	Enhances proliferation and migration by downregulating miR-206	Chinese human and cell lines	Li et al. 2021a)
		HMGA2 and Wnt/β-catenin	Enhances proliferation, migration, and invasion by regulation of HMGA2-mediated Wnt/β-catenin pathway	Chinese human, mouse model, and cell lines	Xiong et al. 2020)
**GBM**	Down	Cyclin A, CDK2, and P53	Downregulates P53 and upregulates CDK2 and cyclin A to induce cell growth	Chinese human, mouse model, and cell lines	Xu et al. 2016)
	Up (induced by hypoxia)	miR-101/ZEB1 axis	Promotes hypoxia-induced EMT by regulating miR-101/ZEB1 axis	Chinese human and cell lines	Zhang et al. 2017b)
**CRC**	Up	p21	Downregulates p21 to induce tumor growth	Chinese human, mouse model, and cell lines	Lian et al. 2016b)
		DNA synthesis, vimentin, N-cadherin, and E-cadherin	Promotes proliferation by facilitating DNA synthesis and enhances migration and invasion by upregulating vimentin and N-cadherin, and downregulating E-cadherin	Chinese human, mouse model, and cell lines	Liu et al. 2020c)
		DKK1	Enhances metastasis and invasion via DKK1 downregulation	Chinese human, mouse model, and cell lines	Rui et al. 2019)
		SGK1	Enhances proliferation and migration and induces apoptosis by targeting SGK1	Chinese human and cell lines	Liu et al. 2018)
**OC**	Up	HIF-1α and VEGF	Upregulates HIF-1α and VEGF to enhance migration, invasion, and cell viability + being an anti-apoptotic	Cell lines	Zhang et al. 2022a)
		MEK/ERK	Promotes proliferation, migration, and invasion by activating MEK/ERK		Liu et al. 2020b)
		miR-615-3p/SMARCE1 pathway	Promotes metastasis through regulation of the miR-615-3p/SMARCE1 pathway	Chinese human, mouse model, and cell lines	Wu et al. 2020)
		miR-148a-3p/AKT2 and ASK1/JNK	Upregulates AKT2 by negatively regulating miR-148a-3p inhibits ASK1/JNK increasing progression	Chinese human and cell lines	Tan et al. 2021)
		Wnt/β-catenin	Enhances cell proliferation and invasion via activation of Wnt/β-catenin	Chinese human and cell lines	Zou et al. 2019)
		IL-6 and PD-L1	Promotes IL-6, thus up-regulates PD-L1 in neutrophils and inhibits the activity of T cells and accelerates immune escape of OC cells	Chinese human, mouse model, and cell lines	Shang et al. 2019)
**PC**	Up	HOXA19/Wnt pathway	Promotes EMT and regulates pancreatic CSCs via induction of HOXA19, activation of Wnt	Cell lines	Li et al. 2015a)
		WDR5/MLL1 pathway	Enhances proliferation, survival, and migration via WDR5/MLL1 chromatin modifying complexes		Cheng et al. 2015)
		HOXA13 and Aurora A kinase	Upregulates HOXA13 and Aurora A kinase expression promote aneuploidy		Cheng et al. 2015; Li et al. 2015a)
		WDR5/HOXA9 and Wnt/β-catenin pathway	Binds to WDR5 and enhances the expression of HOXA9, enhances stem cell properties regulating the Wnt/β-catenin	Chinese human, mouse model, and cell lines	Fu et al. 2017)
		miR-497, HOXA13, and WDR5/MLL1 pathway	Promotes progression through sponging miR-497, the canonical HOTTIP-HOXA13 axis and noncanonical trans-acting HOTTIP-WDR5-MLL1	In silico	Wong et al. 2020)
		mGluR1 and PI3K/Akt/mTOR pathway caspase-3, caspase-8, and Bax	Suppresses mGluR1 and mitigated activation of PI3K/Akt/mTOR pathway to reduce cell viability Induce apoptosis via promoting caspase-3 and caspase-8 activities and increase Bax expression	Chinese human and cell lines	Ye et al. 2018)
**RB**	Up	miR-101-3p and STC1	Sponges miR-101-3p to upregulate STC1, thereby promoting proliferation and inhibiting apoptosis	Cell lines	Yuan et al. 2021a)
**HCC**	Up	miR-205	Promotes cell viability by inhibition of miR-205	In silico	Chen et al. 2022)
		miR-192/−204 and GLS1	miR-192/−204-HOTTIP axis interrupt HCC glutaminolysis through GLS1 inhibition	Chinese human, cell lines, and mouse model	Ge et al. 2015)
		hnRNPA2B1/DKK1/Wnt/β-catenin	Facilitates growth by stimulating hnRNPA2B1/DKK1/Wnt/β-catenin regulatory axis	Cell lines, mouse model, and in silico	Zeng et al. 2024)
		PPAR pathway	Promotes tumorigenesis and development through PPAR signaling	Chinese human and in silico	Zhang et al. 2017c)
**SCLC**	Up	miR-574-5p/EZH1, miR-216a/BCL-2, and HOXA13	Promotes proliferation and progression via regulation of miR-574-5p/EZH1 while regulating apoptosis by binding to miR-216a and upregulating BCL-2, or by suppressing HOXA13	Chinese human, cell lines, and mouse model	Sun et al. 2017; Sun et al. 2018a; Sang et al. 2016)
		miR-574-5p and VIM	Binds to miR-574-5p, that in turn acts through VIM, which is a key marker of EMT	Cell lines	Sun et al. 2021)
**PCa**	Up	TWIST1-WDR5 and HOXA9	TWIST1-WDR5-HOTTIP regulates HOXA9 chromatin to facilitate metastasis	American human, cell lines, and mouse model	Malek et al. 2017)
		miR-216a-5p	Promotes proliferation, migration sponging miR-216a-5p	Chinese human, cell lines, and in silico	Yang et al. 2018)
		Wnt/β-catenin	Regulates cell proliferation and cell cycle arrest via activation of Wnt/β-catenin pathway	Chinese human and cell lines	Jiang et al. 2019)
		HOXA13	Contributes to progression by regulating HOXA13	Cell lines and mouse model	Zhang et al. 2016)
**OS**	Up	HOXA13	Enhances proliferation, migration, and invasion by regulating HOXA13	Chinese human and cell lines	Li et al. 2015b)
		PTBP1 and KHSRP	Facilitates proliferation, invasion, and migration by interaction with PTBP1 to promote KHSRP level	Chinese human, cell lines, and mouse model	Yao et al. 2021b)
		miR-27a-3p and GNG12	HOTTIP/miR-27a-3p may regulate GNG12 expression which affect progression and metastasis	In silico	Yuan et al. 2021b)
		c-Myc	Promotes migration and invasion by upregulating c-Myc	Chinese human and cell lines	Tang and Ji 2019)
**HNSCC**	Up	HIF-1α, CTCF, and HOXA9	HIF-1α or HOTTIP/CTCF promotes progression by targeting HOXA9	Chinese human, cell lines, and in silico	Sun et al. 2020)
**EC**	Up	PI3K/AKT	Promotes EC development via activating PI3K/AKT pathway	Chinese human and cell lines	Guan et al. 2018)
**NPC**	Up	miR-4301	Promotes proliferation, migration, and invasion by sponging miR-4301	Chinese human and cell lines	Shen et al. 2019)
**MC**	Up	PI3K/AKT pathway and CyclineD1	Promotes cell proliferation via PI3K/AKT pathway while inhibiting apoptosis via upregulating CyclineD1	Chinese human and CELL lines	Gao et al. 2018a)
**RCC**	Up	miR-615-3p, and IGF-2	Promotes progression through regulation of the miR-615/IGF-2 pathway	Chinese human, cell lines, and in silico	Wang et al. 2018d)
		miR-506	Promotes progression through regulation of miR-506	Cell lines and in silico	Zhang et al. 2024a)
		PI3K/Akt/Atg13 signaling pathway	Affects progression by regulating autophagy via the PI3K/Akt/Atg13 signaling	Chinese human, cell lines, and mouse model	Su et al. 2019b)
**PTC**	Up	miR-744-5p	Regulate proliferation and apoptosis via miR-744-5p regulation	Chinese human and cell lines	Yuan et al. 2020)
		miR-637	Promotes proliferation, invasion, and migration by regulating miR-637	Chinese human, cell lines, mouse model, and in silico	Yuan et al. 2018)
		MEK/ERK	Promotes proliferation and invasiveness through activation of the MEK/ERK	Iranian human	Esfandi et al. 2024)
**LA**	Up	AKT pathway	Promotes proliferation by regulating AKT signaling	Chinese human, cell lines, and in silico	Zhang et al. 2017a)
		Bad and Bcl-2	Contributes to tumor growth and inhibits cell apoptosis by downregulation of pro-apoptotic factor Bad and upregulation of anti-apoptotic factor Bcl-2	Chinese human, cell lines, and model mouse	Deng et al. 2015)
**TSCC**	UP	-	Correlates with T stage, clinical stage, metastasis	Chinese human	Zhang et al. 2015b)
**NSCLC**	Up	miR-615-3p/HMGB3 axis	Promotes hypoxia-induced glycolysis through targeting miR-615-3p/HMGB3	Chinese human, cell lines, and in silico	Shi et al. 2019)
		HOXA13	Promotes proliferation, migration and inhibits apoptosis by regulating HOXA13	Chinese human and cell lines	Sang et al. 2016)

*AML*, acute myeloid leukemia; *CTCF*, CCCTC binding factor; *US*, United States; *miR*, micro-RNA; *DDA1*, DET1- and DDB1-associated protein 1; *BC*, breast cancer; *Wnt*, wingless-related integration site; *ABCG2*, ATP-binding cassette sub-family G member 2; *GC*, gastric cancer; *HOXA*, homeobox A; *IGFBP-3*, insulin growth factor-binding protein 3; *OTSCC*, oral tongue squamous carcinoma cells; *Bcl-2*, B cell lymphoma 2; *Bax*, BCL2-associated X; *HMGA2*, high-mobility group AT-hook 2; *GBM*, glioblastoma; *CDK2*, cyclin-dependent kinase 2; *ZEB1*, zinc finger E-box binding homeobox 1; *CRC*, colorectal cancer; *DKK1*, Dickkopf WNT signaling pathway inhibitor 1; *SGK1*, serum/glucocorticoid regulated kinase 1; *OC*, ovarian cancer; *HIF-1α*, hypoxia-inducible factor 1-alpha; *VEGF*, vascular endothelial growth factor; *MEK*, mitogen-activated protein kinase (MAPK) kinase; *ERK*, extracellular signal–regulated kinase; *SMARCE1*, SWI/SNF-related matrix-associated actin-dependent regulator of chromatin subfamily E member 1; *ASK1*, apoptosis signal-regulating kinase 1; *JNK*, c-Jun N-terminal kinase; *IL-6*, interleukin-6; *PD-L1*, programmed death-ligand 1; *PC*, pancreatic cancer; *EMT*, epithelial–mesenchymal transition; *CSCs*, cancer stem cells; *WDR5*, WD repeat–containing protein 5; *MLL1*, mixed lineage leukemia 1; *mGluR1*, metabotropic glutamate receptor 1; *mTOR*, mechanistic target of rapamycin; *RB*, retinoblastoma; *STC1*, Stanniocalcin 1; *HCC*, hepatocellular carcinoma; *GLS1*, glutaminase 1; *hnRNPA2B1*, heterogeneous nuclear ribonucleoprotein A2B1; *PPAR*, peroxisome proliferator-activated receptor; *SCLC*, small-cell lung cancer; *EZH1*, enhancer of zeste homologous protein 1; *VIM*, vimentin; *TWIST1*, twist family bHLH transcription factor 1; *PCa*, prostate cancer; OS, osteosarcoma; *KHSRP*, KH-type splicing regulatory protein; *PTBP1*, polypyrimidine tract binding protein 1; *GNG12*, G protein subunit gamma 12; *c-Myc*, cellular myelocytomatosis; *HNSCC*, head and neck squamous cell carcinoma; *EC*, endometrial cancer; *pI3K*, phosphoinositide 3-kinase; *AKT*, protein kinase B; *NPC*, nasopharyngeal cancer; *MC*, mammary cancer; *RCC*, renal cell carcinoma; *IGF‑2*, insulin‑like growth factor-2; *Atg13*, autophagy-related 13; *PTC*, papillary thyroid cancer; *LA*, lung adenocarcinoma; *BAD*, BCL2-associated agonist of cell death; *TSCC*, tongue squamous cell carcinoma; *NSCLC*, non-small-cell lung cancer; *HMGB1*, high mobility group box 1

## Comparative analysis of HOTTIP’s role in all diseases

To sum it up, we performed a comparative analysis to show the role of HOTTIP in different diseases, highlighting similarities and differences in their molecular function. We concluded that HOTTIP’s primary function involves regulating gene expression through chromatin remodeling, particularly within the HOXA gene cluster.

### Common roles across diseases

This includes the following:

- Epigenetic regulation: HOTTIP interacts with the WDR5-MLL complex, promoting histone H3K4 trimethylation and activating transcription of target genes within the HOXA cluster (Lian et al. 2016a). This mechanism is fundamental to its role in maintaining proper cellular function or driving pathology.
- Gene expression modulation: Across diseases, HOTTIP regulates genes involved in cell growth, differentiation, and survival. In cancers, this results in increased proliferation and resistance to apoptosis (Lin et al. 2017). In non-cancerous conditions, such as developmental disorders, it ensures spatial and temporal activation of genes critical for normal tissue patterning (Ka et al. 2022).
- Disease progression and severity: Dysregulation of HOTTIP, whether through overexpression or reduced activity, is associated with disease progression. High levels often correlate with more severe phenotypes, including advanced cancer stages, aggressive metastasis (Guo et al. 2020), and pronounced developmental abnormalities (Ka et al. 2022).

### Disease-specific roles:

This includes the following:

- In cancerous diseases such as hepatocellular carcinoma, pancreatic cancer, and colorectal cancer, HOTTIP facilitates tumor growth and metastasis by activating oncogenes and modulating signaling pathways like Wnt/β-catenin and PI3K/AKT (Lian et al. 2016a). Moreover, HOTTIP contributes to resistance against chemotherapy by upregulating drug efflux transporters or influencing cell cycle regulators. For instance, in small-cell lung cancer, HOTTIP promotes BCL-2 expression, inducing chemoresistance (Sun et al. 2018a). Regarding stemness and EMT, in certain cancers, HOTTIP enhances stem-like properties and EMT, supporting tumor initiation and invasion. In ovarian cancer, HOTTIP promotes proliferation and invasion, indicating its role in enhancing malignant properties (Liu et al. 2020b)

**Fig. 6:**
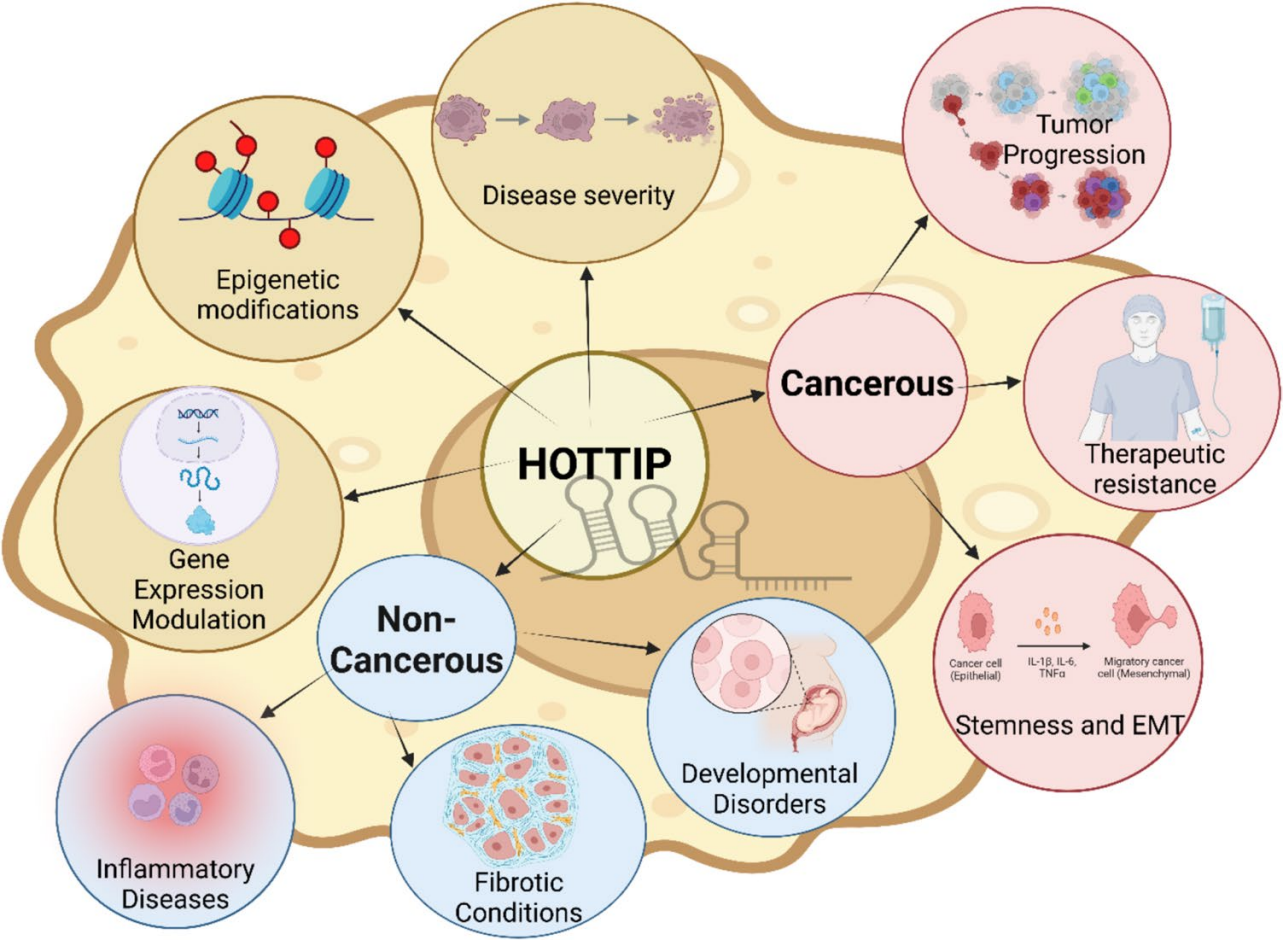
Comparative analysis of HOTTIP role in cancerous and non-cancerous diseases. [Created in BioRender. Elsisi, M. (2025) https://BioRender.com/j39f159]. The brown-colored circles represent similarities while blue circles represent different roles in non-cancerous diseases and the pink-colored circles represent the different roles in cancers. HOTTIIP; HOXA transcript at the distal tip, EMT; epithelial–mesenchymal transition

In non-cancerous diseases such as developmental disorders, HOTTIP is essential for limb development and spatial regulation of the HOXA genes. Misexpression during embryogenesis can result in congenital anomalies, such as limb malformations (Wang et al. 2011). For fibrotic conditions, preliminary studies suggest HOTTIP’s involvement in tissue remodeling and fibrosis by modulating genes responsible for extracellular matrix production (Li et al. 2021), though this area requires further investigation. Finally, regarding inflammatory diseases, dysregulation of HOTTIP in certain inflammatory or autoimmune disorders may influence the expression of immune-related genes, altering the immune response and contributing to disease pathogenesis (Zou and Xu 2020). This can be visualized in Fig. 6 Created in https://BioRender.com.

## HOTTIP and chemotherapeutic resistance

Targeted therapy, enhanced surgical techniques, targeted radiotherapy, and ongoing chemotherapy development have all contributed to significant advancements in cancer treatment over the years (Anand et al. 2023; Pomeroy et al. 2022). The discovery of methotrexate in the 1940 s marked the beginning of the discovery of cancer therapy, which has since produced over 100 chemotherapies and a wide range of targeted therapeutics (Dasari and Tchounwou 2014; Fornari et al. 2021). Through the recruitment of ribosomal subunits, lncRNAs can directly regulate protein translation and play the role of a microRNA sponge, according to cellular research of lncRNAs (Quinn and Chang 2016). It has been demonstrated that lncRNAs are both tumor suppressive and pro-oncogenic in the context of cancer research, with their capacity to control processes directly that are involved in the body’s reaction to medicinal substances. Since tissue-specific therapies might help reduce drug-induced side effects, lncRNA expression can be tissue-specific, making them an appealing target for therapeutic development (Ransohoff et al. 2018).

As per HOTTIP, it was found that overexpression of HOTTIP can reduce the sensitivity of sorafenib—a drug for HCC—in in vitro models (Quagliata et al. 2018). Experimentally, the naturally occurring alkaloid solamargine (SM) has demonstrated possible anticancer action by downregulating the long noncoding RNA HOTTIP and upregulating the expression of miR-4726-5p. This was linked to a reduction in HOTTIP's chemoresistant effect on HCC (Tang et al. 2022). The effects of systemic chemotherapy on cholangiocarcinoma (CCA), cancer in the bile ducts, are significantly hindered by chemo-resistance (Ilyas et al. 2018). A study that looked into the role of lncRNA HOTTIP in the chemo-resistance to cisplatin and gemcitabine in CCA revealed upregulated expression of HOTTIP in CCA patients, and this was associated with good medicinal response and prognosis (Gao et al. 2021). The study also found that HOTTIP silencing powerfully increased chemotherapy sensitivity by weakening proliferation and increasing apoptosis. Afterwards, miR-637 was identified as the functional target of HOTTIP because it could be targeted mechanically by HOTTIP, and functionally its overexpression dismissed the changes by HOTTIP silencing (Gao et al. 2021).

Although neoadjuvant therapy is a type of well-evidenced therapy for colorectal cancer (CRC), not all CRC patients respond well to it (Li et al. 2022b). When HOTTIP is silenced, CRC cells become much more chemosensitive, which increases cell apoptosis and the DNA damage response (DDR) to chemotherapeutic drug therapy (Liu et al. 2022a). Regarding prostate cancer (PCa), it was discovered that, in comparison to the controls, HOTTIP were higher in PCa patient samples and PCa cell lines (Jiang et al. 2019). In addition to preventing PCa cells from proliferating, HOTTIP knockdown promoted cell cycle arrest and increased chemosensitivity to cisplatin. Moreover, HOTTIP regulation in cell proliferation, cell cycle arrest, and chemoresistance to cisplatin in PCa was linked to Wnt/β-catenin signaling (Jiang et al. 2019).

In many tumors, including gastric cancer (GC), the development of multidrug resistance (MDR) has become a significant chemotherapy-related challenge (Vaidya et al. 2022). Tumor MDR is also aided by the epithelial-mesenchymal transition (EMT), which is thought to be a crucial step in the development of cancer. Salinomycin, an EMT inhibitor, lowers long noncoding RNA HOTTIP expression, which in turn lowers EMT-mediated multidrug resistance (Mao et al. 2019b). Worth noting that in GC cells resistant to cisplatin, the expression of HOTTIP was elevated, while its downregulation increased cisplatin sensitivity (Wang et al. 2019b). Furthermore, exosomes containing extracellular HOTTIP may transfer the resistance to cisplatin to susceptible cells. Furthermore, exosomal HOTTIP increased cisplatin resistance in GC cells by triggering high-mobility group A1 (HMGA1). It is interesting to note that miR-218 could directly attach to HOTTIP and that HMGA1 was one of its targets. On the clinical level, poor response to cisplatin treatment in GC patients was linked to increased expression of exosomal HOTTIP in serum (Wang et al. 2019b).

One of the deadliest cancers is pancreatic ductal adenocarcinoma (PDAC), which is mainly characterized by late diagnosis, a high risk of metastasis, and the chemoresistance (Siegel et al. 2022). An increasing body of research indicates that lncRNAs, such as HOTTIP, interact with critical signaling pathways to play a key role in the treatment resistance. Specifically, HOTTIP increases the ability of pancreatic cancer cells to withstand both cisplatin and gemcitabine by either upregulating HOXA13 or downregulating miR-137, a miRNA that initially makes cells more sensitive to cisplatin (Jiang et al. 2023). Other researchers claimed that this chemoresistance could be attributed to sponging of miR-137 and this can be applied to resistance against gemcitabine as well as cisplatin (Yin et al. 2020). Li and his associates investigated temozolomide (TMZ) resistance as a stand-in for better understanding the mechanism underlying therapeutic resistance in glioma cancer cells (Li et al. 2022a). They reported that HOTTIP was elevated in glioma cells that had both acquired and natural resistance to chemotherapy, with a critical mechanistic function for EMT and miR-10b. Therefore, more research is required to determine the significance of HOTTIP and miR-10b as targets for glioma therapy.

Turning then to lung adenocarcinoma, a significant histological form of lung cancer, the primary cause of therapy failure for lung adenocarcinoma is drug resistance (Xue et al. 2017). In lung adenocarcinomas, HOTTIP was overexpressed, and in the group that was not responsive to treatment, it was much elevated. Clinical samples produced comparable outcomes. This chemoresistance was linked, mechanistically, to HOTTIP’s interference with the AKT signaling pathway (Zhang et al. 2017a). Additionally, research was done on its function in small-cell lung cancer (SCLC). Through the regulation of B cell lymphoma 2 (BCL-2), mechanistic investigations demonstrated that HOTTIP boosted the expression of this anti-apoptotic factor and concurrently enhanced chemoresistance of SCLC (Sun et al. 2018a). As a result, HOTTIP’s demonstrated involvement in SCLC chemoresistance raises the possibility that it could be a novel diagnostic and predictive biomarker for SCLC therapeutic therapy.

One of the main forms of leukemia, chronic myeloid leukemia (CML), accounts for 15% of adult leukemias and has an annual incidence of between 1.6 and 2/100,000 worldwide (Deininger et al. 2020). In the bone marrow and cell lines of CML patients resistant to imatinib mesylate (IM), HOTTIP was abundantly expressed (Liu et al. 2022b). Knockdown of HOTTIP induced apoptosis and reduced the proliferation of CML cells in in vitro tests. Additionally, HOTTIP knockdown enhanced sensitivity to IM. Mechanistically, enhancer of zeste homologous protein 2 (EZH2) is recruited by highly expressed HOTTIP to suppress the expression of phosphatase and tensin homologous protein (PTEN) genes, hence contributing to the biological process of IM resistance (Liu et al. 2022b).

By using real-time polymerase chain reaction (RT-PCR) and following treatment with varying dosages of adriamycin (ADM) in cells, the serum level of HOTTIP in patients with esophageal cancer was determined in order to investigate its function in drug resistance (Li et al. 2021c). It was discovered that by favorably activating the ATP-binding cassette sub-family G member 2 (ABCG2) protein, HOTTIP controls medication resistance in esophageal cancer. On the other hand, resistance to cisplatin is a significant clinical issue in ovarian cancer, a lethal gynecological cancer (He et al. 2021b). Using paired cisplatin-sensitive and -resistant ovarian cancer cells, a study assessed the function of HOTTIP in the cisplatin resistance of these cells (Dong et al. 2021). It is interesting to note that HOTTIP was shown to sponge miR-205. While miR-205 increase in resistant cells was reported to resensitize cells to cisplatin, downregulation of miR-205 may lessen the silencing effects of HOTTIP. Zinc finger E-box binding homeobox 2 (ZEB2) was ultimately shown to be the miR-205 gene target, completing the analysis of HOTTIP-miR-205-ZEB2 as the novel axis that is functionally engaged in determining the resistance of ovarian cancer cells to cisplatin (Dong et al. 2021).

## HOTTIP SNPs

Gene expression is influenced by non-coding RNA polymorphisms, which can impact many diseases’ risk and prognosis (Taucher et al. 2016). Here, we report jointly the relationships between several diseases and the single nucleotide polymorphisms (SNPs) in the long non-coding RNA HOTTIP.

### HOTTIP SNPs in non-cancerous diseases

The recurring loss of two or more clinically diagnosed pregnancies within 24 weeks of gestation is referred to as recurrent spontaneous miscarriage. In about half of these cases, no known cause has been found; thus, they are called *idiopathic recurrent spontaneous abortion or IRSA* (Sherpa et al. 2024). It was found that pregnant females carrying TT genotype of HOTTIP rs2023843 have lower risk for IRSA while on the other hand, those with TT genotype of rs78248039 or carrying A-allele for rs1859168 have higher risk for IRSA. This was stated by a study conducted on Iranian females (Mirinejad et al. 2024).

*Adenomatous polyposis* (*AP*) is an autosomal dominant disease characterized by widespread colorectal polyposis and affected patients are very susceptible to develop CRC (Kyriakidis et al. 2023). Patients with AC genotype of HOTTIP rs1859168A/C showed higher risk for CRC development than any other genotype as demonstrated by Ali et al., in their study performed on Egyptian patients (Ali et al. 2020).

Usually, *knee osteoarthritis* (*KOA*) manifests as joint discomfort that becomes worse with activity and goes away with rest. The pathologic and radiological process of knee OA is not fundamentally reversed by pharmacologic or nonpharmacologic therapies, despite the fact that they are typically successful in reducing pain and enhancing physical function (Gelber and Knee 2024). In order to determine who is more likely to get KOA and who is not, this intensifies a thorough genetic search. C allele of HOTTIP’s rs2023843 was more distinct for younger age (age < 60) in KOA (Wang et al. 2018e).

*Hirschsprung’s disease* (*HSCR*) is a congenital condition that affects various intestinal lengths and results in a distal functional obstruction. It is characterized by the neural crest cells’ inability to migrate and populate the distal colon during gestation (Gershon et al. 2023). More studies are needed to elucidate the effect of SNPs on risk and susceptibility of HSCR. A Chinese study showed that there is no statistical evidence of a correlation between HOTTIP rs3807598 and susceptibility of HSCR (Zheng et al. 2020).

### HOTTIP SNPs in cancerous diseases

Pancreatic cancer is a very heterogenous disease concerning its genetics and epigenetics (Halbrook et al. 2023). Recent genetic study on Chinese human samples affected with *PC* stated that CC genotype vs AA and C allele lowered the risk of PC by decreasing HOTTIP expression (Hu et al. 2017). Many polymorphism-based studies are conducted on *HCC* patients. One of them found that GC genotype of HOTTIP rs2071265G/C increased the recurrence in HCC by increasing HOTTIP expression. Meanwhile, G alleles HOTTIP rs17501292G/T, C alleles of HOTTIP rs2067087G/C, and A alleles of HOTTIP rs17427960C/A increase HCC risk as well (Wu et al. 2018). Another interesting study showed that GG genotype of HOTTIP rs3807598G/C increased the survival time in HBV-negative HCC patients (Wang et al. 2018a).

Interestingly, a study on Egyptian females affected with *BC* demonstrated that CC genotype, C allele, and recessive model of HOTTIP rs1859168A/C increased the incidence of BC, and additionally, CC genotype increased HOTTIP expression and decreased miR-615-3p level as well (Abdelaleem et al. 2021). Concerning *NPC*, CC genotype and C allele of HOTTIP rs1859168A/C increased the incidence of NPC while CC genotype also increased NPC’s tumor invasion and lymph node metastasis. Finally, CC and CA genotypes of HOTTIP rs1859168A/C showed higher HOTTIP levels than other genotypes (Lao et al. 2024).

Another GIT-related cancer is gastric cancer. Many researchers studied SNPs in HOTTIP in patients with *GC*. One recent study figured out that CC genotype of HOTTIP rs2067087G/C and CG genotype of HOTTIP rs3807598G/C increased the susceptibility of individuals to develop GC (Wang et al. 2020). One more Iranian study stated that SNP-SNP interaction of CC genotype of HOTTIP rs1859168 with either TG genotype of HOTAIR rs17720428 or CC genotype HOTAIR rs7958904 increased the risk of getting GC (Abdi et al. 2020). Also, HOTTIP SNPs in *CRC* patients were studied. CC genotype of rs3807598C/G, GG genotype of rs2067087G/C, and CA genotype of rs17427960C/A are all associated with CRC risk; meanwhile, CG genotype of rs3807598C/G is associated with poor patient prognosis as well (Lv et al. 2019).

As for *lung cancer*, one study on Chinese patients figured out that C allele of HOTTIP rs5883064 or A allele of HOTTIP rs1859168 increased lung cancer risk (Gong et al. 2016). Finally, a study conducted on Chinese Han females affected with cervical cancer (CC) showed that A allele of HOTTIP rs1859168A/C decreased the risk of CC when compared to C allele (Dai et al. 2023). Summary of different SNPs and effect on different diseases are shown in Table 7. HOTTIP single nucleotide polymorphisms hold significant promise in personalized medicine by serving as biomarkers for cancer susceptibility, progression, and treatment response. Specific SNPs in the HOTTIP locus may influence their expression levels or functional interactions, thereby affecting key oncogenic pathways such as Wnt/β-catenin and PI3K/AKT. Identifying these genetic variations in individuals could help predict their risk of developing certain cancers, stratify patients based on disease aggressiveness, and tailor therapeutic strategies. For instance, patients with SNPs linked to heightened HOTTIP activity might benefit from targeted therapies aimed at disrupting HOTTIP-mediated pathways, improving treatment outcomes while minimizing side effects.

**Table 7:** HOTTIP single nucleotide polymorphism in different diseases

Disease	Studied SNPs	Effect of studied SNPs	Study population/samples	Ref
**IRSA**	rs2023843C/T, rs78248039A/T, rs1859168C/A	TT genotype of rs2023843 ↓ risk for IRSA, the TT genotype of rs78248039, and A-allele for rs1859168 ↑ risk for IRSA	Iranian human samples	Mirinejad et al. 2024)
**GC**	rs2067087G/C and rs3807598G/C	CC genotype of rs2067087 and CG genotype of rs3807598 ↑ susceptibility to GC	Chinese human samples	Wang et al. 2020)
	rs1859168C/A	SNP-SNP interaction of CC genotype of HOTTIP rs1859168 with TG genotype of HOTAIR rs17720428 or CC genotype HOTAIR rs7958904 ↑ risk of GC	Iranian human samples	Abdi et al. 2020)
**AP**	rs1859168A/C	AC genotype ↑ risk for CRC development	Egyptian human	Ali et al. 2020)
**BC**	rs1859168A/C	CC genotype, C allele, and recessive model ↑ incidence of BC, CC genotype ↑HOTTIP expression, and ↓miR-615-3p level	Egyptian human	Abdelaleem et al. 2021)
**PC**	rs1859168A/C	CC vs AA, recessive and additive models and C allele ↓ risk of PC by ↓ HOTTIP expression	Chinese human	Hu et al. 2017)
**HCC**	rs2071265G/C	GC genotype ↑ recurrence in HCC by ↑ HOTTIP expression	Chinese human and cell lines	Wu et al. 2018)
	rs17501292G/T, rs2067087G/C, and rs17427960C/A	G, C, and A alleles ↑ HCC risk, respectively	Chinese human	Wang et al. 2018a)
	rs3807598G/C	GG genotype ↑ survival time in HBV negative HCC		
**HSCR**	rs3807598C/G	No statistical evidence of a correlation between rs3807598 and susceptibility of HSCR	Chinese human	Zheng et al. 2020)
**NPC**	rs1859168A/C	CC genotype and C allele ↑ NPC incidence while CC genotype ↑tumor invasion, and LN metastasis CC or CA genotypes had ↑ HOTTIP levels	Chinese human	Lao et al. 2024)
**CRC**	rs3807598C/G, rs2067087G/C, rs17427960C/A	CC genotype of rs3807598, GG genotype of rs2067087, and CA genotype of rs17427960 ↑ CRC risk CG genotype of rs3807598 = poor prognosis	Chinese human	Lv et al. 2019)
**Lung cancer**	rs5883064C/T and rs1859168A/C	rs5883064 C allele or rs1859168 A allele ↑ lung cancer risk	Chinese human	Gong et al. 2016)
**KOA**	rs2023843C/T	C allele of rs2023843 was more distinct for younger age (age < 60) in KOA	Chinese human	Wang et al. 2018e)
**CC**	rs1859168A/C	The A allele ↓ risk of CC	Chinese human	Dai et al. 2023)

*IRSA*, idiopathic recurrent spontaneous abortion; *GC*, gastric cancer; *HOTAIR*, HOX transcript antisense RNA; *AP*, adenomatous polyposis; *CRC*, colorectal cancer; *HOTTIP*, HOXA transcript at the distal tip; *PC*, pancreatic cancer; *HCC*, hepatocellular carcinoma; *HBV*, hepatitis B virus; *HSCR*, Hirschsprung disease; *NPC*, nasopharyngeal carcinoma; *LN*, lymph nodes; *KOA*, knee osteoarthritis; *CC*, cervical cancer

## HOTTIP and diagnosis/prognosis

The widely accepted understanding is that lncRNAs regulate both transcriptional and post-transcriptional aspects of epigenetic and gene expression (Qian et al. 2019). Moreover, the maintenance and differentiation of stem cells, cell autophagy and apoptosis, and embryonic development have all been linked to lncRNAs. Many cancers and other diseases have altered expression of particular lncRNAs, which suggests that lncRNAs have a role in carcinogenesis and/or tumor cell activity and can be used as predictors for diagnosis and prognosis in a variety of disorders (Kong et al. 2021; Shen et al. 2020).

HOTTIP expression has shown significant correlations with early diagnosis, disease progression, and prediction of outcomes in various cancers and some non-cancer conditions, for instance, the following:

1. *Hepatocellular carcinoma* (*HCC*): Elevated HOTTIP expression in HCC correlates with advanced tumor stage, increased metastasis, and poor overall survival. It has been proposed as a diagnostic biomarker for early detection and as a prognostic marker for patient outcomes.
2. *Pancreatic cancer*: High HOTTIP levels are associated with aggressive tumor characteristics, including increased invasion and metastasis. Patients with elevated HOTTIP expression often experience shorter survival times, underscoring its prognostic potential.
3. *Esophageal cancer*: HOTTIP upregulation has been linked to chemoresistance and disease progression. Its expression levels can help predict therapeutic outcomes and guide treatment strategies.
4. *Ovarian cancer*: HOTTIP expression enhances immune evasion by increasing IL-6 and PD-L1, indicating its potential as a biomarker for predicting immune checkpoint inhibitor responsiveness and overall prognosis.
5. *Acute myeloid leukemia* (*AML*): HOTTIP expression is elevated in AML and correlates with leukemogenesis through the regulation of HOXA genes, making it a potential biomarker for disease stratification and progression.
6. *Non-Cancer Diseases*: In fibrotic diseases, such as idiopathic pulmonary fibrosis, HOTTIP has been implicated in promoting fibroblast activation and extracellular matrix deposition, suggesting its utility as a marker for disease severity and progression.

These examples highlight HOTTIP’s role as a versatile biomarker for early diagnosis, monitoring disease progression, and predicting outcomes, though further clinical validation is essential to standardize its use in personalized medicine. Diagnostic/prognostic roles of HOTTIP in cancer and non-cancerous diseases can be seen in Table 8.

**Table 8:** Diagnostic/prognostic roles of HOTTIP in cancer and non- cancerous diseases

Disease	Role	Study population	Ref
**PC**	Diagnostic/prognostic	Human samples	Jiang et al. 2023; Wang et al. 2015)
	Diagnostic	Cell lines and mouse model	Lou et al. 2020)
	Prognostic	Human samples	Wang et al. 2018f; Lee et al. 2019)
**ARDS**	Diagnostic	Human samples	Shi et al. 2024)
**GC**	Diagnostic	Human samples	Ye et al. 2016; Zhao et al. 2018)
	Prognostic	In silico	Li et al. 2022c; Gao et al. 2018b)
	Prognostic	In silico and human samples	Zhang et al. 2019a)
**CRC**	Diagnostic	Human samples	Gharib et al. 2019; Ali Akbar-Esfahani et al. 2019; Tabaeian et al. 2024)
	Prognostic		Ren et al. 2015; Oehme et al. 2019)
**SSc**	Diagnostic	Human samples	Abd-Elmawla et al. 2020)
**AGA**	Diagnostic	Human samples	Shao et al. 2023)
**OC**	Prognostic	Human samples	Zou et al. 2019)
**HCC**	Prognostic	Human samples	Quagliata et al. 2014; Wu et al. 2018)
	Diagnostic	In silico and human samples	Kim et al. 2021)
		Human samples	Bao et al. 2024)
**Osteosarcoma**	Prognostic	Human samples	Li et al. 2015b; Li et al. 2017)
**TSCC**	Prognostic	Human samples	Zhang et al. 2015b)
**NSCLC**	Diagnostic	Cell lines	Sang et al. 2016)
	Prognostic	In silico and human samples	Navarro et al. 2019)
		In silico	Yang et al. 2024)
**SCLC**	Diagnostic/prognostic	Cell lines and mouse model	Sun et al. 2018a)
	Prognostic	Human samples and cell lines	Sun et al. 2017)
**OSCC**	Prognostic	Human samples and cell lines	Huang et al. 2019)
	Prognostic	In silico	Padam et al. 2022)
**HBV**	Prognostic	Human samples and cell lines	Yılmaz Susluer et al. 2018)
**CCA**	Prognostic	Human samples	Gao et al. 2021)
**HNSCC**	Prognostic	In silico, cell lines, and human samples	Yin et al. 2019; Zhang et al. 2020)
		In silico	Pan et al. 2019)
**RCC**	Prognostic	In silico	Liu et al. 2020d; Zhang et al. 2022b; Lu et al. 2022; Zhang et al. 2019b)
	Diagnostic	Cell lines and human samples	Peng et al. 2018)
		In silico	Zhang et al. 2024a)
		In silico, cell lines, and human samples	Wang et al. 2018d)
**BC**	Prognostic	In silico	Yang et al. 2017b)
**MC**	Prognostic	Human samples	Gao et al. 2018a)
**Glioma**	Prognostic	Cell lines	Zhang et al. 2017b)
**PRTs**	Prognostic	Human samples	Balcin et al. 2018)

*PC*, pancreatic cancer; *ARDS*, acute respiratory distress syndrome; *GC*, gastric cancer; *CRC*, colorectal cancer; *SSc*, systemic sclerosis; *AGA*, acute gouty arthritis; *OC*, ovarian cancer; *HCC*, hepatocellular carcinoma; *TSCC*, tongue squamous cell carcinoma; *NSCLC*, non-small cell lung cancer; *SCLC*, small-cell lung cancer; *OSCC*, oral squamous cell carcinoma; *HBV*, hepatitis B virus; *CCA*, cholangiocarcinoma; *HNSCC*, head and neck squamous cell carcinoma; *RCC*, renal cell carcinoma; *BC*, breast cancer; *MC*, mammary cancer; *PRTs*, pineal region tumors

**Fig. 7:**
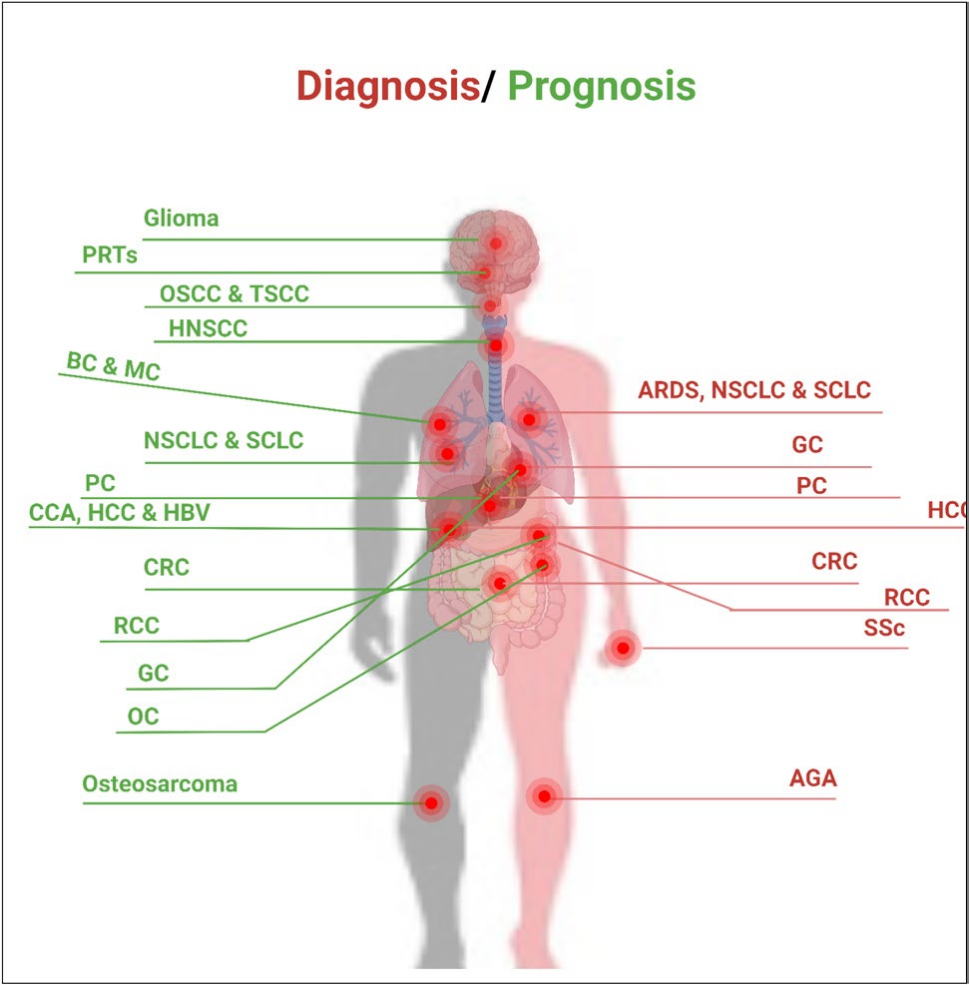
HOTTIP in diagnosis and prognosis in different cancerous and non-cancerous diseases. [Created in BioRender. Elsisi, M. (2025) https://BioRender.com/t72x537]. PC, pancreatic cancer; ARDS, acute respiratory distress syndrome; GC, gastric cancer; CRC, colorectal cancer; SSc, systemic sclerosis; AGA, acute gouty arthritis; OC, ovarian cancer; HCC, hepatocellular carcinoma; TSCC, tongue squamous cell carcinoma; NSCLC, non-small-cell lung cancer; SCLC, small-cell lung cancer; OSCC, oral squamous cell carcinoma; HBV, hepatitis B virus; CCA, cholangiocarcinoma; HNSCC, head and neck squamous cell carcinoma; RCC, renal cell carcinoma; BC, breast cancer; PRTs, pineal region tumors

This can be summarized in Fig. 7 created in BioRender.com.

## Multi-omics and HOTTIP

Multi-omics approaches offer a comprehensive strategy to study HOTTIP, uncover its molecular mechanisms, and translate findings into precision medicine (Mohr et al. 2024; Olivier et al. 2019). Specific examples include the following:

- Transcriptomics: RNA sequencing can quantify HOTTIP expression across different cancers and correlate its levels with clinical features such as tumor stage, metastasis, and response to therapy. For example, transcriptomic profiling in HCC has revealed upregulated HOTTIP as a marker of poor prognosis (Wang et al. 2018a).
- Epigenomics: ChIP-seq can identify HOTTIP’s role in recruiting chromatin-modifying complexes, such as WDR5/MLL1, to activate oncogenic HOXA genes (Ka et al. 2022). This approach elucidates epigenetic landscapes influenced by HOTTIP, aiding the development of targeted epigenetic therapies.
- Proteomics: Mass spectrometry-based proteomics can identify HOTTIP-interacting proteins and downstream effectors in signaling pathways like Wnt/β-catenin or PI3K/AKT (Hussain et al. 2024). For instance, this can reveal novel therapeutic targets to disrupt HOTTIP-mediated oncogenic pathways.
- miRNA Interactomics: Combining transcriptomics with miRNA profiling can pinpoint miRNAs sponged by HOTTIP (e.g., miR-608 or miR-148a-3p) (Li et al. 2014; Singh et al. 2022; Ge et al. 2015). These interactions can guide miRNA-based therapies to counteract HOTTIP’s oncogenic effects.
- Metabolomics: Metabolic profiling can uncover how HOTTIP influences metabolic reprogramming in cancer, such as its role in glutaminolysis in HCC (Ge et al. 2015). This insight can inform metabolic interventions tailored to tumors with high HOTTIP expression.
- Integrated Multi-Omics: Integration of genomics, transcriptomics, and proteomics can uncover genetic variants (e.g., HOTTIP SNPs) associated with its dysregulation and link them to specific phenotypes. For example, such integrative approaches can clarify HOTTIP’s dual role as an oncogene or tumor suppressor in a context-dependent manner.

These multi-omics strategies can refine HOTTIP’s role as a biomarker and therapeutic target, paving the way for personalized medicine tailored to its cancer- and context-specific functions.

## Summary and conclusions

**Fig. 8:**
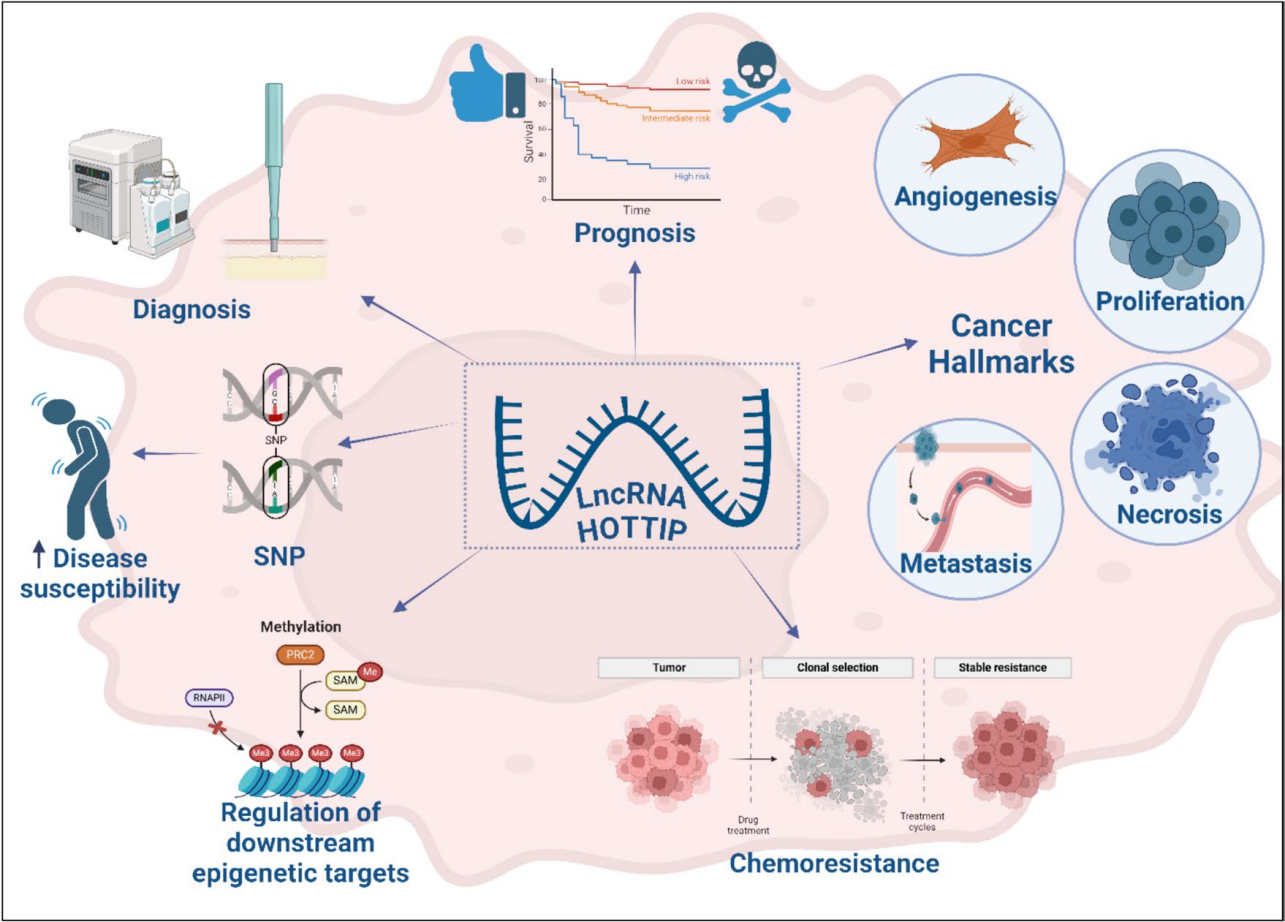
Graphical abstract of HOTTIP different roles in cancer. [Created in BioRender. Elsisi, M. (2025) https://BioRender.com/b66v480]. HOTTIP, HOXA transcript at the distal tip; SNP, single nucleotide polymorphism

In summary, the findings of this review highlighted the critical importance of HOTTIP is highlighted in Fig. 8 (Graphical Abstract, created in BioRender.com). Initially dismissed as mere “junk” or functionless DNA, HOTTIP has been revealed to play critical roles in biological systems. Through extensive database exploration and advanced in silico analysis, we demonstrated that HOTTIP exhibits diverse expression patterns, contributing to its regulatory roles in both cancerous and non-cancerous diseases. Notably, for the first time, we explored its role in normal cells, uncovering its influence on disease pathophysiology, cancer hallmarks, and overall survival. Furthermore, we underscored the potential of this lncRNA as a diagnostic and prognostic biomarker for various conditions. Lastly, we identified how single-nucleotide polymorphisms (SNPs) in HOTTIP significantly impact disease outcomes.

### Future prospective and recommendations

As we look ahead, the findings of this review underscore the importance of lncRNAs, specifically HOTTIP. The rapid evolution of research and disease complications obligate us to be nimbler to discover new insights concerning ncRNAs. We recommend more studies, especially population-based ones, on larger sample sizes in different cancerous and non-cancerous diseases to be carried out by other researchers to fill that research gap. Furthermore, subsequent investigations are required to study HOTTIP SNPs across various cancer types which in turn will facilitate the early detection of diseases, particularly at initial stages or lower grades and thus improving patient outcomes. Additionally, for the application of precision medicine, we recommend integration of multi-omics data as future research should focus on the integration of genomic, proteomic, metabolomic, and other omics data to create comprehensive patient profiles. This holistic approach can enhance the predictive accuracy of disease susceptibility and treatment responses, facilitating more personalized therapy options. Moreover, HOTTIP-based cancer vaccine design would be a milestone via reversed vaccinology approach (Bhattacharya et al. 2022).

Further studies are required to explore the relation of HOTTIP with SNPs (Mesallamy et al. 2014), adipokines’ SNPs (Aboouf et al. 2015) or adipokines expression (Mostafa et al. 2015; Zhang et al. 2024b), and inflammation molecular markers that are called the tumor immune microenvironment (TIME) (Anwar et al. 2022) as well as the possibility of studying vitamins that modulate the TME like vitamin E (Hamdy et al. 2009) or vitamin D (Chen et al. 2023), insulin resistance or insulin like growth factors (El-Mesallamy et al. 2013a; El-Mesallamy et al. 2013b; Hamdy 2011; LeRoith et al. 2021), and more-metabolism-related hormones or genes in various non-communicable diseases (Aboouf et al. 2015; Eissa et al. 2003; Hammad et al. 2022; Khalil et al. 2016; Sanad et al. 2017). It is worth noting that as a research team, we are currently working on a study stating HOTTIP SNPs’ roles in human cancer samples and other study on its genetic expression.

### Limitations

1. *Narrow selection of studies*: The review may be constrained by the inclusion of studies published in specific languages or indexed in certain databases, potentially excluding unpublished or less accessible research.
2. *Small sample sizes in primary studies*: Many of the studies on lncRNA HOTTIP are based on small cohorts or limited experimental models, which restrict the generalizability and robustness of the conclusions drawn.
3. *Incomplete understanding of mechanisms*: Although HOTTIP has been implicated in key processes such as epigenetic regulation and cancer progression, the full spectrum of its biological roles and molecular mechanisms remains poorly understood, leaving gaps in the narrative.
4. *Lack of clinical trials/population-based studies on HOTTIP*: Despite its potential as a diagnostic or therapeutic target, there are few, if any, clinical trials directly investigating HOTTIP. Moreover, most existing studies are case–control in design and cross-sectional, which limits causality inference. Additionally, sample sizes are modest, usually between 50 and 200 patients and there is a lack of longitudinal studies or multi-center cohorts that could more robustly establish HOTTIP’s role as a biomarker or effector molecule.
5. *Redundancy with other lncRNAs*: Functional overlap between HOTTIP and other lncRNAs, particularly those involved in the regulation of HOX genes, may complicate the identification and interpretation of its unique biological roles and contributions.
6. *Challenges in clinical translation*: Despite growing evidence linking HOTTIP to disease pathogenesis, the translation of these findings into clinical applications, such as diagnostics or targeted therapies, remains in its infancy. The clinical translation of HOTTIP-based therapies faces several barriers, including limited understanding of its precise molecular mechanisms across diverse cancer types, the potential for off-target effects due to its widespread role in gene regulation, and challenges in delivering RNA-targeting therapies effectively in vivo. Additionally, the heterogeneity of HOTTIP expression and function in different cancers complicates the development of universal therapeutic approaches. To overcome these challenges, further research is needed to elucidate HOTTIP’s tissue-specific roles and interactions.
7. *Lack of standardized functional assays*: The diverse methodologies used to investigate HOTTIP’s expression, localization, and functional roles introduce variability in reported results. The absence of standardized experimental approaches hampers the reproducibility and comparability of findings across studies.

## Supplementary Information

Below is the link to the electronic supplementary material.

Supplementary file1 (DOCX 2.08 MB)

## Data Availability

All source data for this work (or generated in this study) are available upon reasonable request.
