# Supplementary material for: The long antisense non-coding RNA HOXA transcript at the distal tip (LncRNA HOTTIP) in health and disease: a comprehensive review and in silico analysis
Source: Naunyn Schmiedebergs Arch Pharmacol. 2025 Jul 5;398(12):16537–75. doi: 10.1007/s00210-025-04372-9 (PMC12678630; doi:10.1007/s00210-025-04372-9)
Supplement: Supplementary file 1 — Supplementary file1 (DOCX 2.08 MB) [file 210_2025_4372_MOESM1_ESM.docx]

**Title Page**

**Article type:** Narrative non-systematic review

**The Long antisense Non-Coding RNA HOXA Transcript at the distal tip LncRNA**

**(HOTTIP) In Health and Disease; A Comprehensive Review and In Silico Analysis**

Mona G. El-Sisi^1^, Sara M. Radwan^1^, Sameh S. Ali^2^, Mohamed Y. Mostafa^3^, Nadia M. Hamdy^1, *^

^1^ Department of Biochemistry, Faculty of Pharmacy, Ain Shams University, 11566, Cairo, Egypt.

^2^ Research Department, Children’s Cancer Hospital Egypt-57357, Cairo, Egypt.

^3^ Department of Clinical Oncology, Faculty of Medicine, Ain Shams University, 11566, Cairo, Egypt.

*** Corresponding to** nadia_hamdy@pharma.asu.edu.eg, <https://orcid.org/0000-0003-2105-107X>.

| **A** | **B** | **C** |
| --- | --- | --- |
| **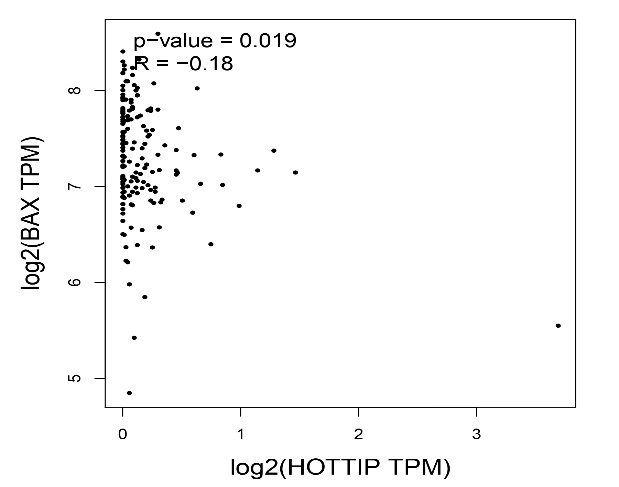** | **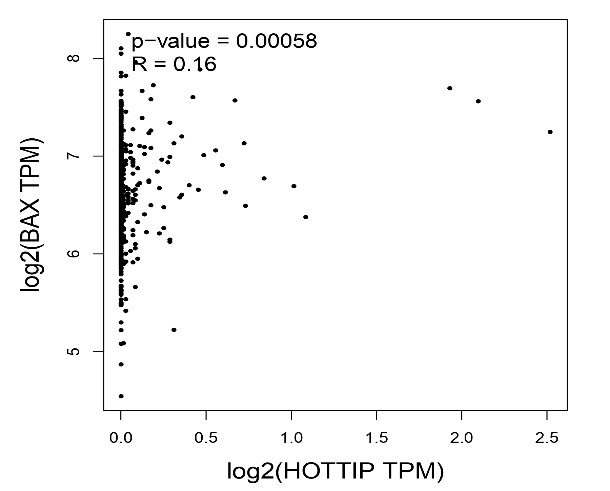** | **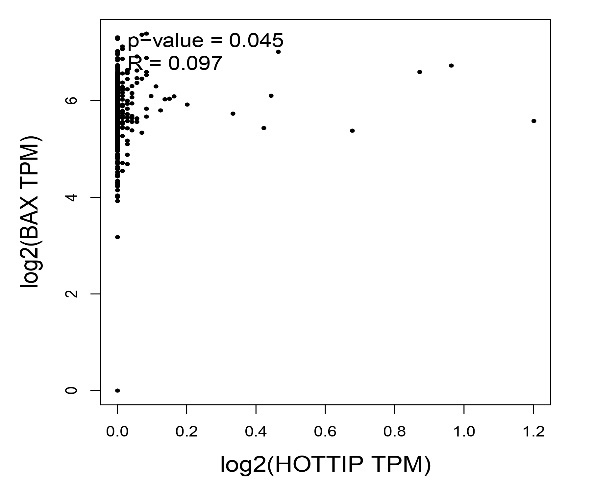** |
| **D** | **E** | **F** |
| **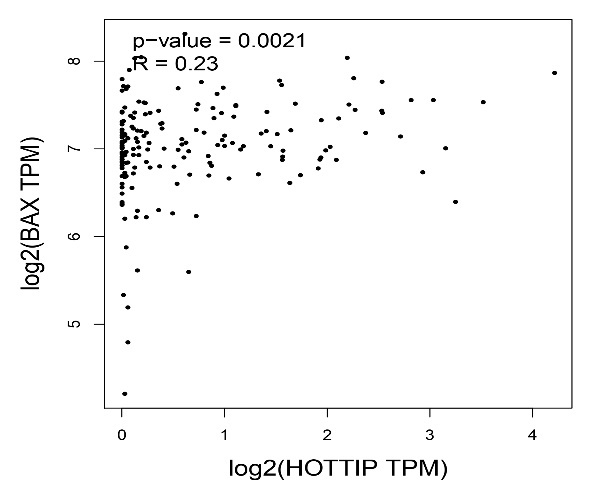** | **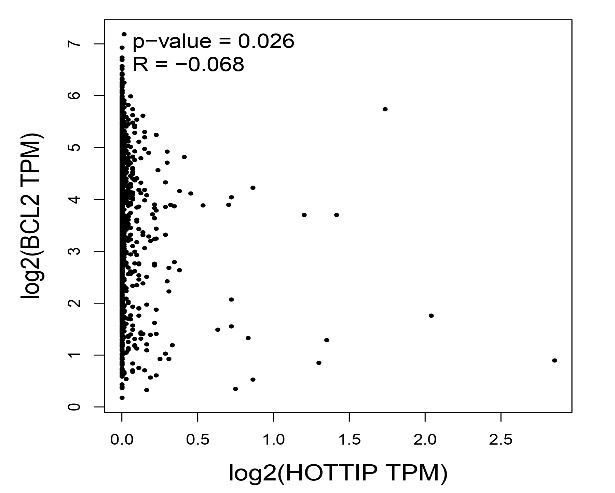** | **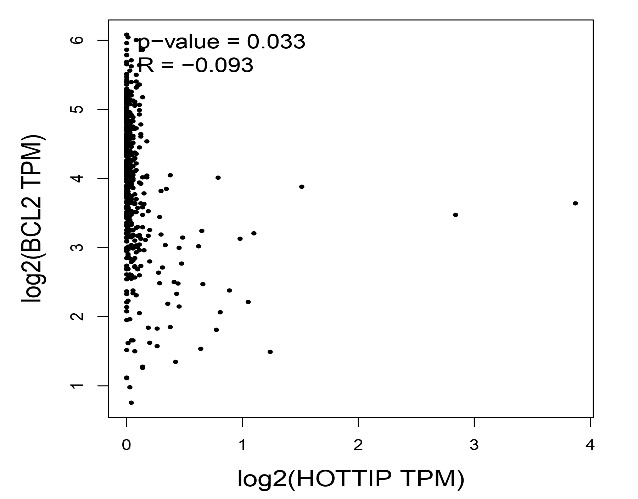** |
| **G** |  |  |
| **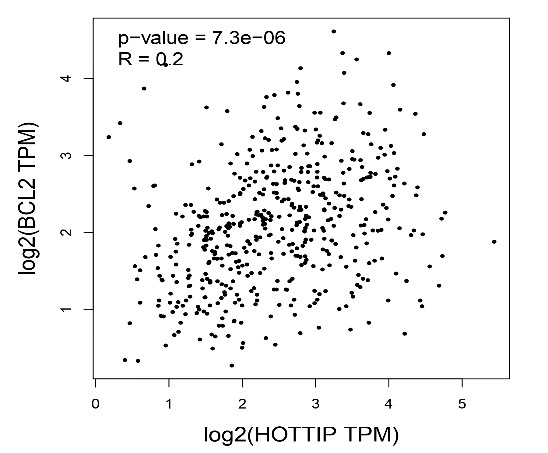** |  |  |

**Fig (S1). The correlation between HOTTIP and apoptotic markers in different cancers A) BAX in GBM, B) BAX in LUAD, C) BAX in OV, D) BAX in PAAD, E) BCL2 in BRCA, F) BCL2 in KIRC, G) BCL2 in PRAD.**

BAX, Bcl-2 Associated X-protein; BCL2, B-Cell Lymphoma 2; GBM, Glioblastoma; LUAD, Lung adenocarcinoma; OV, Ovarian serous cystadenocarcinoma; PAAD, Pancreatic adenocarcinoma; BRCA, Breast invasive carcinoma; KIRC, Kidney renal clear cell carcinoma; PRAD, Prostate adenocarcinoma.

| **A** | **B** |
| --- | --- |
| **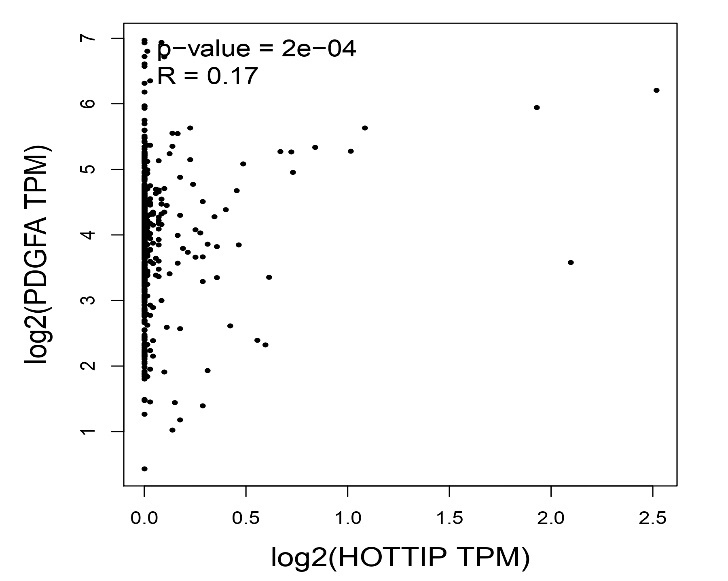** | **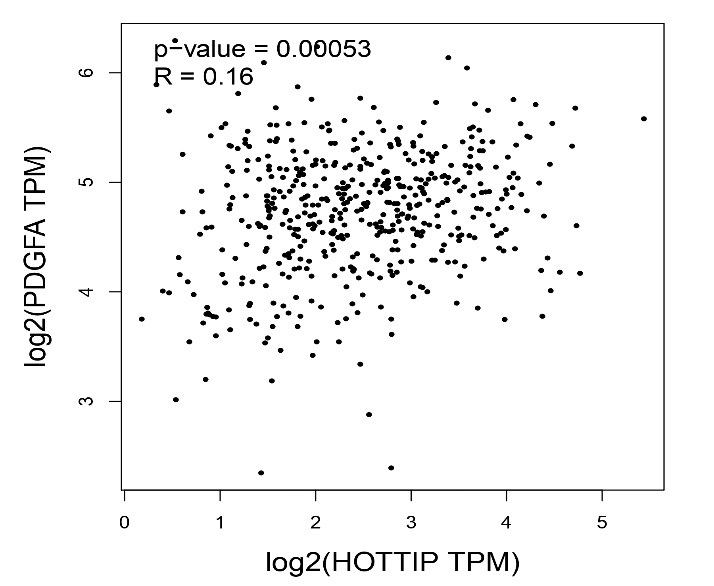** |
| **C** | **D** |
| **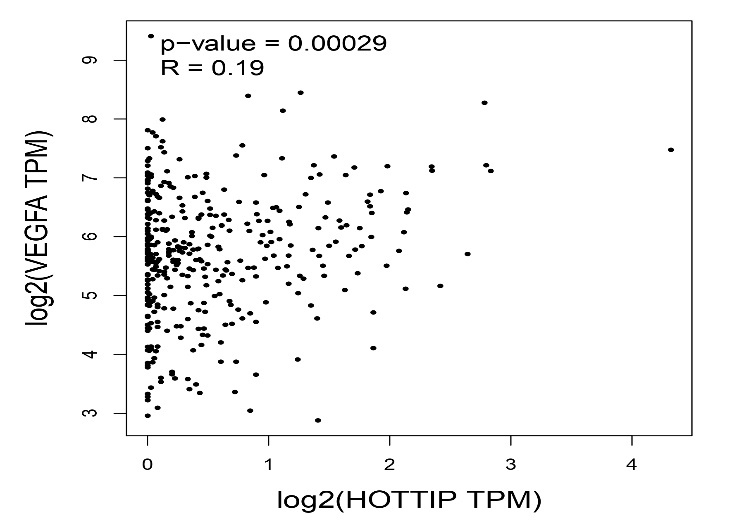** | **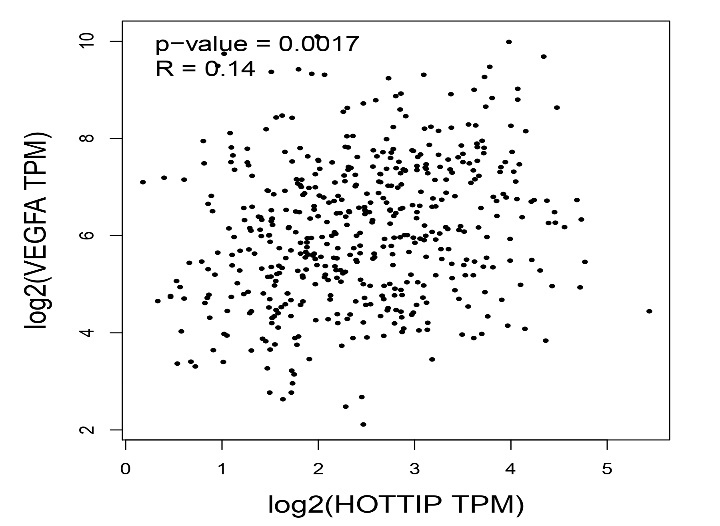** |

**Fig (S2). The correlation between HOTTIP and angiogenesis markers in different cancers A) PDGFA in LUAD, B) PDGFA in PRAD, C) VEGFA in LIHC, D) VEGFA in PRAD.**

PDGFA, Platelet-Derived Growth Factor Subunit A; VEGFA, Vascular Endothelial Growth Factor A; LUAD, Lung adenocarcinoma; PRAD, Prostate adenocarcinoma; LIHC, Liver hepatocellular carcinoma.

| **A** | **B** | **C** |
| --- | --- | --- |
| **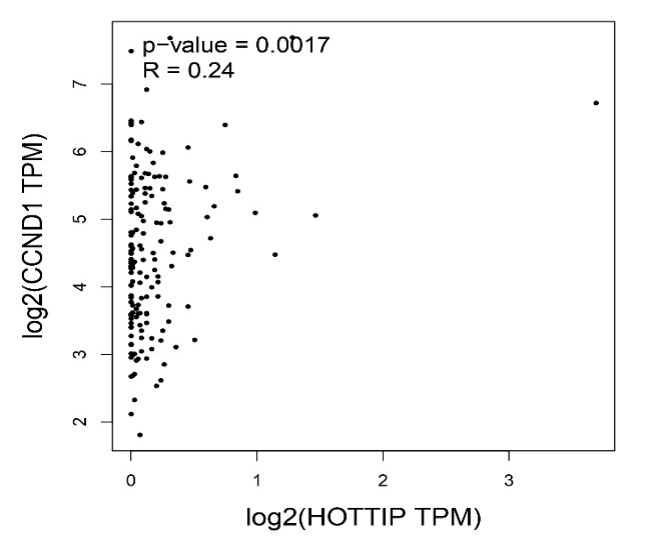** | 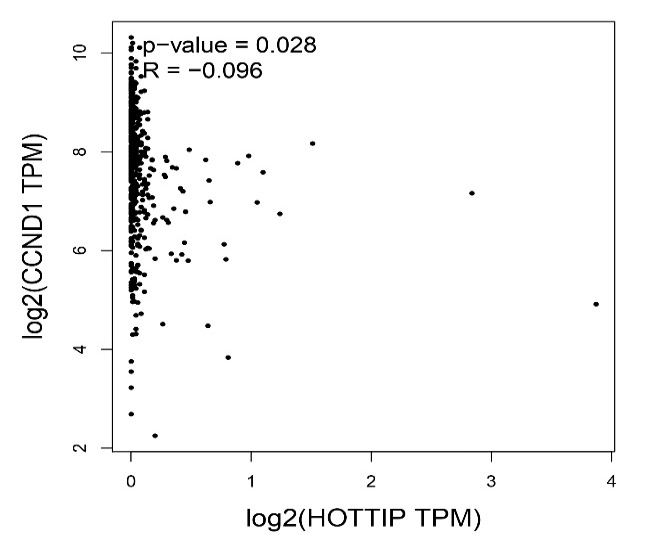 | 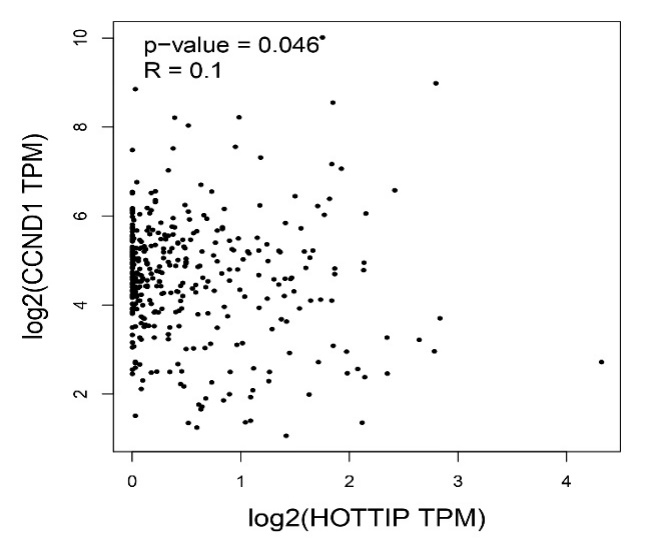 |
| **D** | **E** | **F** |
| **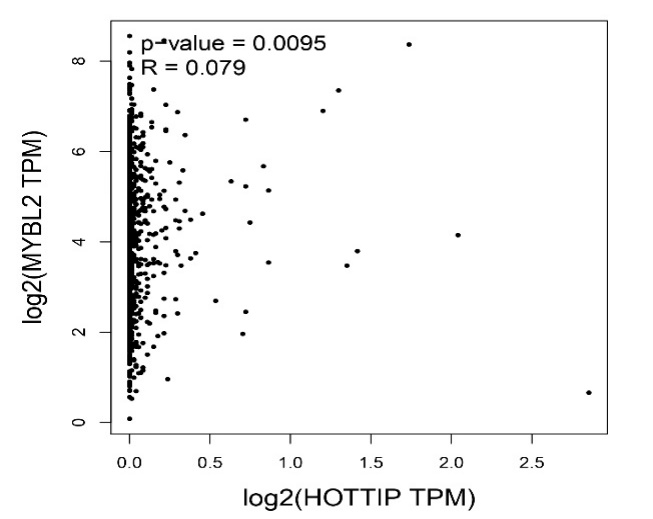** | **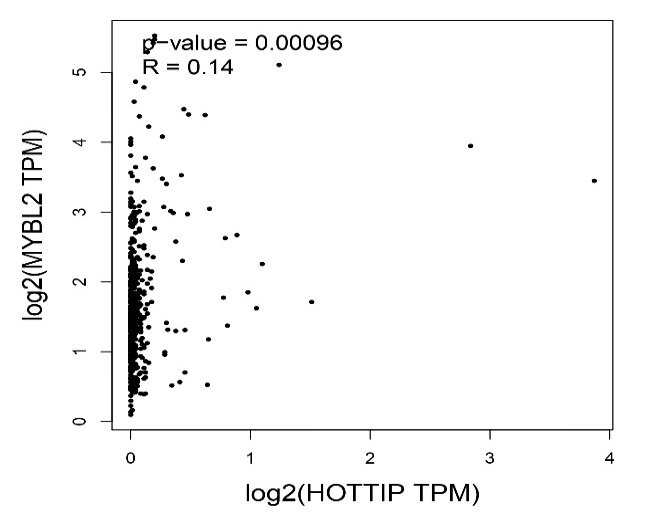** | **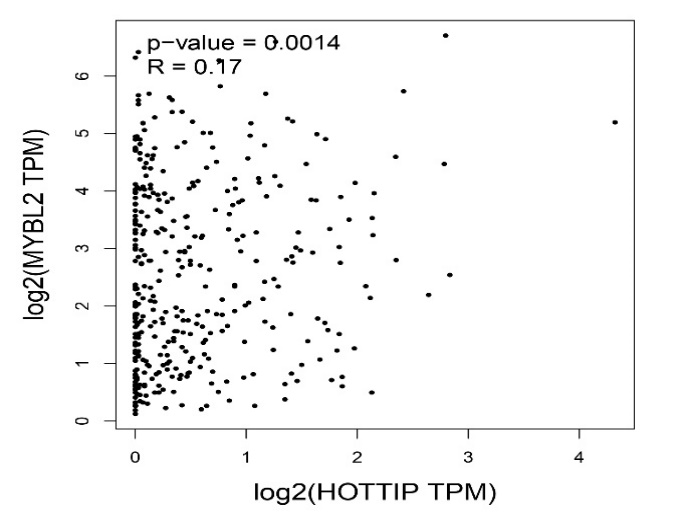** |

**Fig (S3). The correlation between HOTTIP and proliferation markers in different cancers A) CCND1 in GBM, B) CCND1 in KIRC, C) CCND1 in LIHC, D) MYBL2 in BRCA, E) MYBL2 in KIRC, F) MYBL2 in LIHC.**

CCND1, Cyclin D1; MYBL2, Myeloblastosis oncogene-like 2; GBM, Glioblastoma; KIRC, Kidney renal clear cell carcinoma; LIHC, Liver hepatocellular carcinoma; BRCA, Breast invasive carcinoma.

| **A** | **B** |
| --- | --- |
| 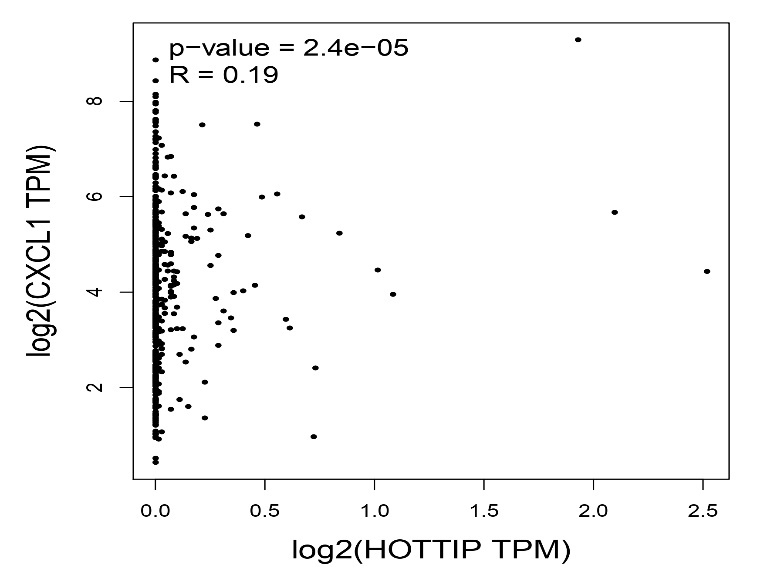 | 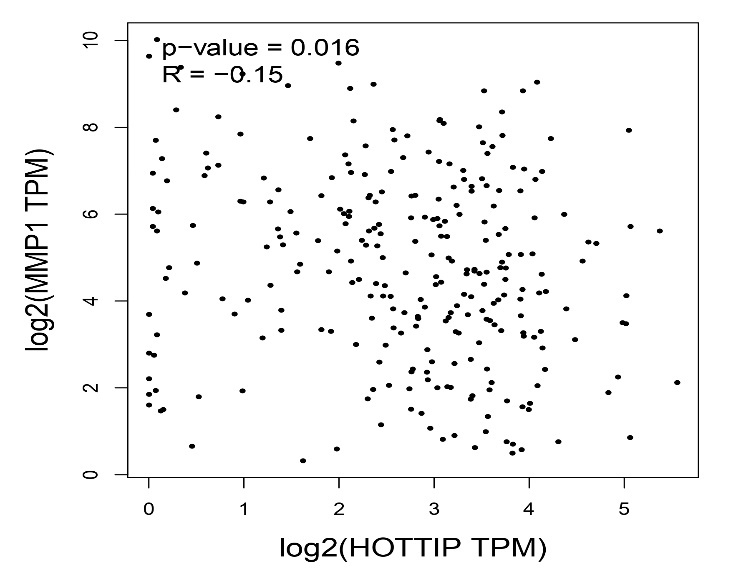 |

**Fig (S4). The correlation between HOTTIP and metastasis markers in different cancers A) CXCL1 in LUAD, B) MMP1 in COAD.**

CXCL1, C-X-C Motif Chemokine Ligand 1; MMP1, Matrix Metallopeptidase 1; LUAD, Lung adenocarcinoma; COAD, Colon adenocarcinoma.

As shown in **figures (S1), (S2), (S3) & (S4)**, there’s a lack of strong correlation between HOTTIP and some common apoptosis/angiogenesis/proliferation/metastasis markers (found by small correlation coefficients **Rs**). This may be attributed to the lack of sufficient studies or to the used small sample sizes. Thus, increasing the magnitude of Rs warrants more studies on larger sample sizes.

**
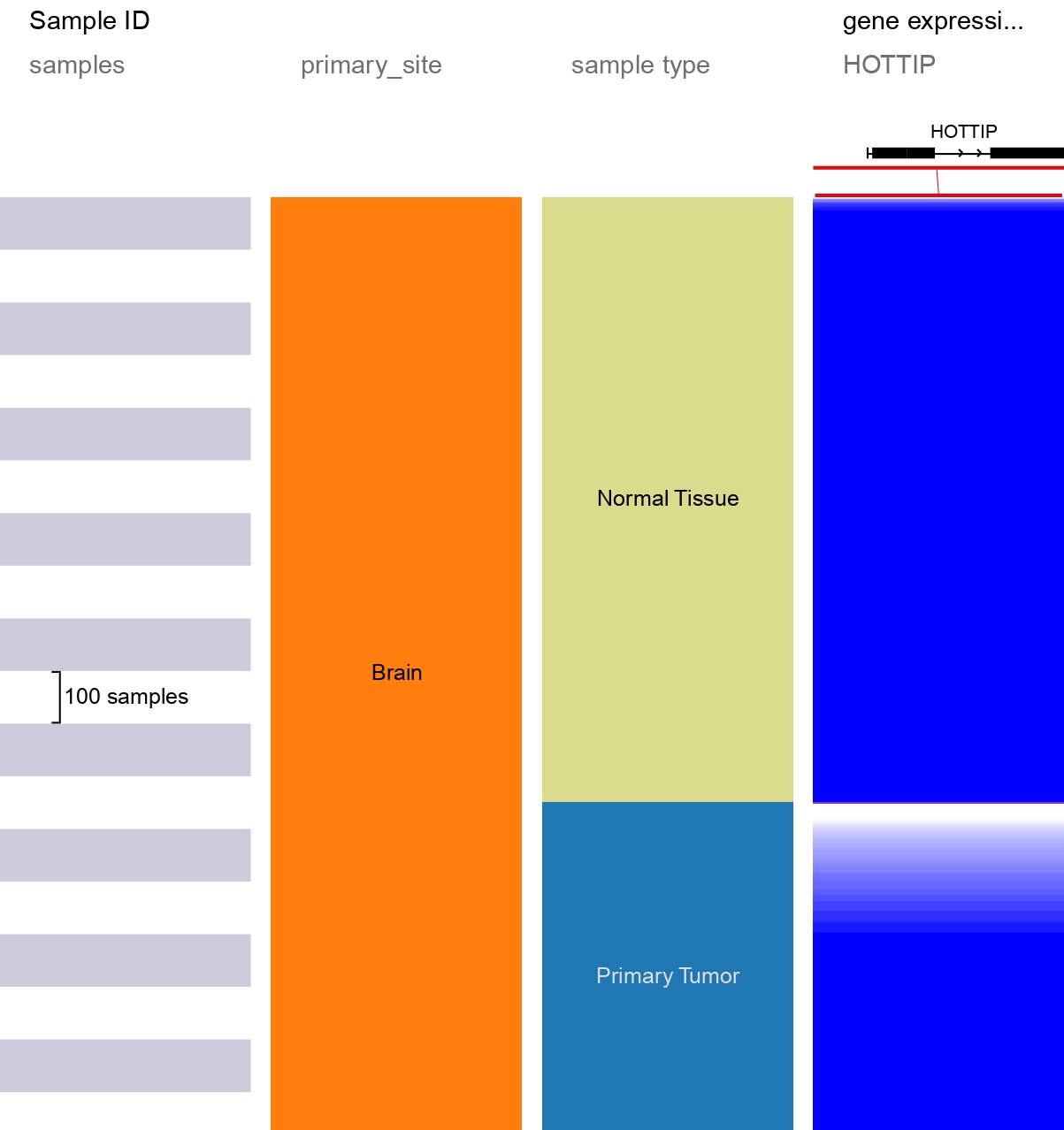
A**

**
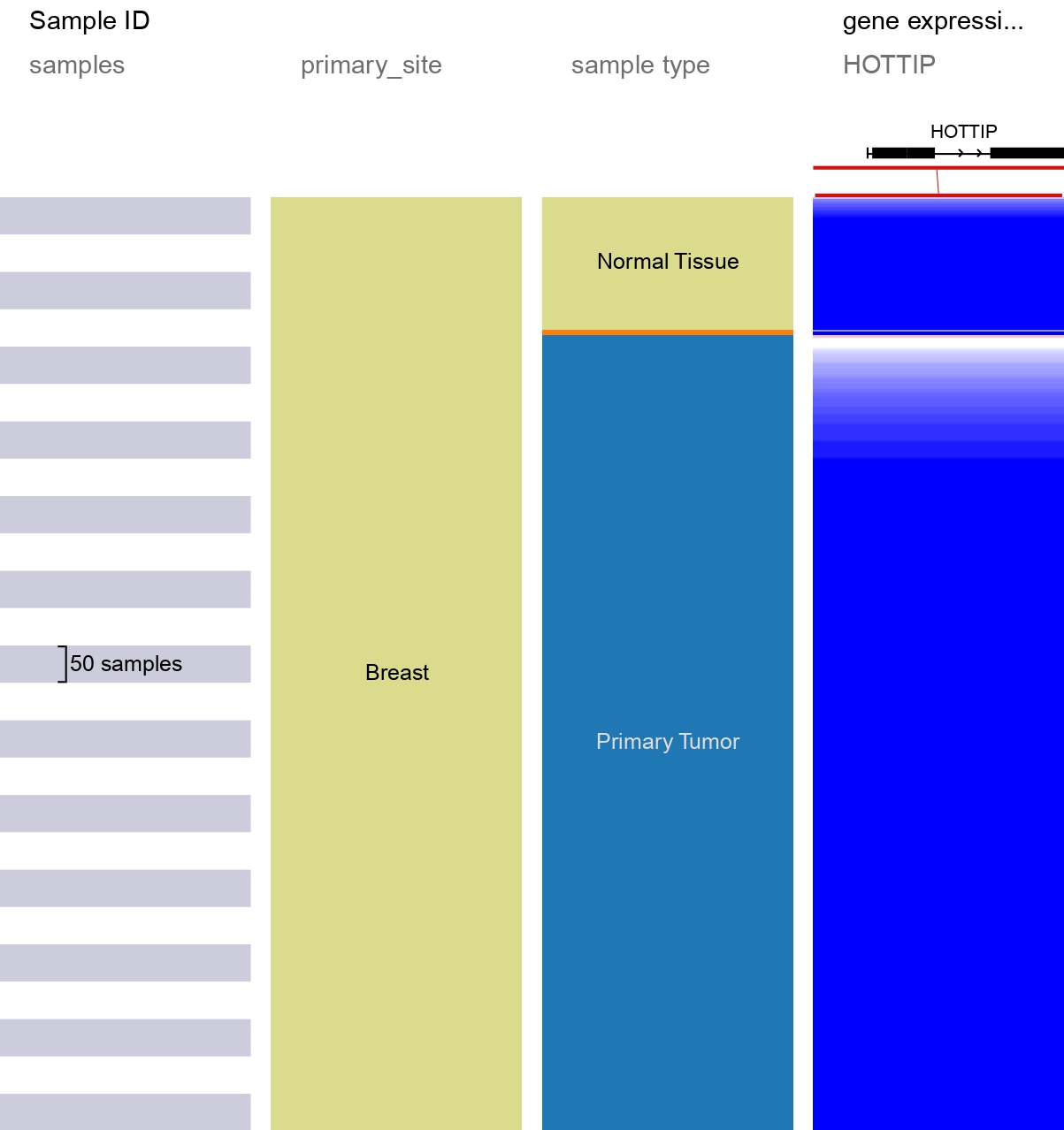
B**


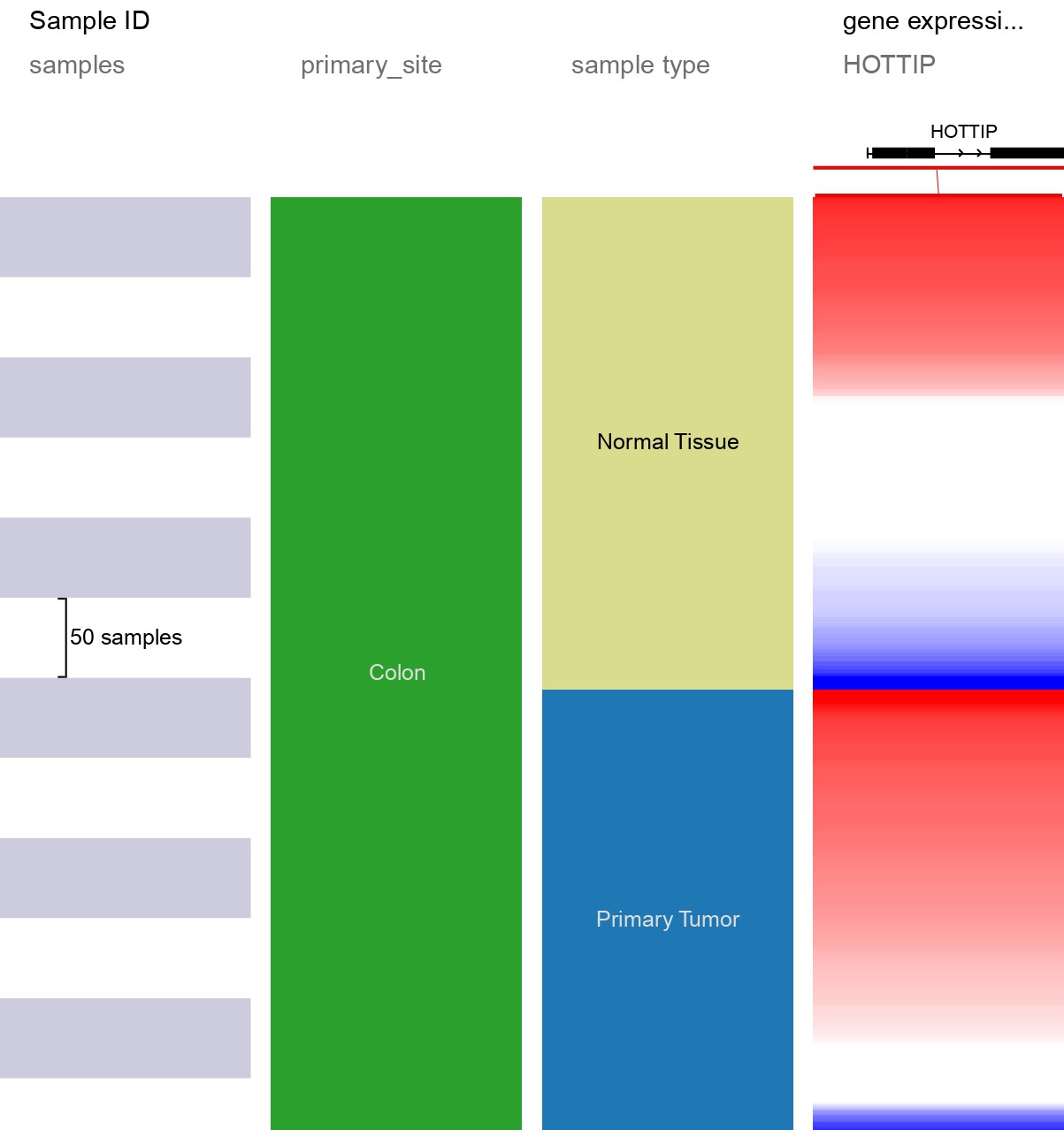
**C**

**D**


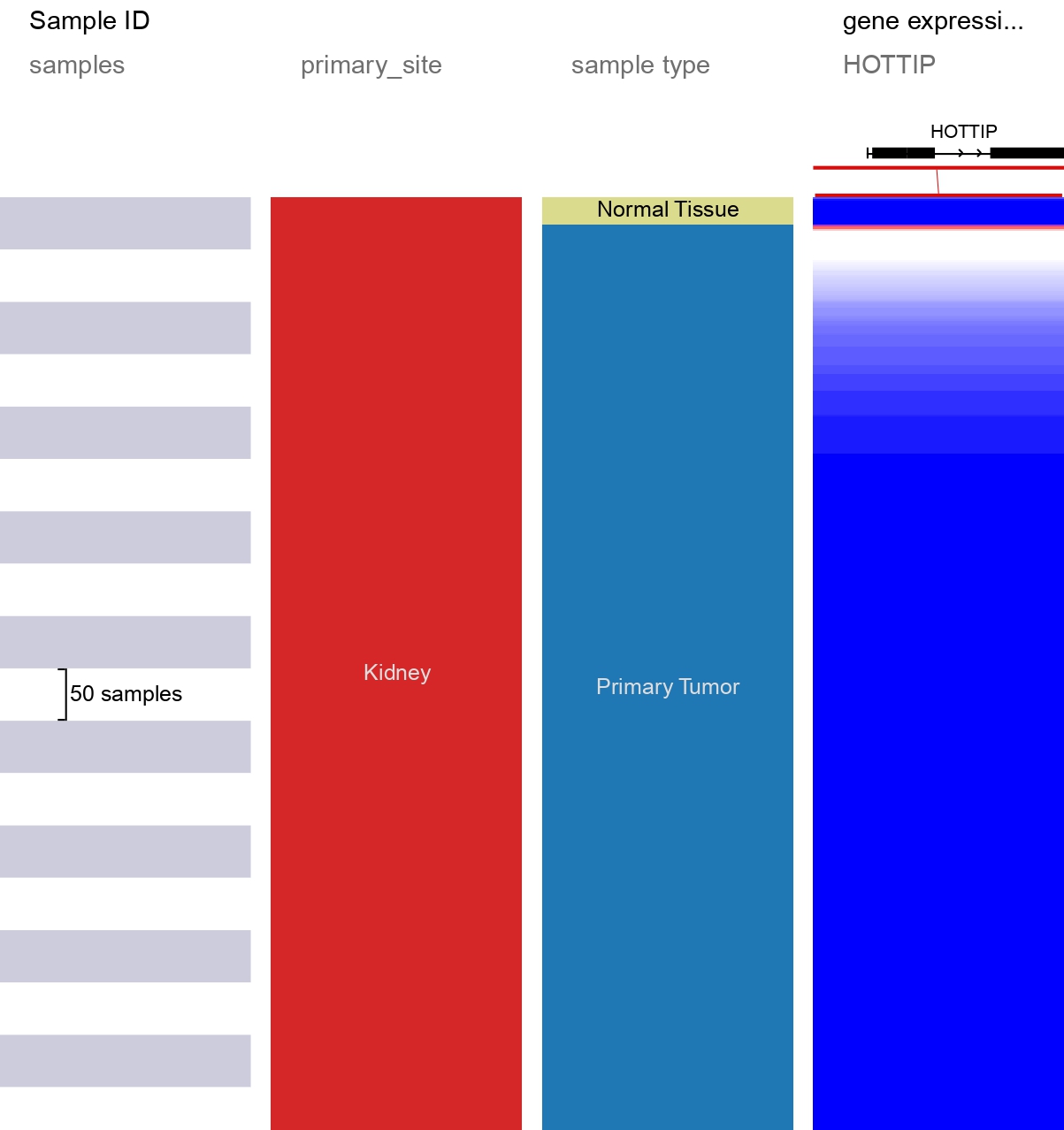


**E**


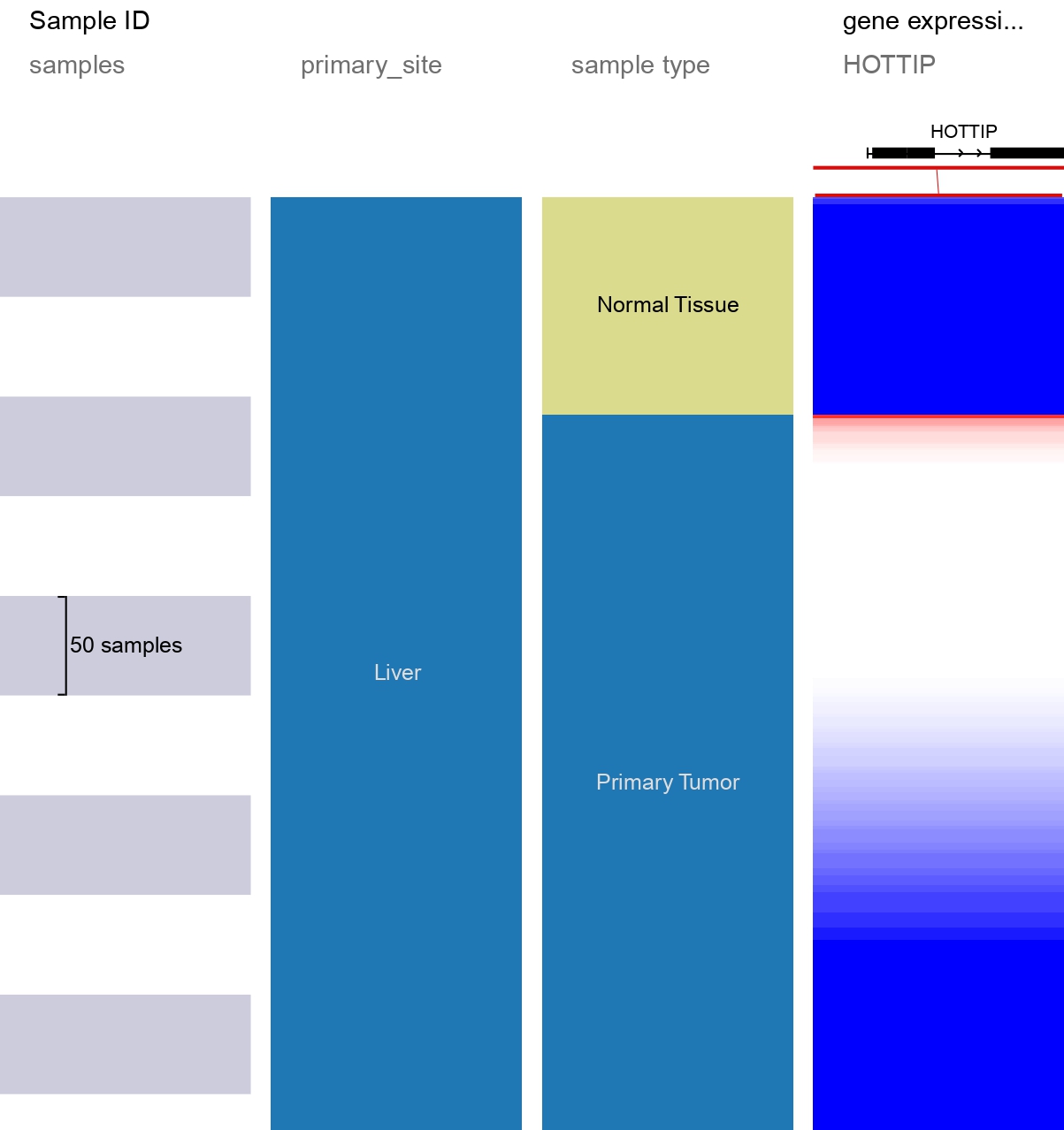


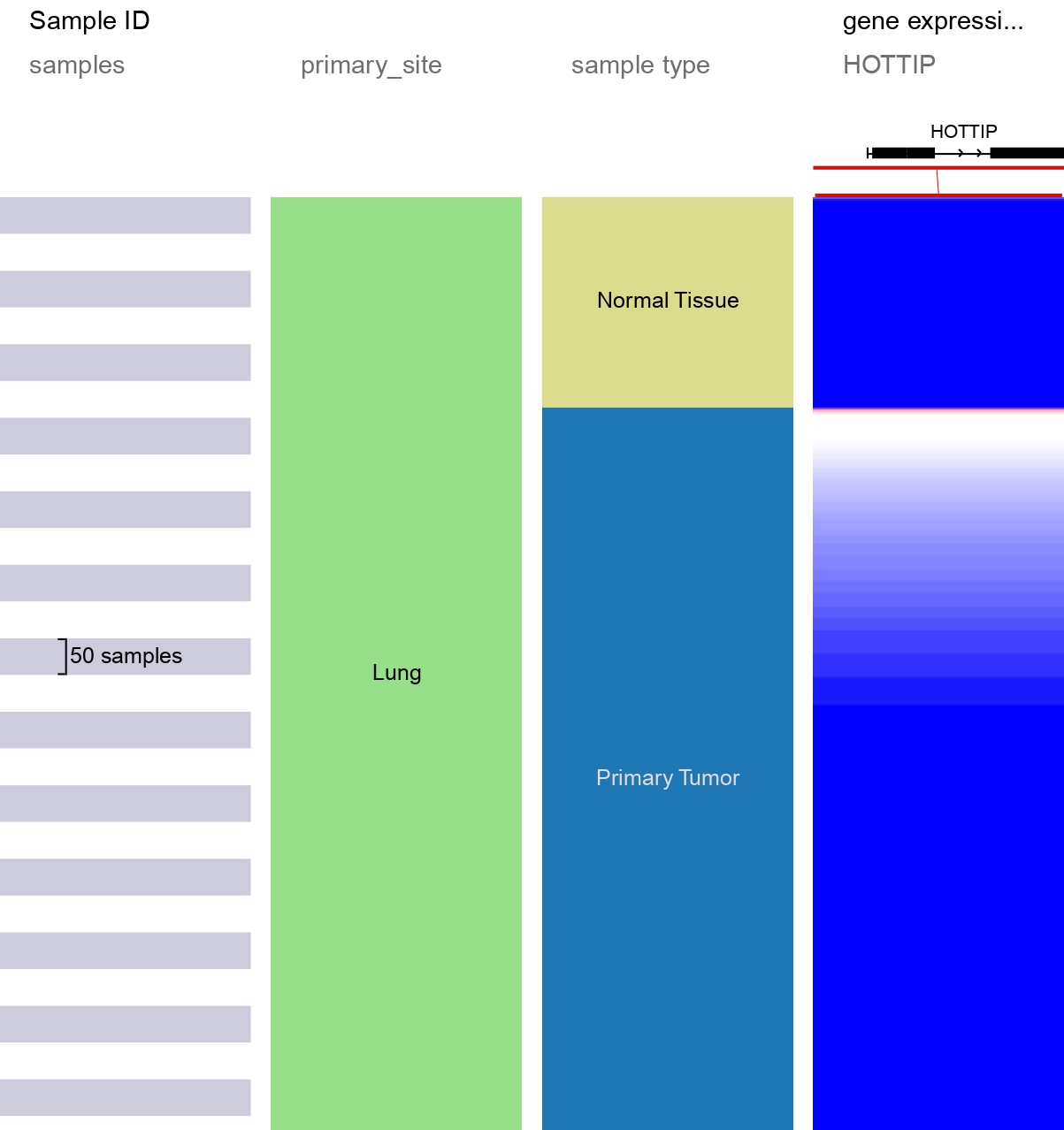
**F**

**
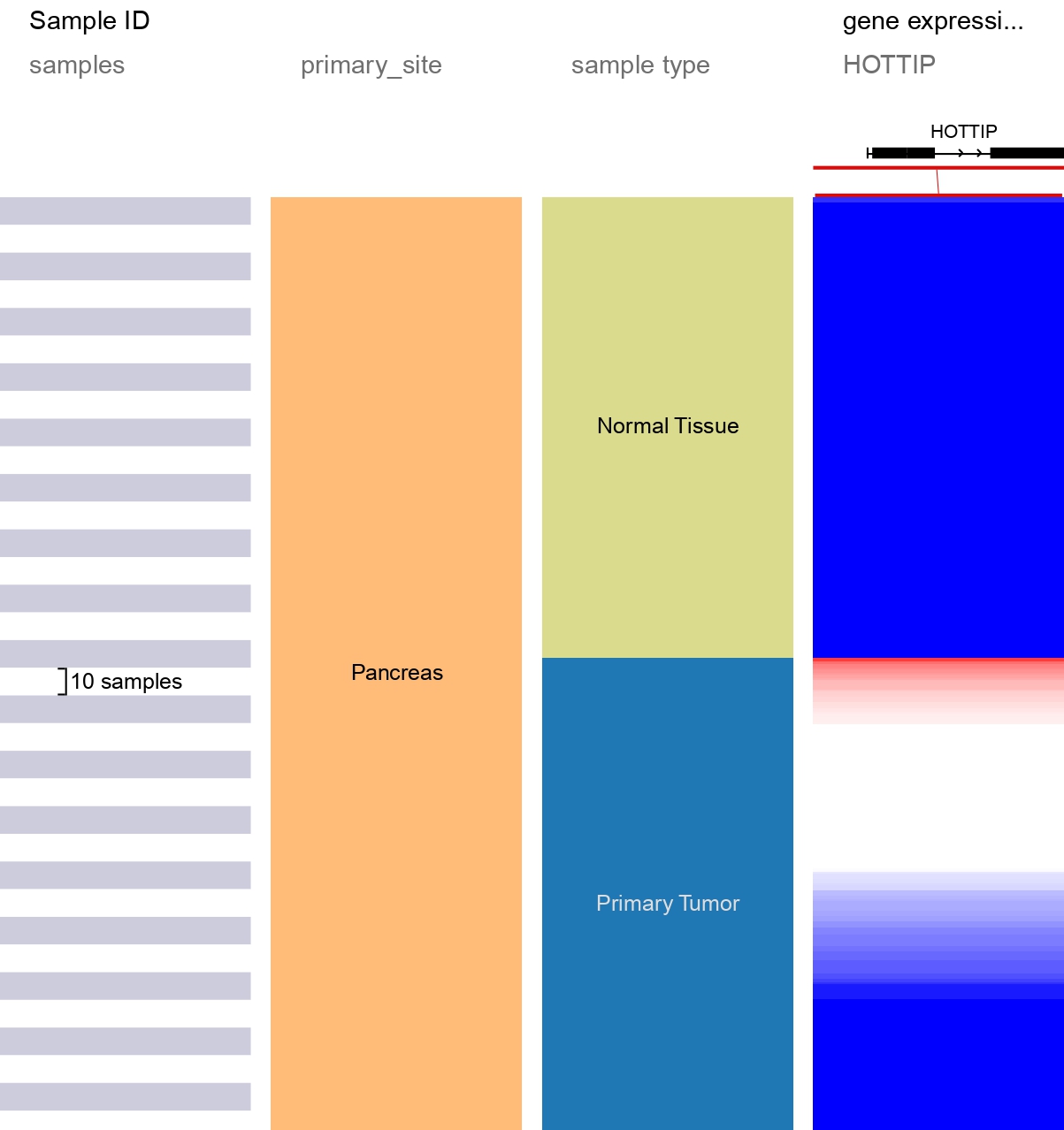
G**

**H**

**
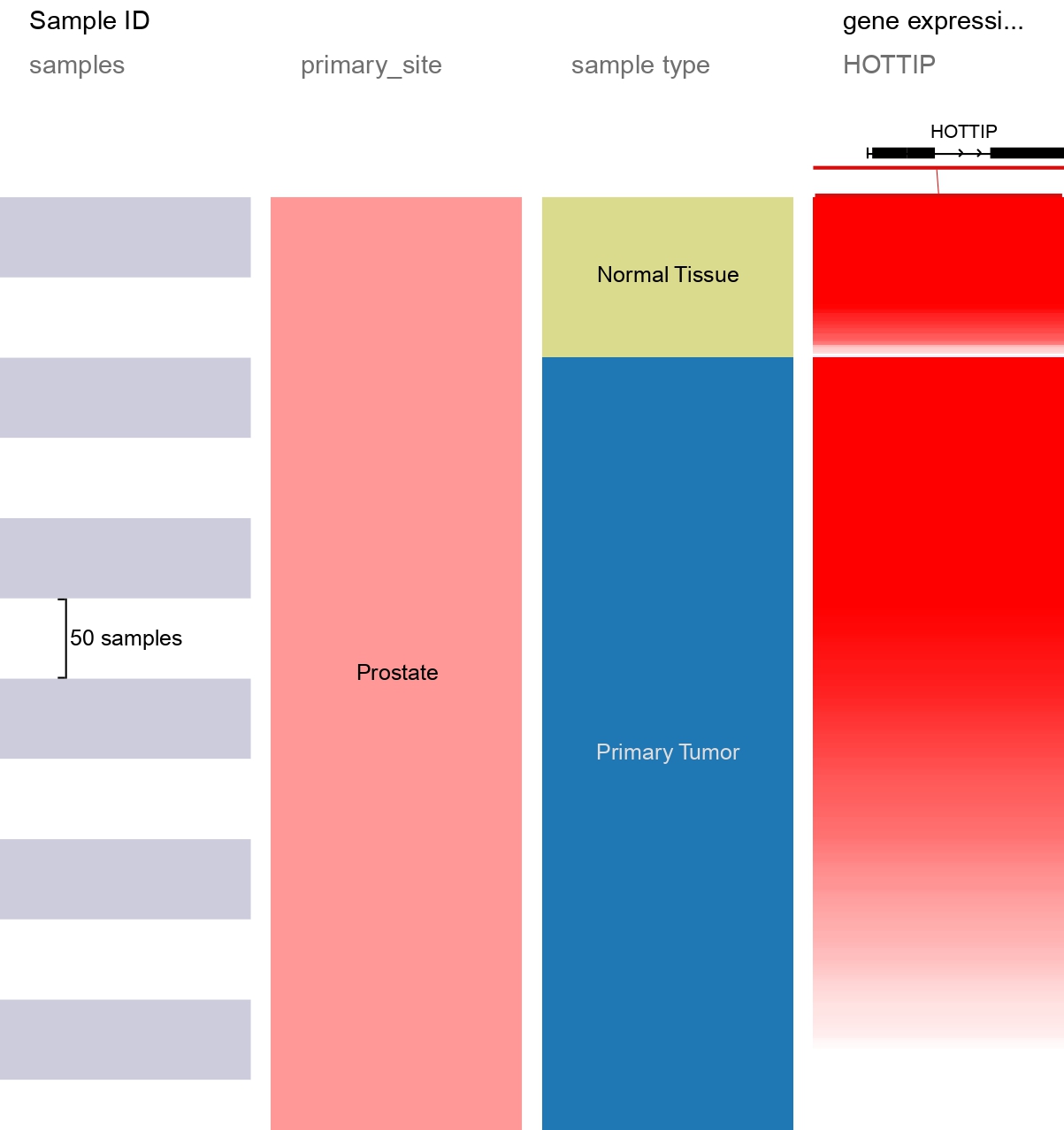
**

**Supplementary Fig (S5). Heatmaps showing the expression of HOTTIP in A) Brain primary tumor Vs Normal tissue, B) Breast primary tumor Vs Normal tissue, C) Colon primary tumor Vs Normal tissue, D) Kidney primary tumor Vs Normal tissue, E) Liver primary tumor Vs Normal tissue, F) Lung primary tumor Vs Normal tissue, G) Pancreatic primary tumor Vs Normal tissue, H) Prostate primary tumor Vs Normal tissue as generated by UCSC Xena web server.**

**Table (S1): Top 5 genes similar to HOTTIP in top 10 most common cancer with their Pearson Correlation Coefficients (PCC).**

| **Gene Symbol** | **Gene ID** | **Cancer** | **PCC*** |
| --- | --- | --- | --- |
| [***HOXA13***](http://gepia.cancer-pku.cn/detail.php?gene=HOXA13) | ENSG00000106031.7 | BRCA | 0.88 |
| [***RP1-170O19.14***](http://gepia.cancer-pku.cn/detail.php?gene=RP1-170O19.14) | ENSG00000253508.1 | BRCA | 0.78 |
| [***SNORA31***](http://gepia.cancer-pku.cn/detail.php?gene=SNORA31) | ENSG00000252888.1 | BRCA | 0.75 |
| [***SPHAR***](http://gepia.cancer-pku.cn/detail.php?gene=SPHAR) | ENSG00000213029.3 | BRCA | 0.66 |
| [***ATP6V0E2-AS1***](http://gepia.cancer-pku.cn/detail.php?gene=ATP6V0E2-AS1) | ENSG00000204934.10 | BRCA | 0.57 |
| ***HOXA13*** | ENSG00000106031.7 | COAD | 0.74 |
| ***RP1-170O19.14*** | ENSG00000253508.1 | COAD | 0.66 |
| ***HOXA11-AS*** | ENSG00000240990.9 | COAD | 0.62 |
| ***HOXA11-AS1_5*** | ENSG00000278592.1 | COAD | 0.46 |
| ***HOXA11*** | ENSG00000005073.5 | COAD | 0.42 |
| [***MAGEA9B***](http://gepia.cancer-pku.cn/detail.php?gene=MAGEA9B) | ENSG00000267978.5 | GBM | 0.97 |
| ***LINC02735*** | ENSG00000255433.5 | GBM | 0.97 |
| [***PRR20B***](http://gepia.cancer-pku.cn/detail.php?gene=PRR20B) | ENSG00000204918.3 | GBM | 0.97 |
| [***PRR20A***](http://gepia.cancer-pku.cn/detail.php?gene=PRR20A) | ENSG00000204919.1 | GBM | 0.97 |
| [***PRR20D***](http://gepia.cancer-pku.cn/detail.php?gene=PRR20D) | ENSG00000227151.4 | GBM | 0.97 |
| [***HTR1D***](http://gepia.cancer-pku.cn/detail.php?gene=HTR1D) | ENSG00000179546.4 | KIRC | 0.96 |
| ***[TRMT112P4](http://gepia.cancer-pku.cn/detail.php?gene=TRMT112P4)*** | ENSG00000236763.2 | KIRC | 0.95 |
| [***CXADRP2***](http://gepia.cancer-pku.cn/detail.php?gene=CXADRP2) | ENSG00000258712.2 | KIRC | 0.94 |
| [***AMER3***](http://gepia.cancer-pku.cn/detail.php?gene=AMER3) | ENSG00000178171.10 | KIRC | 0.94 |
| [***AMER2***](http://gepia.cancer-pku.cn/detail.php?gene=AMER2) | ENSG00000165566.12 | KIRC | 0.93 |
| [***HOXA13***](http://gepia.cancer-pku.cn/detail.php?gene=HOXA13) | ENSG00000106031.7 | LAML | 0.93 |
| [***RHOXF2B***](http://gepia.cancer-pku.cn/detail.php?gene=RHOXF2B) | ENSG00000203989.3 | LAML | 0.85 |
| [***EPYC***](http://gepia.cancer-pku.cn/detail.php?gene=EPYC) | ENSG00000083782.7 | LAML | 0.84 |
| [***TRIM53CP***](http://gepia.cancer-pku.cn/detail.php?gene=TRIM53CP) | ENSG00000254764.1 | LAML | 0.84 |
| [***FMO11P***](http://gepia.cancer-pku.cn/detail.php?gene=FMO11P) | ENSG00000232148.1 | LAML | 0.84 |
| ***HSDL2-AS1*** | ENSG00000230185.4 | LIHC | 0.72 |
| [***TM4SF20***](http://gepia.cancer-pku.cn/detail.php?gene=TM4SF20) | ENSG00000168955.3 | LIHC | 0.72 |
| [***HOXA13***](http://gepia.cancer-pku.cn/detail.php?gene=HOXA13) | ENSG00000106031.7 | LIHC | 0.70 |
| [***PRSS41***](http://gepia.cancer-pku.cn/detail.php?gene=PRSS41) | ENSG00000215148.7 | LIHC | 0.70 |
| [***SEPT14P1***](http://gepia.cancer-pku.cn/detail.php?gene=SEPT14P1) | ENSG00000231523.1 | LIHC | 0.70 |
| [***HOXA13***](http://gepia.cancer-pku.cn/detail.php?gene=HOXA13) | ENSG00000106031.7 | LUAD | 0.85 |
| [***RP1-170O19.14***](http://gepia.cancer-pku.cn/detail.php?gene=RP1-170O19.14) | ENSG00000253508.1 | LUAD | 0.81 |
| [***HOXA11-AS1_5***](http://gepia.cancer-pku.cn/detail.php?gene=HOXA11-AS1_5) | ENSG00000278592.1 | LUAD | 0.71 |
| [***AC002486.2***](http://gepia.cancer-pku.cn/detail.php?gene=AC002486.2) | ENSG00000231525.1 | LUAD | 0.70 |
| [***ASIC2***](http://gepia.cancer-pku.cn/detail.php?gene=ASIC2) | ENSG00000108684.14 | LUAD | 0.70 |
| [***HOXA13***](http://gepia.cancer-pku.cn/detail.php?gene=HOXA13) | ENSG00000106031.7 | OV | 0.92 |
| [***RP1-170O19.14***](http://gepia.cancer-pku.cn/detail.php?gene=RP1-170O19.14) | ENSG00000253508.1 | OV | 0.82 |
| [***HOXA11***](http://gepia.cancer-pku.cn/detail.php?gene=HOXA11) | ENSG00000005073.5 | OV | 0.78 |
| [***HOXA11-AS1_6***](http://gepia.cancer-pku.cn/detail.php?gene=HOXA11-AS1_6) | ENSG00000278020.1 | OV | 0.74 |
| [***ZFHX4-AS1***](http://gepia.cancer-pku.cn/detail.php?gene=ZFHX4-AS1) | ENSG00000253661.1 | OV | 0.72 |
| [***HOXA13***](http://gepia.cancer-pku.cn/detail.php?gene=HOXA13) | ENSG00000106031.7 | PAAD | 0.88 |
| [***HOXA11-AS***](http://gepia.cancer-pku.cn/detail.php?gene=HOXA11-AS) | ENSG00000240990.9 | PAAD | 0.81 |
| [***HOXA11***](http://gepia.cancer-pku.cn/detail.php?gene=HOXA11) | ENSG00000005073.5 | PAAD | 0.71 |
| [***HOXA11-AS1_5***](http://gepia.cancer-pku.cn/detail.php?gene=HOXA11-AS1_5) | ENSG00000278592.1 | PAAD | 0.68 |
| [***HOXA9***](http://gepia.cancer-pku.cn/detail.php?gene=HOXA9) | ENSG00000078399.15 | PAAD | 0.66 |
| [***HOXA13***](http://gepia.cancer-pku.cn/detail.php?gene=HOXA13) | ENSG00000106031.7 | PRAD | 0.69 |
| [***RP1-170O19.14***](http://gepia.cancer-pku.cn/detail.php?gene=RP1-170O19.14) | ENSG00000253508.1 | PRAD | 0.53 |
| [***HOXA11-AS1_5***](http://gepia.cancer-pku.cn/detail.php?gene=HOXA11-AS1_5) | ENSG00000278592.1 | PRAD | 0.41 |
| [***PBX2***](http://gepia.cancer-pku.cn/detail.php?gene=PBX2) | ENSG00000204304.11 | PRAD | 0.41 |
| [***TTC31***](http://gepia.cancer-pku.cn/detail.php?gene=TTC31) | ENSG00000115282.19 | PRAD | 0.40 |

*All the correlations are significant at p-value > 0.001

HOXA, Homeobox A; BRCA, Breast invasive carcinoma; RP1-170O19.14, Retinitis Pigmentosa-1-170O19.14; SNORA31, Small Nucleolar RNA, H/ACA Box 31; SPHAR, S-phase response; ATP6V0E2-AS1, ATPase H+ transporting V0 subunit e2 anti-sense 1; COAD, Colon adenocarcinoma; HOXA11-AS, Homeobox A11- antisense; MAGEA9B, Melanoma-associated Antigen 4 family member A9B ; GBM, Glioblastoma; LINC02735, Long Intergenic Non-Protein Coding RNA 2735; PRR20A, Proline-rich protein 20A; HTR1D, 5-Hydroxytryptamine Receptor 1D; KIRC, Kidney renal clear cell carcinoma; TRMT112P4, tRNA methyltransferase subunit 11-2 pseudogene 4; CXADRP2, Coxsackie virus and adenovirus receptor pseudogene 2; AMER, APC Membrane Recruitment Protein; LAML, Acute Myeloid Leukemia; RHOXF2B, Rhox Homeobox Family Member 2B; EPYC, Epiphycan; TRIM53CP Tripartite Motif containing 53C Pseudogene; FMO11P, Flavin Containing Dimethylaniline Monoxygenase 11 Pseudogene; HSDL2-AS1, Hydroxysteroid Dehydrogenase Like 2- antisense 1; LIHC, Liver hepatocellular carcinoma; TM4SF20, Transmembrane 4 L Six Family Member 20; PRRS41, Serine protease 41; SEPT14P1, Septin 14 pseudogene 1; LUAD, Lung adenocarcinoma; ASIC2, Acid Sensing Ion Channel Subunit 2; OV, Ovarian serous cystadenocarcinoma; ZFHX4-AS1, Zinc Finger Homeobox 4- antisense1; PAAD, Pancreatic adenocarcinoma; PRAD, Prostate adenocarcinoma; PBX2, Pre B cell leukemia homeobox 2; TTC31, Tetratricopeptide Repeat Domain 31.

This table shows that there’s a strong significant similarity between *HOTTIP & HOXA13, RP1-170O19.14, SNORA31* and *SPHAR* while there’s an intermediate significant similarity between *HOTTIP & ATP6V0E2-AS1* in BRCA. In COAD, there’s a strong significant similarity between *HOTTIP & HOXA13, RP1-170O19.14* and *HOXA11-AS* while there’s an intermediate significant similarity between *HOTTIP & HOXA11-AS1_5* and *HOXA11*.

For GBM, there’s a strong significant similarity between *HOTTIP &MAGEA9B, LINC02735, PRR20B, PRR20A* and *PRR20D*. This applies to KIRC where there’s a strong significant similarity between *HOTTIP & HTR1D, TRMT112P4, CXADRP2, AMER3* and *AMER2* as well. This is also applicable in LAML as there’s a strong significant similarity between *HOTTIP & HOXA13, RHOXF2B, EPYC, TRIM53CP* and *FMO11P.*

Regarding LUAD, there’s also a strong significant similarity between *HOTTIP & HOXA13, RP1-170O19.14, HOXA11-AS1_5, AC002486.2* and *ASIC2*. For OV, the 5 genes (*HOXA13, RP1-170O19.14, HOXA11, HOXA11-AS1_6* and *ZFHX4-AS1*) are strongly similar to HOTTIP in a significant manner. *HOXA13, HOXA11, HOXA11-AS, HOXA11-AS1_5* and *HOXA9* genes are all significantly similar to *HOTTIP* in PAAD. Finally, *HOXA13* gene is highly similar to *HOTTIP* while *HOXA11-AS1_5, RP1-170O19.14, PBX2* and *TTC31* genes are moderately similar to *HOTTIP* in PRAD.

**List of abbreviations:**

ABCG2: ATP-Binding Cassette sub-family G member 2

ADAMTS-4: A Disintegrin and Metalloprotease with Thrombospondin motifs 4

ADAMTS-5 A: Disintegrin and Metalloprotease with Thrombospondin motifs 5

ADM: Adriamycin

AGA: Acute Gouty Arthritis

AKT: Protein Kinase B

ALS: Amyotrophic Lateral Sclerosis

AMER: APC Membrane Recruitment Protein

AMI: Acute myocardial infarction

AML: Acute Myeloid Leukemia

AP: Adenomatous Polyposis

ARDS: Acute Respiratory Distress Syndrome

AS: Ankylosing Spondylitis

ASFLSs: AS Fibroblast-Like Synoviocytes

ASIC2: Acid Sensing Ion Channel Subunit 2

ASK1: Apoptosis Signal-regulating Kinase 1

Atg13: Autophagy Related 13

ATP6V0E2-AS1: ATPase H+ transporting V0 subunit e2 anti-sense 1

BAD: BCL2 associated Agonist of cell Death

BAX: Bcl-2 Associated X-protein

BC: Breast Cancer

BCL2: B-Cell Lymphoma 2

BMD: Bone Mineral Density

BMSCs: Bone marrow Mesenchymal Stem Cells

BRCA: Breast invasive carcinoma

CAD: Coronary Artery Disease

CC: Cervical Cancer

CCA: Cholangiocarcinoma

CCL3: C-C Motif Chemokine Ligand 3

CCND1: Cyclin D1

CDK2: Cyclin-Dependent Kinase 2

ceRNA: Competing Endogenous RNA

CHF: Congestive Heart Failure

ChIP-seq: Chromatin immunoprecipitation sequencing

CML: Chronic Myeloid Leukemia

c-Myc: cellular Myelocytomatosis

COAD: Colon adenocarcinoma

COL2A1: Collagen Type II Alpha 1 Chain

CRC: Colorectal Cancer

CREB1: cAMP-Responsive Element-Binding protein 1

CSCs: Cancer Stem Cells

CTCF: CCCTC binding factor

CXADRP2: Coxsackie virus and adenovirus receptor pseudogene 2

CXCL1: C-X-C Motif Chemokine Ligand 1

DDA1: DET1- and DDB1-Associated Protein 1

DDR: DNA Damage Response

DKK1: Dickkopf WNT Signaling Pathway Inhibitor 1

DLX2: Distal-Less Homeobox 2

DN: Diabetic Nephropathy

DNA pol: Deoxy Nucleic Acid Polymerase

DNA: Deoxy Nucleic acid

DNMT1: DNA MethylTransferase 1

EC: Endometrial Cancer

EFNA3: Ephrin A3

EMC: Extracellular Matrix

EMT: Epithelial–Mesenchymal Transition

EPO: Erythropoietin

EPYC: Epiphycan

ERK: Extracellular signal–Regulated Kinase

EZH1: Enhancer of Zeste Homologous protein 1

EZH2: Enhancer of Zeste Homologous protein 2

FLS: Fibroblast-Like Synoviocytes

FMO11P: Flavin Containing Dimethylaniline Monoxygenase 11 Pseudogene

FOXO1: Forkhead box O1

FOXO3: Forkhead box O3

G-6-Pase: Glucose-6-Phosphatase

GBM: Glioblastoma

GC: Gastric Cancer

GDM: Gestational Diabetes Mellitus

GLS1: Glutaminase 1

GLUT2: Glucose Transporter 2

GNG12: G protein subunit Gamma 12

GTF3C2: General Transcription Factor IIIC Subunit 2

HBV: Hepatitis B Virus

HCC: Hepatocellular Carcinoma

HCV: hepatitis C virus

HIF-1α: Hypoxia-Inducible Factor 1-alpha

HMGA1: High-Mobility Group A1

HMGA2: High-Mobility Group AT-hook 2

HMGB1: High Mobility Group Box 1

hnRNPA2B1: heterogeneous nuclear Ribonucleoprotein A2B1

HNSCC: Head and Neck Squamous Cell Carcinoma

HOTAIR: HOX Transcript Antisense RNA

HOTTIP: HOXA transcript at the distal tip

HOXA: Homeobox A

HOXA11-AS: Homeobox A11- antisense

HSCR: Hirschsprung Disease

HSCs: Hepatic Stellate Cells

HSDL2-AS1: Hydroxysteroid Dehydrogenase Like 2- antisense 1

HTR1D: 5-Hydroxytryptamine Receptor 1D

IDD: Intervertebral Disc Degeneration

IGF‑2: Insulin‑like Growth Factor-2

IGFBP-3: Insulin Growth Factor-Binding Protein 3

IL-1β: Interleukin-1β

IL-6: Interleukin-6

IM: Imatinib Mesylate

IRSA: Idiopathic Recurrent Spontaneous Abortion

JNK: c-Jun N-terminal Kinase

KHSRP: KH-Type Splicing Regulatory Protein

KIRC: Kidney renal clear cell carcinoma

KOA: Knee Osteoarthritis

LA: Lung Adenocarcinoma

LAML: Acute Myeloid Leukemia

LIHC: Liver hepatocellular carcinoma

LINC02735: Long Intergenic Non-Protein Coding RNA 2735

LN: Lymph Nodes

LncRNA: Long non-coding RNA

LNM: Lymph Node Metastasis

LPS: Lipopolysaccharide

LUAD: Lung adenocarcinoma

MAGEA9B: Melanoma-associated Antigen 4 family member A9B

MAPK: Mitogen-Activated Protein Kinase

MC: Mammary Cancer

MDR: Multidrug Resistance

MEK: Mitogen-Activated Protein Kinase (MAPK) kinase

mGluR1: metabotropic Glutamate Receptor 1

miR: Micro-RNA

MLL: Mixed Lineage Leukemia

MMCs: Mouse Mesangial Cells

MMP1: Matrix Metallopeptidase 1

MMP13: Matrix Metallopeptidase 13

MMP3: Matrix Metallopeptidase 3

MP: Macrophages

mTOR: mechanistic Target Of Rapamycin

MYBL2: Myeloblastosis oncogene-like 2

NF-κB: Nuclear Factor Kappa B

NLK: Nemo Like Kinase

NPC: Nasopharyngeal Carcinoma

NSCLC: Non-Small Cell Lung Cancer

NSCs: Neural Stem Cells

OA: Osteoarthritis

OC: Ovarian Cancer

OS: Osteosarcoma

OSCC: Oral Squamous Cell Carcinoma

OSF: Oral Submucous Fibrosis

OTSCC: Oral Tongue Squamous Carcinoma Cells

OV: Ovarian serous cystadenocarcinoma

PA: Primary Aldosteronism

PAAD: Pancreatic adenocarcinoma

PAS: Polyadenylation Sites

PBX2: Pre-B cell leukemia homeobox 2

PC: Pancreatic Cancer

PCa: Prostate Cancer

PDAC: Pancreatic Ductal Adenocarcinoma

PDGFA: Platelet-Derived Growth Factor subunit A

PD-L1: Programmed Death-Ligand 1

PEPCK: PhosphoEnolPyruvate CarboxyKinase

PI3K: PhosphoInositide 3-Kinase

PPAR: Peroxisome Proliferator-Activated Receptor

PRAD: Prostate adenocarcinoma

PRR20A: Proline-rich protein 20A

PRRS41: Serine protease 41

PRTs: Pineal Region Tumors

Psip1: PC4 and SF2 interacting protein 1

PTBP1: Polypyrimidine Tract Binding Protein 1

PTC: Papillary Thyroid Cancer

PTEN: Phosphatase And Tensin Homologous Protein

RA: Rheumatoid Arthritis

RASFs: RA Synovial Fibroblasts

RB: Retinoblastoma

RBM22: RNA Binding Motif Protein 22

RCC: Renal Cell Carcinoma

RHOXF2B: Rhox Homeobox Family Member 2B

RNAseq: RNA sequencing

RND3: Rho family GTPase 3

RP1-170O19.14: Retinitis Pigmentosa-1-170O19.14

RT-PCR: Real-Time Polymerase Chain Reaction

SCLC: Small Cell Lung Cancer

SEPT14P1: Septin 14 pseudogene 1

SFRP1: Secreted Frizzled-Related Protein 1

SGK1: Serum/Glucocorticoid Regulated Kinase 1

SM: Solamargine

SMARCE1: SWI/SNF related Matrix associated Actin dependent Regulator of Chromatin subfamily E member 1

SNAR-A1: Small NF90 (ILF3) Associated RNA A1

SNORA31: Small Nucleolar RNA, H/ACA Box 31

SNP: Single Nucleotide Polymorphism

snRNA: Small Nuclear RNA

SP-C: Surfactant protein-C

SPHAR: S-phase response

SRF: Serum Response Factor

SSc: Systemic Sclerosis

STAT3: Signal Transducer and Activator of Transcription 3

STC1: Stanniocalcin 1

TAF: Tenofovir Alafenamide

TAF15: TATA-box binding protein associated factor 15

TGFBR: Transforming Growth Factor Beta Receptor

TGFBRAP1: Transforming Growth Factor Β Receptor-Associated Protein 1

TIME: Tumor Immune MicroEnvironment

TM4SF20: Transmembrane 4 L Six Family Member 20

TME: Tumor Microenvironment

TMZ: Temozolomide

TNF-α: Tumor Necrosis Factor-α

TRAP: Tartrate-Resistant Acid Phosphatase

TRIM53CP: Tripartite Motif containing 53C Pseudogene

TRMT112P4: tRNA methyltransferase subunit 11-2 pseudogene 4

TSCC: Tongue Squamous Cell Carcinoma

TTC31: Tetratricopeptide Repeat Domain 31

TWIST1: Twist family bHLH transcription factor 1

US: United States

VEGF: Vascular Endothelial Growth Factor

VEGFA: Vascular Endothelial Growth Factor A

VIM: Vimentin

WDR5: WD Repeat containing protein 5

Wnt: Wingless-related integration site

ZEB1: Zinc Finger E-Box Binding Homeobox 1

ZEB2: Zinc Finger E-Box Binding Homeobox 2

ZFHX4-AS1: Zinc Finger Homeobox 4- antisense1
